# MHCII-Mediated Dialog between Group 2 Innate Lymphoid Cells and CD4^+^ T Cells Potentiates Type 2 Immunity and Promotes Parasitic Helminth Expulsion

**DOI:** 10.1016/j.immuni.2014.06.016

**Published:** 2014-08-21

**Authors:** Christopher J. Oliphant, You Yi Hwang, Jennifer A. Walker, Maryam Salimi, See Heng Wong, James M. Brewer, Alexandros Englezakis, Jillian L. Barlow, Emily Hams, Seth T. Scanlon, Graham S. Ogg, Padraic G. Fallon, Andrew N.J. McKenzie

**Affiliations:** 1 MRC Laboratory of Molecular Biology, Francis Crick Avenue, Cambridge, CB2 0QH, UK; 2 MRC Human Immunology Unit, NIHR Biomedical Research Centre, University of Oxford, John Radcliffe Hospital, OX3 9DS, UK; 3 Trinity Biomedical Sciences Institute, Trinity College Dublin, Dublin 2, Ireland; 4 National Children’s Research Centre, Our Lady’s Children’s Hospital, Crumlin, Dublin 12, Ireland; 5 Institute of Molecular Medicine, Trinity College Dublin, Dublin 2, Ireland; 6 Institute of Infection, Immunity and Inflammation, GRBC, University Place, Glasgow, G12 8TA, UK

## Abstract

Group 2 innate lymphoid cells (ILC2s) release interleukin-13 (IL-13) during protective immunity to helminth infection and detrimentally during allergy and asthma. Using two mouse models to deplete ILC2s in vivo, we demonstrate that T helper 2 (Th2) cell responses are impaired in the absence of ILC2s. We show that MHCII-expressing ILC2s interact with antigen-specific T cells to instigate a dialog in which IL-2 production from T cells promotes ILC2 proliferation and IL-13 production. Deletion of MHCII renders IL-13-expressing ILC2s incapable of efficiently inducing *Nippostrongylus brasiliensis* expulsion. Thus, during transition to adaptive T cell-mediated immunity, the ILC2 and T cell crosstalk contributes to their mutual maintenance, expansion and cytokine production. This interaction appears to augment dendritic-cell-induced T cell activation and identifies a previously unappreciated pathway in the regulation of type-2 immunity.

## Highlights

• Genetic ablation of ILC2s impairs type-2 immunity
• MHCII-expressing ILC2s potentiate Th2 responses
• IL-2 from T cells promotes ILC2 proliferation and expression of type-2 cytokines
• MHCII and IL-13 expression by ILC2s is important for *N. brasiliensis* expulsion

## Graphical Abstract

**Figure:**
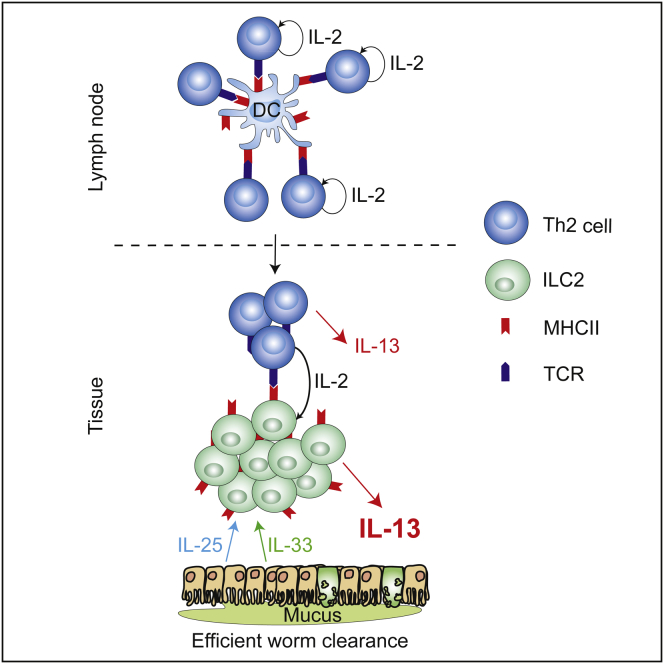

## Teaser

Type-2 innate lymphoid cells proliferate and release interleukin-13 during protective immunity to helminth infection and detrimentally during allergy and asthma. McKenzie and colleagues establish that these activities are potentiated through an MHC class II-mediated dialogue with T cells.

## Introduction

Group 2 immunity is believed to have evolved to combat parasitic helminth infection, but also contributes to wound healing. These responses are characterized by adaptive T helper 2 (Th2) cells expressing interleukin-4 (IL-4), IL-5, and IL-13, B cells secreting immunoglobulin E (IgE), eosinophils, and mast cells. Innate lymphoid cells (ILCs) also play key roles in type-2 responses by producing high amounts of type-2 cytokines (Moro et al., 2010, Neill et al., 2010, Price et al., 2010). ILC2s arise rapidly during helminth challenge, preceding the expansion of the adaptive Th2 response. This raises the question of whether ILC2s contribute to the initiation, polarization, or potentiation of adaptive immunity. Unlike type-1 responses, characterized by Th1 cells expressing interferon-γ (IFN-γ), where macrophages and dendritic cells provide the Th1-cell-inducing factor IL-12, the pathways that elicit and potentiate Th2 cells and type-2 immunity are less well defined. Although IL-4 is a key factor in Th2 cell differentiation, type-2 immunity continues in its absence, indicating additional routes of activation (Forbes et al., 2010, Kopf et al., 1993). IL-25 and IL-33 have been reported to induce T cell expression of type-2 cytokines, and it is these two epithelium-derived factors that potently induce ILC2s at the initiation of type-2 immunity (Moro et al., 2010, Neill et al., 2010). A potential role for ILC2s in influencing the adaptive type-2 response was indicated in an early report demonstrating that a non-B/non-T cell population (later called ILC2) was capable of biasing T cells to a more type-2 phenotype (Fallon et al., 2006). Later, the transfer of ILC2s into IL-13-deficient mice, which displayed reduced T cell responses following helminth infection, was shown to restore Th2 cell responses in vivo (Neill et al., 2010). More recently, ILC2-produced IL-13 has been linked to the migration of dendritic cells (DCs) and the support of Th2 cell differentiation (Halim et al., 2014).

Previous studies have demonstrated that MHCII-expressing conventional antigen-presenting DCs play a critical role in generating type-2 responses (Hammad et al., 2010, Phythian-Adams et al., 2010). MHCII is also expressed on B cells, plasmacytoid DCs, and IFN-γ-producing killer DCs (IKDCs), though in lower amounts than on conventional DCs (Chan et al., 2006, Taieb et al., 2006). More controversial reports advocated a role for basophils in antigen presentation (Perrigoue et al., 2009, Sokol et al., 2009, Yoshimoto et al., 2009). However, the generation of basophil null mice failed to show any major defects in primary Th2 cell responses (Ohnmacht et al., 2010). Thus, although basophils express MHCII, the key function of this molecule in their biology remains to be fully elucidated.

MHCII is expressed on ILC2s and ILC3s (Hepworth et al., 2013, Neill et al., 2010). MHCII on ILC3s, in the absence of the costimulatory molecules CD80 and CD86, resulted in ILC3-mediated suppression of intestinal immune responses against commensal bacteria (Hepworth et al., 2013). However, the role of MHCII on ILC2s remains to be fully characterized. Here we introduce two complementary in vivo models for ILC2 depletion and demonstrate the importance of ILC2s for the efficient development of rapid Th2 cell responses during the expulsion of the parasitic worm *Nippostrongylus brasiliensis*.

## Results

### ILC2s Are Required for Efficient Development of Th2 Cells during *N. brasiliensis* Infection

**Figure 1:**
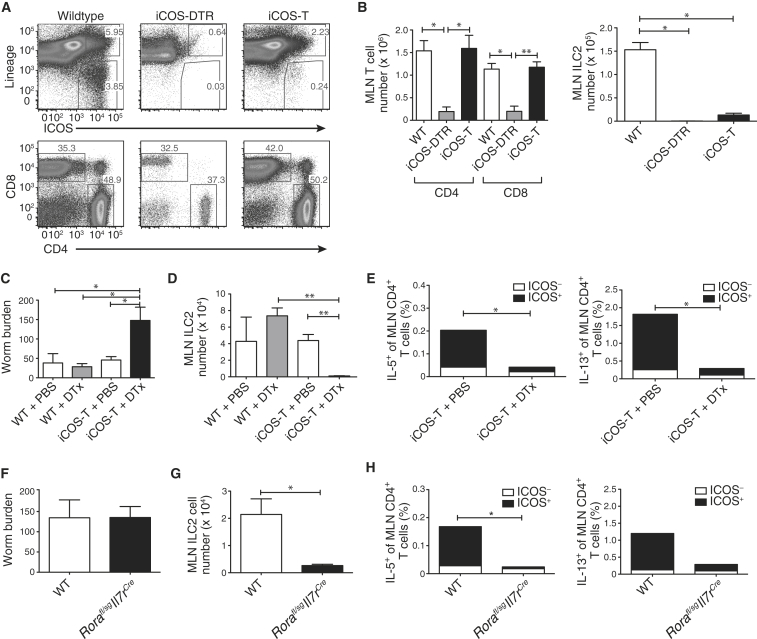
ILC2 Ablation Leads to Impaired Th2 Responses to *N. brasiliensis* (A) ILC2 ablation. Representative flow cytometric analysis of MLN from WT, iCOS-DTR (without *Cd4*^Cre^), or iCOS-T mice (with *Cd4*^Cre^), treated with three daily doses of DTx (25 ng/g bodyweight) and IL-33. (B) Numbers of T cells and ILC2s in MLN of mice, treated daily with 3 doses of DTx and IL-33. (C) Worm burden in mice at 5 days postinfection (d.p.i). iCOS-T mice treated daily with DTx (15 ng/g bodyweight, i.p.). (D) ILC2s in mice 5 d.p.i. (E) CD4^+^ T cells producing IL-5 and IL-13. Statistical analysis performed between CD4^+^ICOS^+^ T cell populations (black bars). (F) Worm burden in *Rora*^fl/sg^*Il7r*^Cre^ mice 5 d.p.i. (G) ILC2 numbers in *Rora*^fl/sg^*Il7r*^Cre^ mice 5 d.p.i. (H) CD4^+^ T cells producing IL-5 and IL-13. Statistical analysis performed between CD4^+^ICOS^+^ T cell populations (black bars). Data are representative of at least two independent experiments with three (A and B) or five (C–H) mice per group and bar graphs represent mean ± SEM. ^∗^p < 0.05 and ^∗∗^ < 0.001. See also Figures S1 and S2.

We have shown that mice lacking the IL-25 receptor fail to induce ILC2s efficiently during *N. brasiliensis* infection. Concurrently, the impaired Th2 cell responses that were observed in these mice implied a role for ILC2s in promoting adaptive immunity (Neill et al., 2010). To define the roles of ILC2s and Th2 cells during a type-2 immune response, we generated two complementary mouse models to enable ILC2 ablation during helminth infection. First, we generated a mouse strain in which ILC2s can be depleted temporally by the administration of diphtheria toxin (DTx). As inducible T cell costimulator (ICOS) is expressed preferentially on both T cells and ILC2s, we inserted a floxed DTx receptor (DTR) gene into the *Icos* locus (resulting in a null allele) enabling the CD4-cre-mediated excision of the DTR gene from T cells but its retention in ILC2s (see Figures S1A–S1C available online). Thus, the treatment of inducible ICOS-diphtheria toxin receptor (iCOS-T) mice with DTx allowed us to selectively deplete ILC2s while sparing T cells (Figures 1A and 1B).

After *N. brasiliensis* infection and DTx treatment, we observed a higher worm burden at day 5 after infection in ILC2-depleted iCOS-T mice compared to controls (Figure 1C), correlating with the specific depletion of ILC2s (Figure 1D), but maintenance of other cell populations (Figures S1H and S1I). Notably, we also observed that ILC2-depleted iCOS-T mice exhibited a substantial decrease in IL-5- and IL-13-producing CD4^+^ T cells (Figure 1E). Because this deficit primarily affected ICOS^+^CD4^+^ T cells, we explored the possibility that ICOS haploinsufficiency might affect CD4^+^ T cell function. ICOS expression on CD4^+^ T cells from iCOS-T mice was reduced as compared to controls (Figure S1D), but T cell-intrinsic defects were not observed following IL-33 administration or *N. brasiliensis* infection (Figures S1E and S1F). The impaired Th2 cell response might be explained through an off-target effect of DTx on ICOS^+^CD4^+^ T cells. While restimulation of ovalbumin-primed splenocytes in vitro in the presence of DTx suggested that this was unlikely (Figure S1G), we generated another strain of ILC2-deficient mice and repeated the infection studies.

The transcription factor retinoid-related orphan receptor alpha (RORα) is critical for ILC2 development (Wong et al., 2012). We have established a conditionally targeted *Rora*^fl/sg^ mouse that when intercrossed with IL-7 receptor (IL-7R)-cre (restricted to the lymphoid lineage) mice yields an ILC2-deficient mouse strain in which other lineages are unaffected (Figures S2A–S2C). *Rora*^fl/sg^*Il7r*^Cre^ ILC2-deficient mice were infected with *N. brasiliensis* and cytokine expression was determined after 5 days. Although we observed little difference in worm burden at day 5 compared to controls (Figure 1F), this strain does show delayed worm expulsion (Figure S2D). The *Rora*^fl/sg^*Il7r*^Cre^ ILC2-deficient mice had reduced ILC2s (Figure 1G) and a dramatic deficit in IL-5 and IL-13-producing ICOS^+^CD4^+^ T cells (Figure 1H).

Thus, in two distinct ILC2-deficient mouse strains in which ILC2 ablation is accomplished through discrete pathways, we observed concordant in vivo defects in CD4^+^ Th2 cells. These data support an in vivo interplay between ILC2s and CD4^+^ T cells, and we proceeded to investigate the potential role of ILC2-expressed MHCII in this interaction.

### MHCII Is Expressed, but Also Acquired, by ILC2s

**Figure 2:**
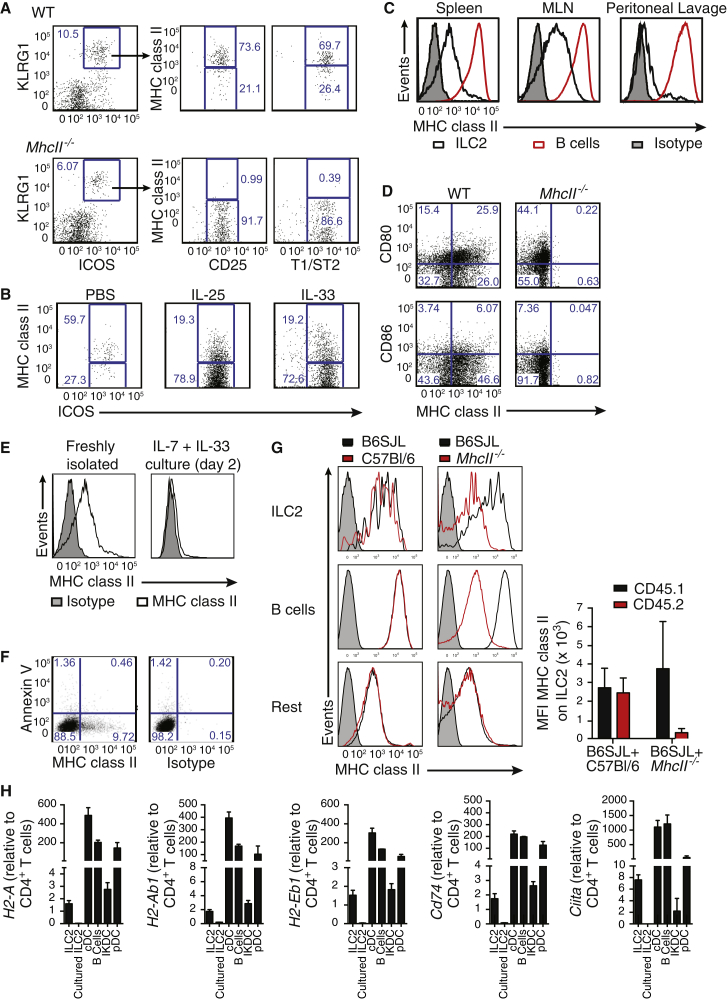
MHCII Is Expressed by ILC2s (A) Representative flow cytometry of MHCII on ILC2s from the MLN of naive mice. Plots are gated on lin^–^ cells. (B) MHCII expression by ILC2s from the MLN of mice treated i.p. as indicated. Plots are gated on lin^–^ cells. (C) MHCII expression on IL-33-elicited ILC2s and B cells from the indicated tissues. (D) CD80 and CD86 expression on Lin^–^ICOS^+^ MLN cells from IL-33-treated mice. (E) MHCII expression on IL-25-elicited ILC2s cultured as indicated. (F) Annexin V and MHCII expression on IL-33-elicited lymph node ILC2s cultured for 48 hr. (G) Chimeric mice were generated by mixing B6SJL (CD45.1^+^) bone marrow with either C57Bl/6 or *MhcII*^−/−^ bone marrow (CD45.2^+^) in a 1:1 ratio. MHCII expression was determined following three doses of IL-33 (i.p.). Graph shows mean fluorescence intensity (MFI) of MHCII expression by ILC2s. (H) Quantitative RT-PCR for MHCII (*H2-Aa*, *H2-Ab1*, or *H2-Eb1*), *Cd74* and *Ciita* gene expression from the indicated populations. ILC2s are from the MLN of IL-33-treated mice. Data are representative of at least two independent experiments with three mice per group. Bar graphs represent mean ± SEM. See also Figure S3.

We confirmed MHCII expression on ∼50%–70% of naive wild-type (WT) ILC2s (Lin^–^Klrg1^+^CD25^+^ST2^+^ICOS^+^Sca1^hi^GATA3^+^IL-13^+^) and those elicited in response to IL-25 or IL-33, as compared to ILC2s from MHCII-deficient mice (*H2-Ab1*^*−/−*^, referred to throughout as *MhcII*^*−/−*^) (Figure 2A and 2B; Figures S3A–S3C). Expression of MHCII on ILC2s was at lower amounts than that on B cells (Figure 2C). Lymph node, spleen, and Peyer’s patch-derived ILC2s expressed MHCII, but ILC2s from the peritoneal lavage, bronchoalveolar lavage, and lung had considerably lower frequency of MHCII expression (Figure 2C; Figure S3D and S3E). A proportion of ILC2s also expressed the costimulatory molecule CD80 and to a lesser extent CD86 (Figure 2D), which are required, in combination with MHCII, for T cell activation and survival. Thus ILC2s express MHCII on their surface and have the potential to interact directly with CD4^+^ T cells via this molecule.

ILC2 culture, for as little as 2 days, resulted in loss of cell surface expression of MHCII and by day 6 of culture only ∼10% of ILC2s retained MHCII expression (Figure 2E; Figure S3F). Annexin V staining confirmed that this was not due to selective apoptosis of MHCII^+^ cells (Figure 2F), raising the possibility that the presence of MHCII on ILC2s might arise through trogocytosis from antigen-presenting cells (APCs) (Wetzel et al., 2005) and that its loss from ILC2s was the result of membrane turnover. Trogocytosis of MHCII by ILC2s was assessed by generating mixed bone-marrow chimeras using CD45.1^+^ WT and CD45.2^+^ MHCII-deficient cells. Low amounts of MHCII dressing were detected on the ILC2s originating from the MHCII-deficient bone marrow, and this was equivalent to the amount of MHCII acquisition observed on other populations, including B cells (Figure 2G; Figure S3G). Cross-dressing did not account for all MHCII on the surface of ILC2s and, furthermore, ILC2s (confirmed as GATA3^+^ and CD11c^–^ compared to DC populations; Figure S3H) expressed mRNA encoding H2-Aa, H2-Ab1, and H2-Eb1, invariant MHCII chaperone CD74, and the MHC transcriptional activator CIITA (Figure 2H). Notably, downregulation of these MHC-related genes coincided with the loss of cell surface MHCII following in vitro culture. Thus, ILC2s express endogenous MHCII and might also acquire this molecule by trogocytosis.

### ILC2 Expression of MHCII Is Important for the IL-13-Dependent Expulsion of *N. brasiliensis*

To investigate the biological importance of MHCII expression by ILC2s in vivo, we assessed the effect of deleting MHCII from ILC2s during an *N. brasiliensis* worm infection. Resolution of *N. brasiliensis* infection is highly dependent on ILC2s, which provide the IL-13 that induces the intestinal mucus production and muscular contraction characteristic of the “weep and sweep” response (Fallon et al., 2002). IL-13-deficient hosts (in which the *Il13* gene has been replaced with the eGFP gene [Neill et al., 2010]) are impaired in their ability to expel *N. brasiliensis*, but parasite clearance can be rescued by transferring WT IL-13-sufficient ILC2s into these hosts.

**Figure 3:**
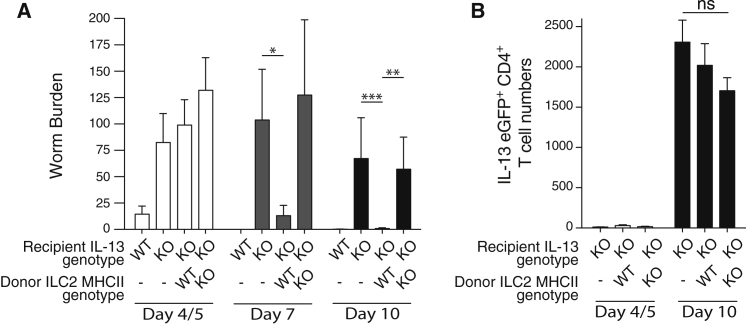
MHCII-Deficient ILC2s Fail to Rescue *N. brasiliensis* Expulsion in IL-13-Deficient Hosts (A) Worm burden in IL-13-deficient hosts following transfer of ILC2s from IL-25-treated WT or *MhcII*^*−/−*^ donors. Data are pooled from four independent experiments with five or six mice in each group per experiment. ^∗^p < 0.05, ^∗∗^p < 0.01, and ^∗∗∗^p < 0.001. (B) Number of *Il13*^egfp/egfp^ CD4^+^ T cells in MLN following infection. Data are representative of three independent experiments with five to six mice per group. Bar graphs represent mean ± SEM. See also Figure S4.

We tested the function of MHCII expression on ILC2s by transferring MHCII-deficient ILC2s into the *N. brasiliensis*-infected IL-13-deficient mice. The transferred ILC2s could be identified in the IL-13-deficient recipients due to expression of IL-13 protein (Figure S4A). Despite having similar intestinal parasite burdens at day 4/5, only IL-13-deficient hosts receiving WT ILC2s expelled their worms efficiently by day 7 (Figure 3A). By contrast, IL-13-deficient hosts receiving MHCII-deficient ILC2s displayed delayed parasite expulsion (Figure 3A). Notably, IL-13-deficient hosts receiving MHCII-deficient ILC2s showed impaired worm expulsion despite normal, albeit IL-13-deficient, T cell induction (Figure 3B), presumably driven by endogenous MHCII-sufficient APCs. Importantly, we found no intrinsic deficit in the ability of MHCII-deficient ILC2s to produce IL-13 as compared to controls (Figures S4B and S4C). These results suggest a dialog between ILC2s and T cells that is required to induce sufficient IL-13-positive ILC2s.

### ILC2 MHCII Contributes to Antigen-Induced Activation of CD4^+^ T Cells

**Figure 4:**
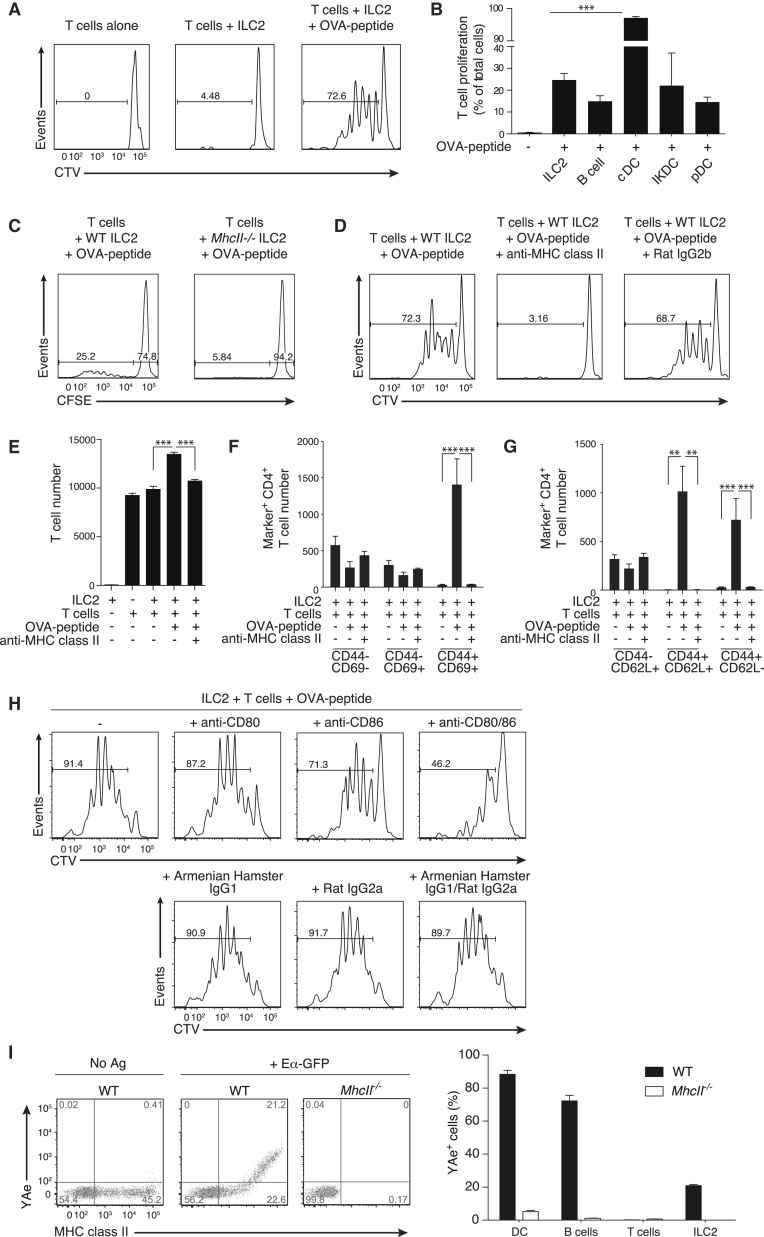
Antigen-Dependent Activation of T Cells by ILC2s Requires MHCII (A) Cell trace violet (CTV)-labeled OTIITg CD4^+^ T cells were cultured in a 1:1 ratio with IL-33-elicited ILC2s, pulsed with or without OVA-peptide. (B) Antigen-dependent OTIITg CD4^+^ T cell proliferation induced by the indicated populations. (C) CFSE-labeled OTIITg CD4^+^ T cell proliferation, as indicated. (D) CTV-labeled OTIITg CD4^+^ T cell proliferation, as indicated. (E) OTIITg CD4^+^ T cell numbers in cocultures, as indicated. (F and G) The number of activated OTIITg CD4^+^ T cells (CD44^+^CD69^+^ or CD44^+^CD62L^–^) following coculture with antigen-pulsed ILC2s. (H) CTV-labeled OTIITg CD4^+^ T cell proliferation, as indicated. Data are representative of three independent experiments with two or three mice per group. (I) Detection of processed and presented Eα peptide on ILC2s. Data are representative of two independent experiments. ILC2s were elicited using IL-33 and isolated from mesenteric, para-aortic, and inguinal lymph nodes. Bar graphs represent mean ± SEM. See also Figure S5.

We next investigated the potential role for MHCII on ILC2s. Purified C57Bl/6 ILC2s were cultured with ovalbumin (OVA)-specific OTII transgenic (Tg) C57Bl/6 CD4^+^ T cells (Barnden et al., 1998) in the presence or absence of OVA-derived peptide. ILC2s induced in vivo with IL-25 or IL-33 and preincubated with OVA-peptide induced similar T cell proliferation (Figure 4A; Figure S5A). In contrast, CD4^+^ T cells alone, or cocultures of T cells and ILC2s in the absence of OVA peptide, failed to induce T cell proliferation. ILC2s were purified by flow cytometry to avoid DC cross-contamination (Figure S5B) and limiting dilution analysis confirmed that the contribution of contaminating DCs could be discounted (data not shown). Similar results were obtained with ILC2s purified from BALB/c mice in the presence of BALB/c DO11.10Tg CD4^+^ T cells (Figure S5C). An irrelevant peptide derived from myelin proteolipid protein failed to stimulate DO11.10Tg T cell proliferation (Figure S5D). ILC2s that had downregulated surface MHCII expression following in vitro culture (Figure 2E) also failed to stimulate DO11.10Tg T cell proliferation (Figure S5E). The inclusion of DCs in these cultures indicated that in vitro cultured ILC2s did not actively impair T cell proliferation (Figure S5E). The peptide-presenting capacity of ILC2s was significantly lower than that of CD11c^+^ DCs but was equivalent to that observed for naive B cells, IKDCs, and plasmacytoid DCs (Figure 4B).

Unlike ILC2s from WT mice, MHCII-deficient ILC2s failed to induce OTIITg T cell proliferation (Figure 4C). To confirm that the absence of MHCII had not altered the developmental state of ILC2s in the MHCII gene-targeted mice, we also demonstrated that a neutralizing anti-MHCII antibody, but not an isotype control, prevented proliferation of OTIITg T cells cocultured with peptide-pulsed ILC2s (Figures 4D and 4E; Figure S5F). Antigen-dependent T cell activation by ILC2s was further confirmed by increased numbers of activated CD4^+^ T cells (CD44^+^CD69^+^CD62L^−^, Figures 4F and 4G). These data demonstrate that ILC2 MHCII ligation induced the expansion and activation of CD4^+^ T cells.

Because ILC2s express the costimulatory molecules CD80 and CD86 (Figure 2D), we examined the role for these molecules by using blocking antibodies in vitro. Although individual blockade of CD80 or CD86 had little effect on T cell proliferation (Figure 4H), a combination of both antibodies inhibited T cell proliferation as compared to controls (Figure 4H). Antibody blocking was ineffective in BALB/c DO11.10Tg cocultures (data not shown), suggesting potential effects of TCR affinity on the requirement for costimulatory signals. Thus, the expression of the costimulatory molecules CD80 and CD86 by ILC2s provides an additional signal for the proliferation of OTIITg T cells in the context of peptide.

### Murine ILC2s Endocytose, Process, and Present Antigen

ILC2s endocytosed soluble antigen in vitro dose dependently (Figures S5G–S5I) and also degraded ovalbumin-DQ (OVA-DQ), a self-quenched conjugate of ovalbumin protein that fluoresces when cleaved (Figures S5H and S5I). Furthermore, in vivo processed OVA-DQ fluorescence was observed in bronchoalveolar lavage and lung-derived ILC2s following intranasal antigen administration (Figures S5J and S5K). However, neither ILC2s loaded in vitro with OVA, or sorted OVA-DQ-positive ILC2s, loaded in vivo, induced transgenic T cell proliferation in coculture (Figures S5L and S5M). To determine whether this resulted from an inability of ILC2s to present the processed antigen, we cultured ILC2s in the presence of an E-alpha (Eα)-green fluorescent protein (GFP) fusion protein and used an Eα-specific antibody to detect an Eα-derived peptide bound in the context of MHC (I-A^b^) (Pape et al., 2007). ILC2s were incubated with Eα-GFP and after 20 hr stained for the presence of Eα-derived peptide. Notably, a proportion of the WT, but not MHC-deficient, ILC2s stained positive for the presence of MHCII-bound Eα-peptide as did DCs and B cells (Figure 4I). Thus, ILC2s can process and present antigen, albeit to a lesser extent than professional APCs, but not sufficiently to elicit T cell proliferation in the in vitro assays tested.

### ILC2s Enter an Antigen-Dependent Dialog with CD4^+^ T Cells

**Figure 5:**
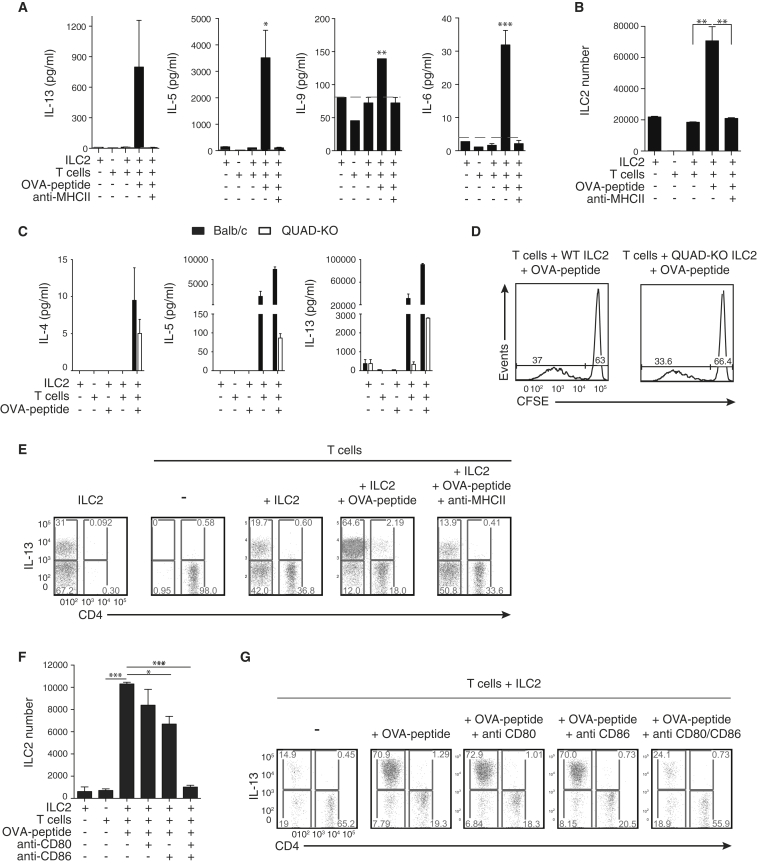
ILC2s Enter Antigen-Dependent Dialog with CD4^+^ T Cells (A) Cytokines in supernatants of cocultures containing peptide-pulsed ILC2s and OTIITg CD4^+^ T cells. Dotted line indicates limit of detection for each assay. (B) ILC2 number following coculture with OTIITg CD4^+^ T cells, as indicated. (C) Cytokine production in supernatants of WT (BALB/c) or QUAD-KO ILC2s and DO11.10Tg T cell cocultures, as indicated. (D) CFSE-labeled DO11.10Tg T cell proliferation following coculture with QUAD-KO ILC2s, as indicated. (E) Intracellular IL-13 staining following ILC2:OTIITg CD4^+^ T cell coculture, as indicated. (F) ILC2 number following coculture with OTIITg CD4^+^ T cells, as indicated. (G) Intracellular IL-13 staining following ILC2:OTIITg CD4^+^ T cell coculture, as indicated. Data are representative of three independent experiments with two or three mice per group. MLN ILC2s were elicited using IL-33 (A, B, E–G) or IL-25 (C and D). Bar graphs represent mean ± SEM. See also Figure S6.

We next examined whether ILC2s polarize OTIITg CD4^+^ T cells toward a Th2 phenotype. Supernatants from OTIITg T cells cocultured with OVA peptide-pulsed ILC2s contained markedly increased amounts of type-2 cytokines IL-5, IL-6, IL-9, and IL-13, but little IFN-γ or IL-17A, as compared to controls lacking peptide (Figure 5A; Figure S6A). This elicitation of type-2 cytokine expression was blocked by neutralizing anti-MHCII antibody (Figure 5A). Notably, this antigen- and MHCII-dependent increase in type-2 cytokines correlated with enhanced ILC2 numbers (Figure 5B).

The source of type-2 cytokines was investigated with cocultures containing either WT ILC2s or ILC2s harvested from mice with a combined deficiency for IL-4, IL-5, IL-9, and IL-13 (QUAD-KO) (Fallon et al., 2002). Coculture of WT ILC2s and DO11.10Tg T cells resulted in IL-5 and IL-13 expression that increased with the inclusion of peptide (Figure 5C). Comparison to parallel cultures, in which QUAD-KO ILC2s were combined with transgenic T cells, demonstrated that the majority of the IL-5 and IL-13 observed in the WT cultures was ILC2-derived (Figure 5C) and that type-2 cytokine production was only induced in T cells following the addition of peptide (Figure 5C). The absence of ILC2-derived type-2 cytokines had no effect on the proliferative potential of T cells (Figure 5D). Intracellular cytokine staining confirmed that ILC2s were the major source of type-2 cytokines following coculture in the presence of OVA peptide (Figure 5E; Figure S6B). Blocking antibodies targeting MHCII (Figure 5E; Figure S6B) or CD80 and CD86 (Figures 5F and 5G) impaired ILC2 expansion and type-2 cytokine expression. Together, these data highlight that ILC2s and T cells enter an antigen-dependent crosstalk that results in their subsequent activation and potentiation of type-2 immune responses and requires the expression of both MHCII and the costimulatory molecules CD80 and CD86 on ILC2s.

### Crosstalk between ILC2s and CD4^+^ T Cells Requires IL-2

**Figure 6:**
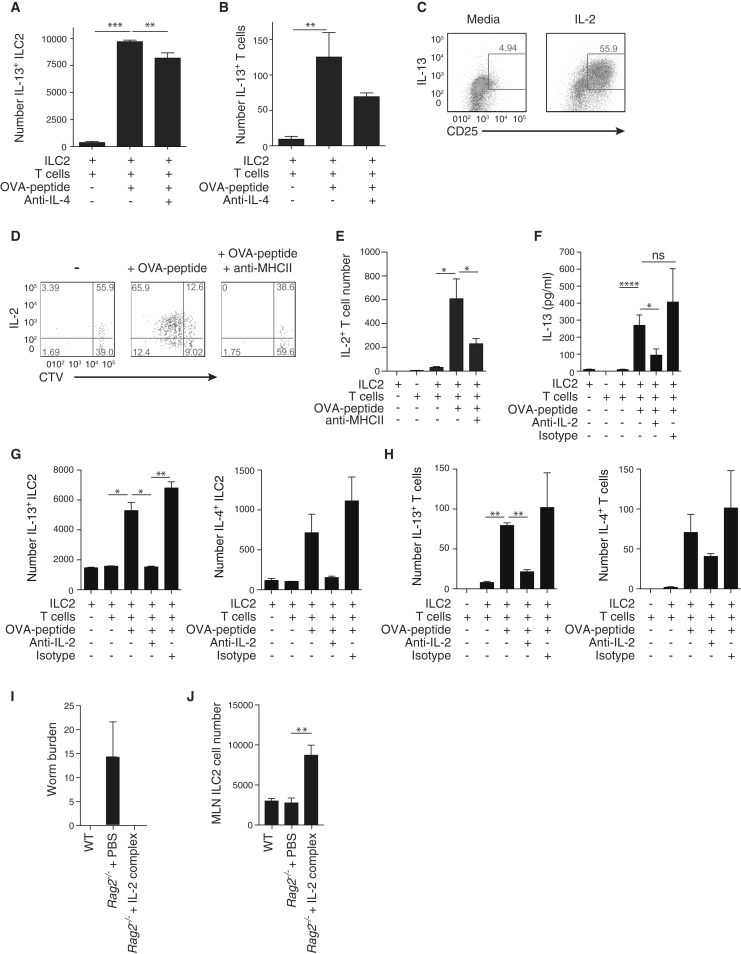
IL-2 Is Required for the Antigen-Dependent Dialog between ILC2s and T Cells (A and B) Number of IL-13-expressing ILC2s (A) and T cells (B) from ILC2:OTIITg CD4^+^ T cell cocultures in the presence of IL-4-blocking antibodies. (C) Surface CD25 and intracellular IL-13 expression by ILC2s stimulated with IL-2 for 72 hr. (D) Intracellular IL-2 staining of CTV-labeled OTIITg CD4^+^ T cells following coculture with OVA-peptide-loaded WT ILC2s. (E) Number of IL-2-expressing T cells from ILC2:OTIITg CD4^+^ T cell cocultures in the presence of MHCII-blocking antibodies. (F) IL-13 concentration in supernatants from ILC2:OTIITg CD4^+^ T cell cocultures in the presence of blocking anti-IL-2 antibodies. (G and H) Number of cytokine expressing ILC2s (G) and OTIITg CD4^+^ T cells (H) in the presence of IL-2 blocking antibodies. (I) Worm burdens and (J) ILC2s in *Rag2*^−/−^ mice 10 d.p.i. with *N. brasiliensis* and treated as indicated. Lymph node ILC2s were elicited with IL-33. Bar graphs represent mean ± SEM. Data are representative of three independent experiments with two or three mice per group (A–H) or from a single experiment with five or six mice per group (I and J). Bar graphs represent mean ± SEM.

We next sought to identify the secreted factors required for ILC2s and CD4^+^ T cell interaction. Notably, although IL-4, a factor linked to Th2 cell polarization, is not always detectable in ILC2s (Fallon et al., 2006, Neill et al., 2010), we observed that IL-4 production from ILC2s was clearly evident in the presence of peptide (Figure 5C; Figures S6B and S6C). Blocking IL-4 in cocultures had only a small effect on IL-13^+^ ILC2 and T cell numbers (Figures 6A and 6B). Thus, IL-4 might play a minor role in altering the cytokine milieu that arises in the MHCII-mediated collaboration between ILC2s and T cells.

The limited effect of IL-4 led us to investigate IL-2, the receptor for which (CD25) is highly expressed on ILC2s. Indeed, culture of ILC2s with IL-2 resulted in increased numbers of IL-13-producing ILC2s (Figure 6C). Intracellular cytokine staining demonstrated an antigen-dependent increase in the number of IL-2-expressing T cells in ILC2 cocultures, which was impaired with anti-MHCII antibodies (Figures 6D and 6E). Critically, IL-2 blockade prevented antigen-induced expression of type-2 cytokines in culture supernatants (Figure 6F), and intracellular cytokine staining confirmed a role for IL-2 in the expression of type-2 cytokines from ILC2s (Figure 6G) and T cells (Figure 6H).

We have shown previously that *Rag2*^−/−^ mice have impaired worm clearance. To determine whether this deficit might be due to insufficient IL-2 production from T cells failing to induce sufficient ILC2s, we infected *Rag2*^−/−^ mice with *N. brasiliensis* while also treating one group with an IL-2-anti-IL-2 antibody complex to facilitate slow release of the active IL-2 molecule. *Rag2*^−/−^ mice sufficient for ILC2s failed to expel their worms efficiently (Figure 6I). By contrast, providing IL-2 resulted in rapid worm expulsion that correlated with elevated ILC2 numbers (Figures 6I and 6J). Thus, the MHCII-, CD80-, CD86-initiated production of IL-4 and IL-2 from ILC2s and T cells coordinates efficient T cell and ILC2 proliferation and type-2 cytokine expression.

### Human ILC2s Express MHCII and Present Antigen to T Cells

In parallel studies, we assessed MHCII expression on purified human lineage^–^IL-7Rα^+^CRTH2^+^ ILC2s (Mjösberg et al., 2011, Salimi et al., 2013) (Figure S6D). Human ILC2s expressed high amounts of HLA-DR, comparable to human monocytes, in conjunction with the costimulatory receptors CD80 and CD86 (Figure S6E). We investigated the ability of human ILC2s to activate human T cell lines with antigen specificity for the common house dust-mite allergen (HDM) Der p 1. Increasing concentrations of whole Der p 1 protein or Der p 1 peptide (DRB15), induced a 100- to 200-fold increase in the expression of IL-4 (Figure S6F), accompanied by a 3-fold increase in IFN-γ production (Figure S6G). Intracellular cytokine staining revealed induction of T cell-derived IL-13 in HLA-DR^+^ ILC2 cocultures, but not those with anti-MHCII or HLA-mismatched ILC2s (Figure S6H). However, ILC2-produced IL-13 was not detected (Figure S6H). Thus, human ILC2s can process and present antigens to T cells thereby initiating cytokine expression.

## Discussion

A critical role for ILC2s in antihelminthic immune responses was demonstrated in mice deficient in IL-33 and IL-25 signaling (Neill et al., 2010) and in RORα-deficient mice, which lack ILC2s (Wong et al., 2012). Transfer of ILC2s into IL-13-deficient, but not Rag2-deficient, hosts was sufficient to restore worm clearance (Neill et al., 2010). This suggested that interactions between IL-13-sufficient ILC2s and T cells, but probably not B cells (Voehringer et al., 2006), were required for the effective clearance of parasitic helminths. The impairment of T cell responses suggested that in addition to conventional T cell priming by DCs, which is essential for efficient Th2 generation (Hammad et al., 2010, Phythian-Adams et al., 2010), ILC2s might also play a previously unanticipated role in the initiation of protective type-2 immunity.

We have generated two ILC2-depletion models to study ILC2 function in vivo. The iCOS-T model relies upon *Icos*-promoter controlled expression of DTR on ILC2s enabling temporal regulation of ILC2 deletion by DTx-administration. In the second model, ILC2s were depleted through IL-7Rcre-mediated excision of a “floxed” exon in the *Rora* gene. In these distinct models, we observed that ILC2 ablation impaired the magnitude of the Th2 response during *N. brasiliensis* infection.

While MHCII-expressing conventional antigen-presenting DCs are essential for activating naive T cell responses (Hammad et al., 2010, Phythian-Adams et al., 2010), MHCII-mediated crosstalk between ILC2s and T cells plays an additional role in potentiating IL-13-dependent *N. brasiliensis* expulsion. T cell-derived IL-2 induces proliferation of ILC2s and elevates their expression of type-2 cytokines. Thus, this reciprocal interaction potentiates both the innate and adaptive sources of type-2 cytokines and provides an important antigen-dependent feedback, whereby regulation of ILC2 proliferation or survival shifts from innate sources of IL-33 and/or IL-25 (Neill et al., 2010) to cytokines provided by an ongoing adaptive response, namely IL-2 and IL-4 derived from T cells.

Several pathways regulate Th2 cell differentiation, including TCR signal strength, ligation of costimulatory molecules on APCs, and availability of cytokines such as IL-2 and IL-4 (Cote-Sierra et al., 2004, Jenkins et al., 2008, Yamane et al., 2005). Coculture of T cells with ILC2s, in the presence of antigen, preferentially induces T cell secretion of IL-5 and IL-13. Notably, we observed relatively low amounts of MHCII expression on ILC2s, compared to DC subsets or B cells. This interaction might contribute to the polarization of the T cell response because suboptimal TCR ligation has been reported to bias Th2 differentiation in vitro (Yamane et al., 2005) and in vivo (Jun et al., 2003). Similarly, components from *Schistosoma mansoni* egg extracts have been demonstrated to condition DCs, resulting in their impaired interaction with T cells and potentially reduced TCR signal strength (Everts et al., 2009). Therefore, ILC2s, unlike other APC, might be preconditioned to polarize T cells toward a Th2 phenotype by virtue of their lower amount of MHCII expression.

Cytokine regulation of Th2 cell differentiation has focused on IL-4 though it is clear that it is not essential for Th2 development in vivo (van Panhuys et al., 2008). Indeed, we found little role for IL-4 in the ILC2-regulated differentiation of Th2 cells. By contrast, IL-2 was critical for the proliferation of Th2 cells in the presence of ILC2s and antigen. IL-2 is expressed by T cells following CD28 ligation (Seder et al., 1994) and plays an important role in Th2 differentiation via STAT5a signaling (Cote-Sierra et al., 2004, Zhu et al., 2003). Notably, we found that the CD28 ligands CD80 and CD86 were expressed on ILC2, suggesting that ILC2s have the capacity to interact with T cells and contribute to their type-2 polarization.

Strikingly, we observed a profound increase in ILC2 proliferation and type-2 cytokine production in T cell cocultures with antigen, which was dependent on T cell-derived IL-2. ILC2s require the common γ-chain (γc) receptor for their development and proliferation, and are absent in γc receptor-deficient mice (Moro et al., 2010, Neill et al., 2010, Price et al., 2010). Furthermore, Moro et al. (2010) showed that ILC2s from fat-associated lymphoid clusters (FALC) expanded in the presence of IL-2, resulting in elevated amounts of cytokine production (Moro et al., 2010). Additionally, IL-2 has also been reported to induce ILC2s to secrete IL-9, which then protects the ILC2s from apoptosis via an autocrine feedback loop (Turner et al., 2013). Therefore, it is possible that IL-2 expressed following antigen-dependent ILC2:T cell interactions induces IL-9 expression and improves ILC2 survival, thereby further biasing toward a type-2 immune response. Certainly, IL-9 was present in the cultures that included T cells and antigen, though we were unable to identify the cellular source.

Despite demonstrating that murine ILC2s can take up and process OVA, and process and present Eα-GFP, we have been unable to demonstrate T cell proliferation. Several studies have shown that cells can acquire MHCII:antigen complexes from antigen-presenting cells (Wetzel et al., 2005). We observed acquisition of MHCII by ILC2s in mixed bone-marrow chimera experiments, which might contribute to their biology. However, where MHCII-deficient ILC2s were transferred into *N. brasiliensis*-infected MHCII-sufficient mice, MHCII trogocytosis was insufficient to restore worm expulsion. In contrast to mouse ILC2s, we found that human ILC2s process whole Der p 1 antigen and present the derived peptides to T cells in vitro. Thus, our existing in vitro cellular proliferation assays appear too insensitive. Previously, human NK cells have been shown to process and present tetanus toxoid and Der p 1 to T cells, but protein antigen from *Mycobacterium leprae* was poorly processed and presented (Roncarolo et al., 1991). It is also noteworthy that many of the antigens associated with type-2 immune responses are either shed by multicellular helminth parasites (Kamata et al., 1995) or are proteins with protease activity, such as Der p 1 or papain (Gough et al., 1999, Halim et al., 2012). It is therefore interesting to speculate that ILC2s might also acquire exogenous peptides from their surroundings (Santambrogio et al., 1999).

This proinflammatory role for MHCII expression on ILC2s supports similar findings for peptide presentation by ILC2s (Mirchandani et al., 2014). By contrast, ILC3s have been reported to suppress intestinal immune responses against commensal bacteria (Hepworth et al., 2013). Notably, ILC2s express the costimulatory molecules CD80 and CD86 on their surface, whereas ILC3s do not (Hepworth et al., 2013), and this might underlie the agonistic role for MHCII on ILC2s.

In addition to antihelminthic immunity, ILC2s have also been implicated in promoting detrimental type-2 immune responses in mouse models of asthma (Barlow et al., 2012, Chang et al., 2011, Monticelli et al., 2011) and allergies (Bartemes et al., 2012, Halim et al., 2012, Salimi et al., 2013). Currently there is little information regarding ILC2s and T cell interactions in allergic models, but similar antigen-dependent ILC2:T cell crosstalk might also be important for the potentiation of type-2 responses in allergy. Indeed, ILC2s are enriched in nasal polyps from patients with chronic rhinosinusitis (Mjösberg et al., 2011), which might arise in response to allergens. We have now demonstrated the ability of human ILC2s to process and present the house-dust-mite allergen Der p 1, to human T cells in vitro in a process that would exacerbate type-2 inflammation. In these human assays, we observed the induction of T cell-derived IL-13, but did not detect ILC2-produced IL-13. It is not clear why this is the case but might be a consequence of the 3- to 4-week culture required to derive sufficient ILC2s for these assays. However, these data support a potential role for human ILC2s in the potentiation of T cell responses during asthma and allergy.

The two mouse models that we describe for the genetic ablation of ILC2s in vivo complement those that have already been reported. The use of the *Il13* gene to drive diphtheria toxin resulted in the deletion of IL-13-producing ILC2s, but other IL-13-producers would also be susceptible to toxin-mediated cell death (Liang et al., 2012). The depletion of ILC2s has also been reported in mice in which *Id2*^CreER^ mediated the excision of a conditional *Gata3* allele (Hoyler et al., 2012); however, it is becoming clear that GATA3 is required for all ILC populations (Serafini et al., 2014). We now show that ILC2 ablation can be regulated temporally in otherwise immune-competent iCOS-T mice. Because it remained possible that the insertion of DTR into the *Icos* locus might alter T cell activation, we also generated *Rora*^fl/sg^*Il7r*^Cre^ mice. Although RORα has been reported previously to play a subordinate role to RORγ in the development of Th17 T cells, a role in Th2 cell activation has not been reported. Thus, the complementary data from these two distinct mouse lines strongly support the role for ILC2s in the progression to adaptive Th2-mediated immunity. These mice should prove useful for defining the roles of ILC2s in further disease models.

Our data demonstrate that ILC2s and T cells cooperate through MHCII-dependent activation to potentiate the type-2 response against *N. brasiliensis* and extend the recently described pathway by which IL-13 from ILC2s can promote DC migration to the draining lymph nodes to stimulate Th2 polarization (Halim et al., 2014). Such functions place ILC2s at a critical point in the transition from the innate type-2 response to adaptive type-2 immunity. Therefore, ILC2s expand initially in response to IL-33 and IL-25 derived from innate cell sources but require adaptive T cell-produced mediators for their maintenance and subsequent potentiation of protective type-2 immunity.

## Experimental Procedures

### Mice

C57Bl/6, *H2-Ab1*^*−/−*^ (*MhcII*^−/−^) (Cosgrove et al., 1991), *Il13*^egfp/egfp^ (Neill et al., 2010), *Il7r*^Cre^ (Schlenner et al., 2010), OTIITgB6 (JAX Laboratories), and Staggerer *Rora*^sg/+^ (JAX Laboratories) mice were on a C57Bl/6 background. *Il13*^tdTomato/+^, *Il4*^gfp/gfp^ (Hu-Li et al., 2001) and *Il4*^−/−^*Il5*^−/−^*Il9*^−/−^*Il13*^−/−^ (QUAD-KO) (Fallon et al., 2002) mice were on a BALB/c background. iCOS-DTR, iCOS-T, and *Rora*^+/flox^*Il7r*^Cre^ mice were generated as described in the Supplemental Experimental Procedures. All mice were bred in a specific pathogen-free facility. BALB/c mice were purchased from Charles River Laboratories as required. In individual experiments all mice were matched for age, gender, and background strain. All animal experiments undertaken in this study were done so with the approval of the UK Home Office.

### IL-25, IL-33, IL-2 Complex and DTx Administration

IL-25 (either 2 μg/mouse or 0.5 μg/mouse of recombinant mouse IL-25 [Centocor] in PBS) was administered daily for 3 days intraperitoneally (i.p., termed IL-25-elicited ILC2s). For i.p. IL-33 administration, 0.5 μg/mouse or 1 μg/mouse recombinant mouse IL-33 (BioLegend) in PBS was administered daily for up to 4 days (termed IL-33-elicited ILC2s). For intranasal IL-33 administrations, mice were anesthetized and 0.4 μg of recombinant protein administered in PBS on 4 consecutive days. IL-2 complexes were prepared by incubating IL-2 and anti-IL-2 mAb (clone JES6) (both from R&D Systems) at a 1:5 w/w ratio for 20 min at 37°C. Complexes were then diluted in PBS and administered i.p. (1 μg IL-2 and 5 μg anti-IL-2 per dose) on days 0, 2, 4, 6, and 8 following *N. brasiliensis* infection. DTx (either 25 ng/g or 15 ng/g body weight, Sigma) was administered daily for up to 5 days i.p. Mice were euthanized 24 hr later and tissues collected for analysis. Control animals received PBS only.

### Bone-Marrow Chimeras

For mixed-chimera experiments, lethally irradiated CD45.1^+^ B6/SJL mice were reconstituted with a 1:1 mixture of bone-marrow cells (2 × 10^6^ cells) containing either CD45.1^+^ B6/SJL and CD45.2^+^ C57Bl/6 or CD45.1^+^ B6/SJL and CD45.2^+^ *MhcII*^*−/−*^ cells. After 10 weeks, mice were given daily doses of recombinant mouse IL-33 (0.4 μg per dose).

### In Vitro ILC2 Cultures with IL-7 and IL-33 or IL-2

For the expansion of ILC2s in vitro from WT mice, ILC2s were FACS purified, as defined by LIN^−^ (a combination of CD3, CD4, CD8α, CD19, B220, CD11c, CD11b, Gr1, FcεR1, CD5, γ/δTCR, NK1.1, and TER119) ICOS^+^. For IL-7/IL-33 in vitro cultures of ILC2s, we followed the protocol as described previously (Neill et al., 2010). Cultures were maintained for between 2 and 6 days. For in vitro IL-2 cultures, ILC2s were FACS purified from the spleen and MLN of IL-25 or IL-33-treated mice, and subsequently cultured at 2.5 × 10^4^ cells per well in the presence or absence of IL-2 (10 ng/ml). Cultures were maintained for 3 days at 37°C.

### In Vitro ILC2s and T Cell Cocultures

Freshly isolated or in vitro cultured ILC2s, from WT, *MhcII*^−/−^, or QUAD-KO mice, were resupended in RPMI 1640 (GIBCO®) supplemented with 2 mM L-glutamine, 50 U/ml penicillin, 50 μg/ml streptomycin, and 10% Hyclone Fetal Bovine Serum (Thermoscientific). Where necessary, ILC2s were pulsed with OVA-peptide (323–339) for 2 hr at 37°C. Subsequently ILC2s were washed thoroughly and 1 × 10^4^ ILC2s were cultured with 1 × 10^4^ labeled CD3^+^CD4^+^ OTIITg or DO11.10Tg splenic T cells, which were sorted by flow cytometry, for 5 days at 37°C, 5% CO_2_. Where indicated, the antibodies M5/114.15.2 (anti-MHCII, eBioscience), 16-10A1 (anti-CD80, BioLegend), and GL-1 (anti-CD86, BioLegend) were added to cultures at a concentration of 1 μg/ml. 11B11 (anti-IL-4, eBioscience) was incubated at a concentration of 10 μg/ml. Where appropriate, blockade of IL-2 was performed with 10 μg/ml of JES6-1A12 (eBioscience) and S4B6 (BD PharMingen). Cocultures containing in vivo-loaded OVA-DQ^+^ ILC2s were prepared by FACS-purifying ILC2s, based on Lin^−^ICOS^+^OVA-DQ^+^ or lineage^−^ICOS^+^OVA-DQ^−^, from the bronchoalveolar lavage of WT mice receiving an intranasal administration of OVA-DQ and IL-33 on 7 consecutive days. Cocultures were setup at a 1:1 ratio with 4 × 10^4^ cells per well. Human samples were taken under GCP guidance with ethical approval of the NRES Committee South Central.

### Helminth Infection and Cell Transfers

Mice were inoculated subcutaneously with 500 viable third-stage *N. brasiliensis* larvae. Where appropriate, FACS-purified ILC2s were transferred by intravenous injection 2 hr after *N. brasiliensis* injections.

### E-alpha:Green Fluorescent Protein Fusion Protein

To assess the ability of ILC2s to present processed antigen, we cultured ILC2s in the presence of 100 μg/ml E-alpha (Eα)-green fluorescent protein (GFP) fusion protein for 20 hr. An Eα-specific antibody (clone YAe) was used to detect an Eα-derived peptide (Eα52-68) bound in the context of MHC (I-A^b^) prior to flow cytometric analysis.

### Statistical Analysis

Graph Pad Prism was used to calculate the SEM when different numbers of data sets existed in each experimental group. When data were normally distributed and when two independent variables were being analyzed, a Kruskal-Wallis one-way ANOVA with Bonferroni post-analysis was performed. In all other instances, statistical differences between groups were calculated with Student’s t test, with *P* < 0.05 considered significant.

## Author Contributions

A.N.J.M., S.H.W, Y.Y.H., and C.J.O conceived the study. C.J.O, S.H.W., J.A.W., J.L.B., Y.Y.H., E.H., A.E., S.T.S., and P.G.F. did the experiments or contributed to experimental design, reagents, and analysis. G.S.O and M.S. performed the human ILC2 experiments. A.N.J.M. wrote the manuscript with contributions from all authors, but primarily from C.J.O and J.A.W.

## Supplemental Information

Document S1. Figures S1–S6 and Supplemental Experimental Procedures

Document S2. Article plus Supplemental Information
