# Supplementary material for: MHCII-Mediated Dialog between Group 2 Innate Lymphoid Cells and CD4+ T Cells Potentiates Type 2 Immunity and Promotes Parasitic Helminth Expulsion
Source: Immunity. 2014 Aug 21;41(2):283–95. doi: 10.1016/j.immuni.2014.06.016 (PMC4148706; doi:10.1016/j.immuni.2014.06.016)
Supplement: Document S1. Figures S1–S6 and Supplemental Experimental Procedures [file mmc1.pdf]

Immunity, Volume *41*

Supplemental Information

MHCII-Mediated Dialog between Group 2 Innate Lymphoid Cells and CD4$^+$ T Cells Potentiates Type 2 Immunity and Promotes Parasitic Helminth Expulsion

Christopher J. Oliphant, You Yi Hwang, Jennifer A. Walker, Maryam Salimi, See Heng Wong, James M. Brewer, Alexandros Englezakis, Jillian L. Barlow, Emily Hams, Seth T. Scanlon, Graham S. Ogg, Padraic G. Fallon, and Andrew N.J. McKenzie

Figure S1

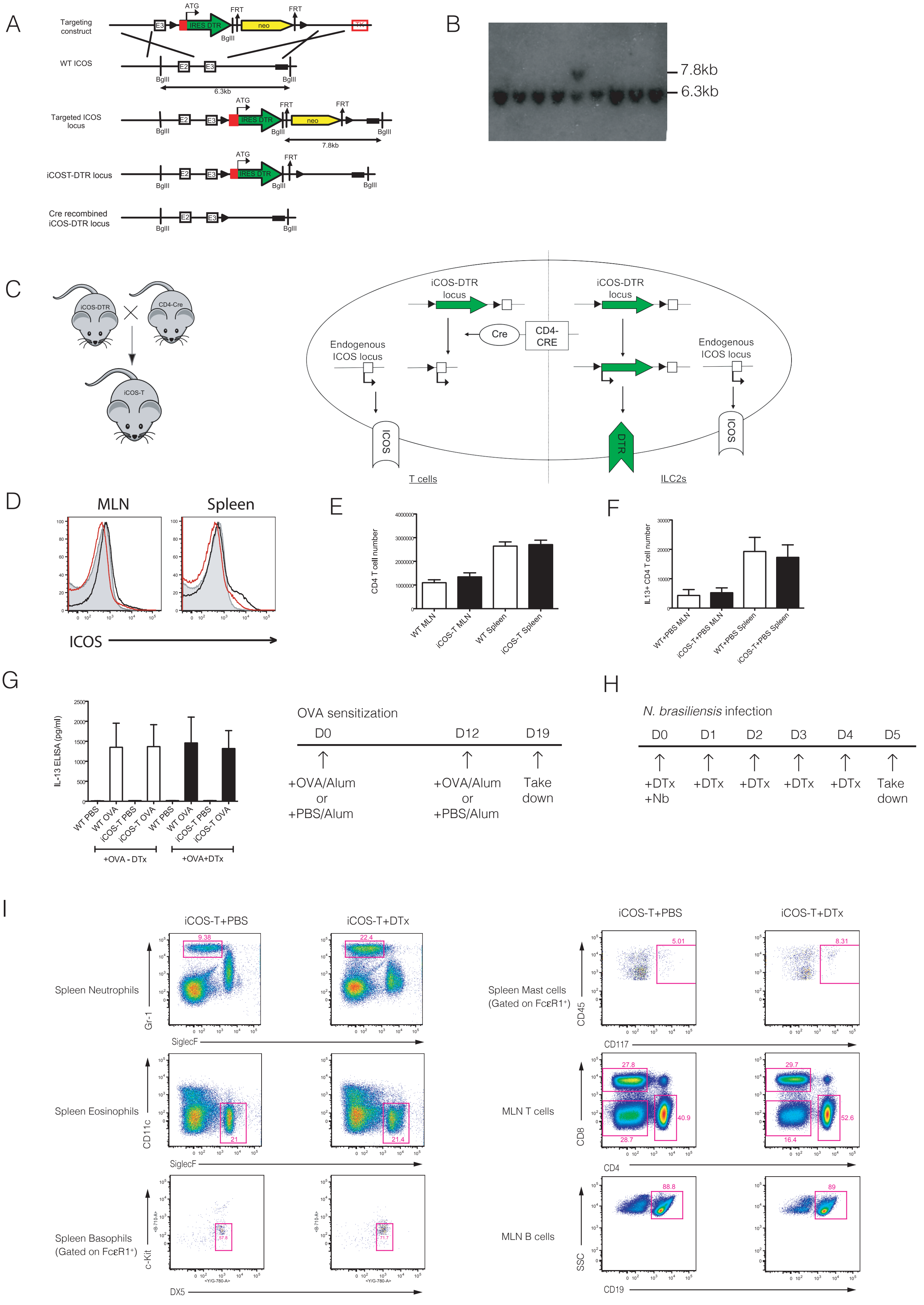

A
Targeting construct
WT ICOS
Targeted ICOS locus
iCOST-DTR locus
Cre recombined iCOS-DTR locus
ATG
FRT
IRES DTR
neo
BglII
6.3kb
7.8kb
B
7.8kb
6.3kb
C
iCOS-DTR
CD4-Cre
iCOS-T
iCOS-DTR locus
Cre
CD4-CRE
Endogenous ICOS locus
ICOS
DTR
T cells
ILC2s
D
MLN
Spleen
ICOS
E
CD4 T cell number
WT MLN
iCOS-T MLN
WT Spleen
iCOS-T Spleen
F
IL13+ CD4 T cell number
WT+PBS MLN
iCOS-T+PBS MLN
WT+PBS Spleen
iCOS-T+PBS Spleen
G
IL-13 ELISA (pg/ml)
WT PBS
WT OVA
iCOS-T PBS
iCOS-T OVA
+OVA - DTx
+OVA+DTx
OVA sensitization
D0
D12
D19
+OVA/Alum or +PBS/Alum
Take down
H
N. brasiliensis infection
D0
D1
D2
D3
D4
D5
+DTx +Nb
+DTx
Take down
I
iCOS-T+PBS
iCOS-T+DTx
Spleen Neutrophils
Gr-1
SiglecF
Spleen Eosinophils
CD11c
Spleen Basophils (Gated on FcεR1+)
c-Kit
DX5
Spleen Mast cells (Gated on FcεR1+)
CD45
CD117
MLN T cells
CD8
CD4
MLN B cells
SSC
CD19

Figure S2

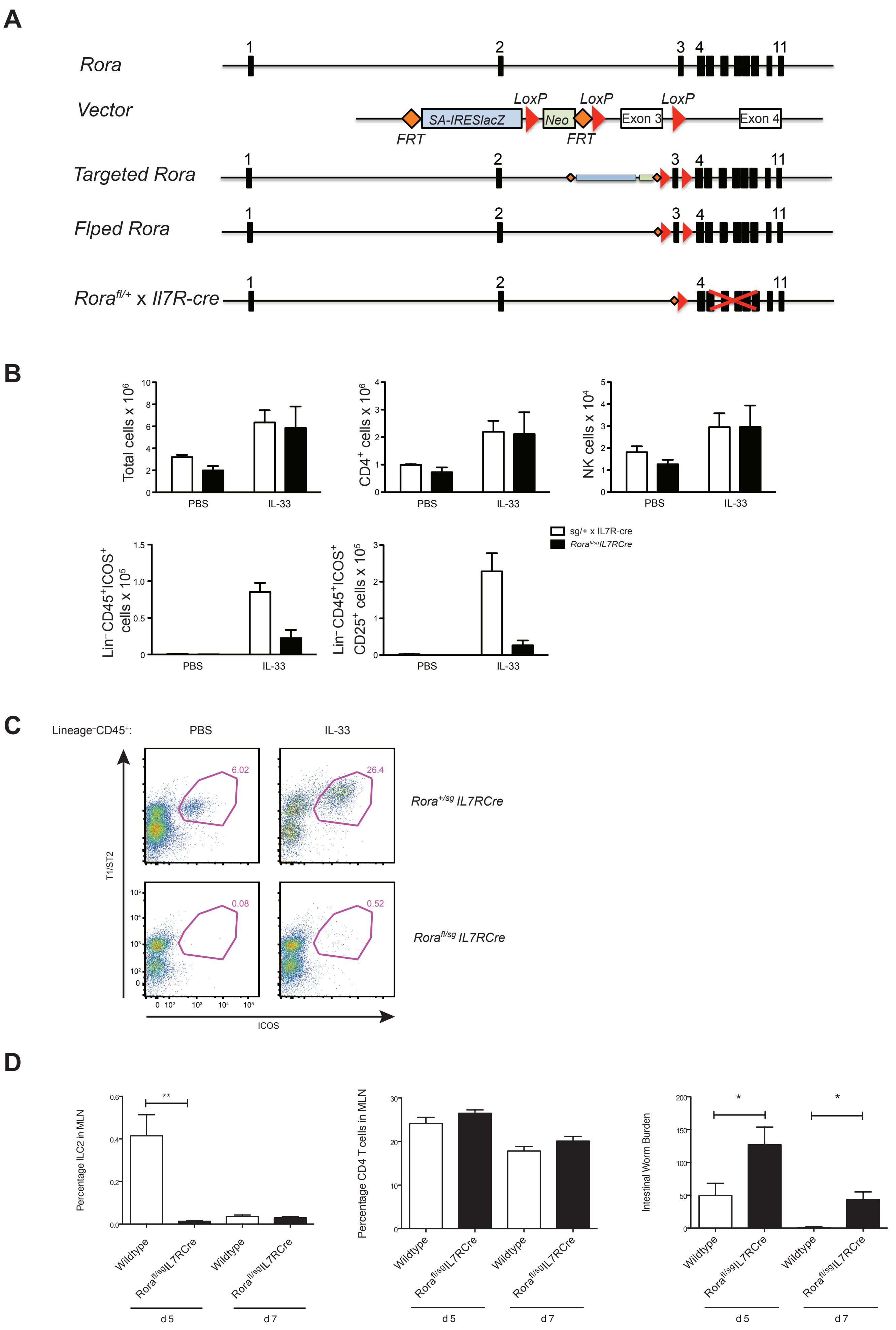

A
Rora
Vector
LoxP
SA-IRESlacZ
Neo
FRT
Exon 3
Exon 4
Targeted Rora
Flped Rora
Rorafl/+ x Il7R-cre
B
Total cells x 10^6
CD4+ cells x 10^6
NK cells x 10^4
PBS
IL-33
Lin−CD45+ICOS+ cells x 10^5
Lin−CD45+ICOS+ CD25+ cells x 10^5
sg/+ x IL7R-cre
Rorafl/sg IL7RCre
C
Lineage−CD45+:
PBS
IL-33
6.02
26.4
0.08
0.52
Rora+/sg IL7RCre
Rorafl/sg IL7RCre
T1/ST2
ICOS
D
Percentage ILC2 in MLN
Percentage CD4 T cells in MLN
Intestinal Worm Burden
Wildtype
Rorafl/sg IL7RCre
d 5
d 7

Figure S3

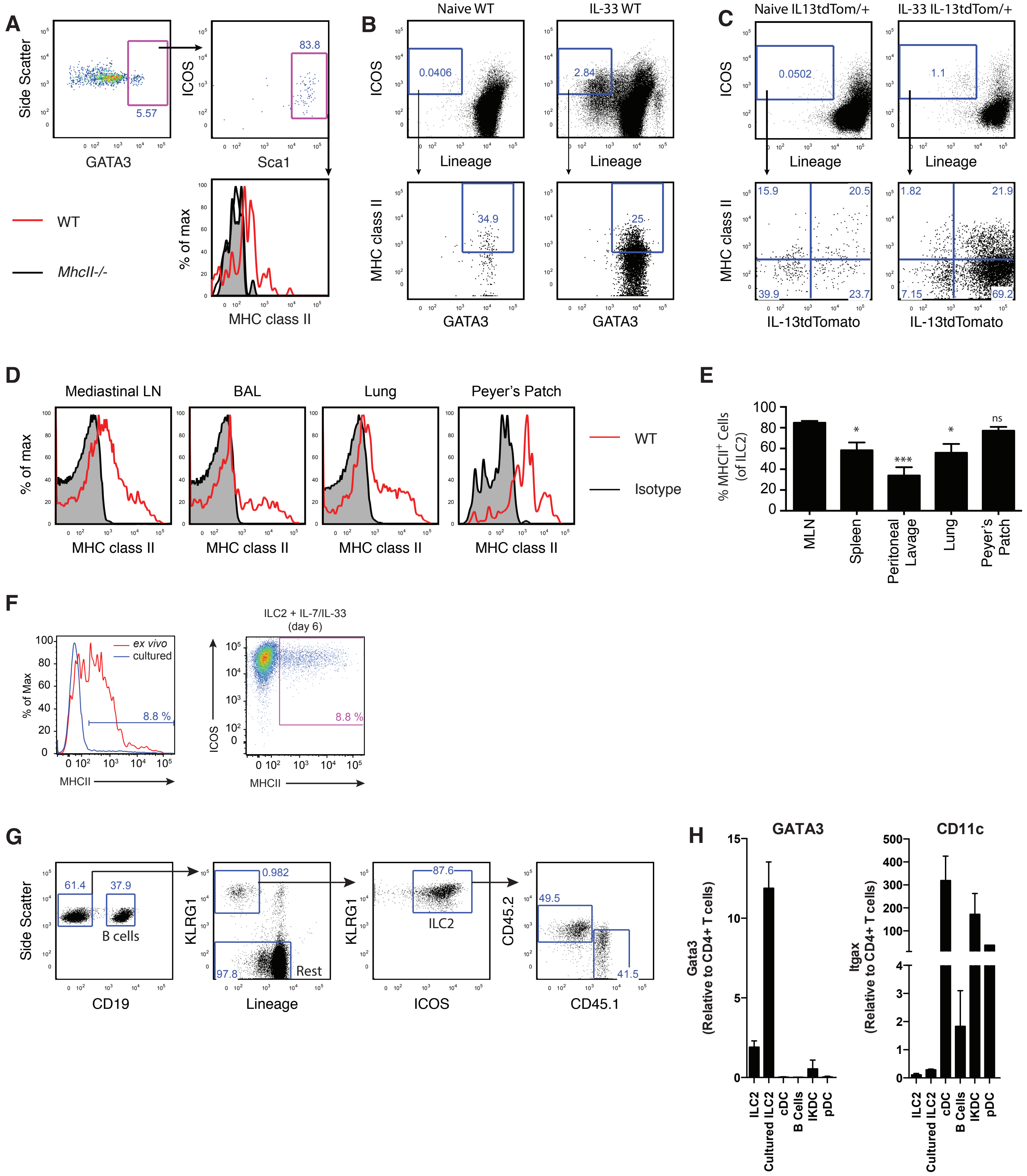

A
Side Scatter
GATA3
5.57
ICOS
Sca1
83.8
% of max
MHC class II
WT
MhcII-/-
B
Naive WT
IL-33 WT
ICOS
Lineage
0.0406
2.84
MHC class II
GATA3
34.9
25
C
Naive IL13tdTom/+
IL-33 IL-13tdTom/+
ICOS
Lineage
0.0502
1.1
MHC class II
IL-13tdTomato
15.9
20.5
39.9
23.7
1.82
21.9
7.15
69.2
D
Mediastinal LN
BAL
Lung
Peyer's Patch
% of max
MHC class II
WT
Isotype
E
% MHCII+ Cells (of ILC2)
MLN
Spleen
Peritoneal Lavage
Lung
Peyer's Patch
ns
F
% of Max
MHCII
ex vivo
cultured
8.8 %
ILC2 + IL-7/IL-33 (day 6)
ICOS
G
Side Scatter
CD19
61.4
37.9
B cells
KLRG1
Lineage
0.982
97.8
Rest
ICOS
87.6
ILC2
CD45.2
CD45.1
49.5
41.5
H
GATA3
Gata3 (Relative to CD4+ T cells)
CD11c
Itgax (Relative to CD4+ T cells)
ILC2
Cultured ILC2
cDC
B Cells
lKDC
pDC

Figure S4

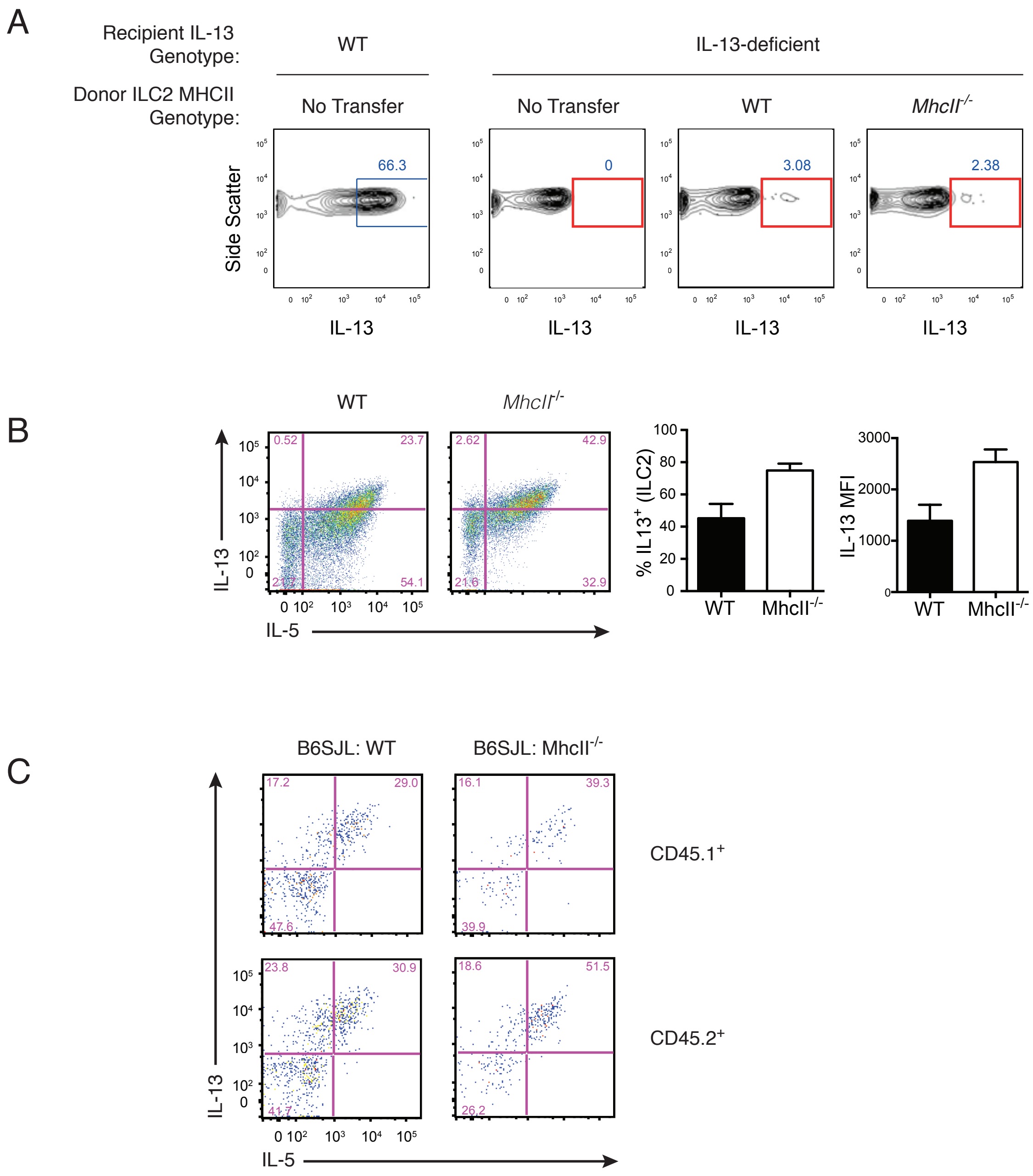
A
Recipient IL-13 Genotype:
WT
IL-13-deficient
Donor ILC2 MHCII Genotype:
No Transfer
No Transfer
WT
Mhcll-/-
Side Scatter
66.3
0
3.08
2.38
IL-13
IL-13
IL-13
IL-13
B
WT
Mhcll-/-
0.52
23.7
54.1
2.62
42.9
21.6
32.9
IL-13
IL-5
% IL13+ (ILC2)
WT
MhcII-/-
IL-13 MFI
WT
MhcII-/-
C
B6SJL: WT
B6SJL: MhcII-/-
17.2
29.0
47.6
16.1
39.3
39.9
23.8
30.9
18.6
51.5
26.2
CD45.1+
CD45.2+
IL-13
IL-5

Figure S5

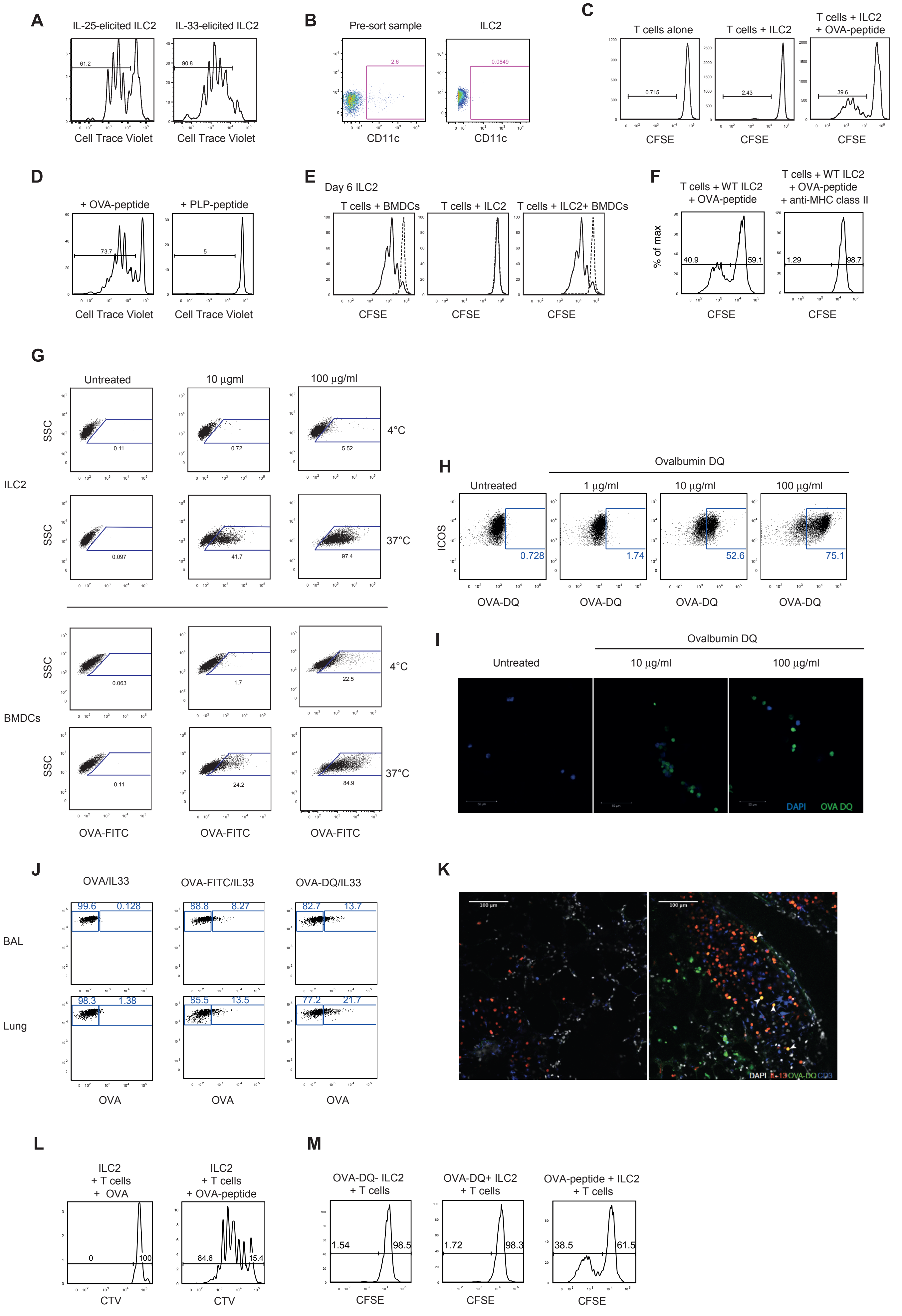
A
IL-25-elicited ILC2
IL-33-elicited ILC2
61.2
90.8
Cell Trace Violet
Cell Trace Violet
B
Pre-sort sample
ILC2
2.6
0.0849
CD11c
CD11c
C
T cells alone
T cells + ILC2
T cells + ILC2 + OVA-peptide
0.715
2.43
39.6
CFSE
CFSE
CFSE
D
+ OVA-peptide
+ PLP-peptide
73.7
5
Cell Trace Violet
Cell Trace Violet
E
Day 6 ILC2
T cells + BMDCs
T cells + ILC2
T cells + ILC2+ BMDCs
CFSE
CFSE
CFSE
F
T cells + WT ILC2 + OVA-peptide
T cells + WT ILC2 + OVA-peptide + anti-MHC class II
% of max
40.9
59.1
1.29
98.7
CFSE
CFSE
G
Untreated
10 µgml
100 µg/ml
ILC2
SSC
0.11
0.72
5.52
4°C
0.097
41.7
97.4
37°C
BMDCs
0.063
1.7
22.5
4°C
0.11
24.2
84.9
37°C
OVA-FITC
OVA-FITC
OVA-FITC
H
Ovalbumin DQ
Untreated
1 µg/ml
10 µg/ml
100 µg/ml
ICOS
0.728
1.74
52.6
75.1
OVA-DQ
OVA-DQ
OVA-DQ
OVA-DQ
I
Ovalbumin DQ
Untreated
10 µg/ml
100 µg/ml
DAPI
OVA DQ
J
OVA/IL33
OVA-FITC/IL33
OVA-DQ/IL33
BAL
99.6
0.128
88.8
8.27
82.7
13.7
Lung
98.3
1.38
85.5
13.5
77.2
21.7
OVA
OVA
OVA
K
100 µm
100 µm
DAPI
OVA-DQ
CD3
L
ILC2 + T cells + OVA
ILC2 + T cells + OVA-peptide
0
100
84.6
15.4
CTV
CTV
M
OVA-DQ- ILC2 + T cells
OVA-DQ+ ILC2 + T cells
OVA-peptide + ILC2 + T cells
1.54
98.5
1.72
98.3
38.5
61.5
CFSE
CFSE
CFSE

Figure S6

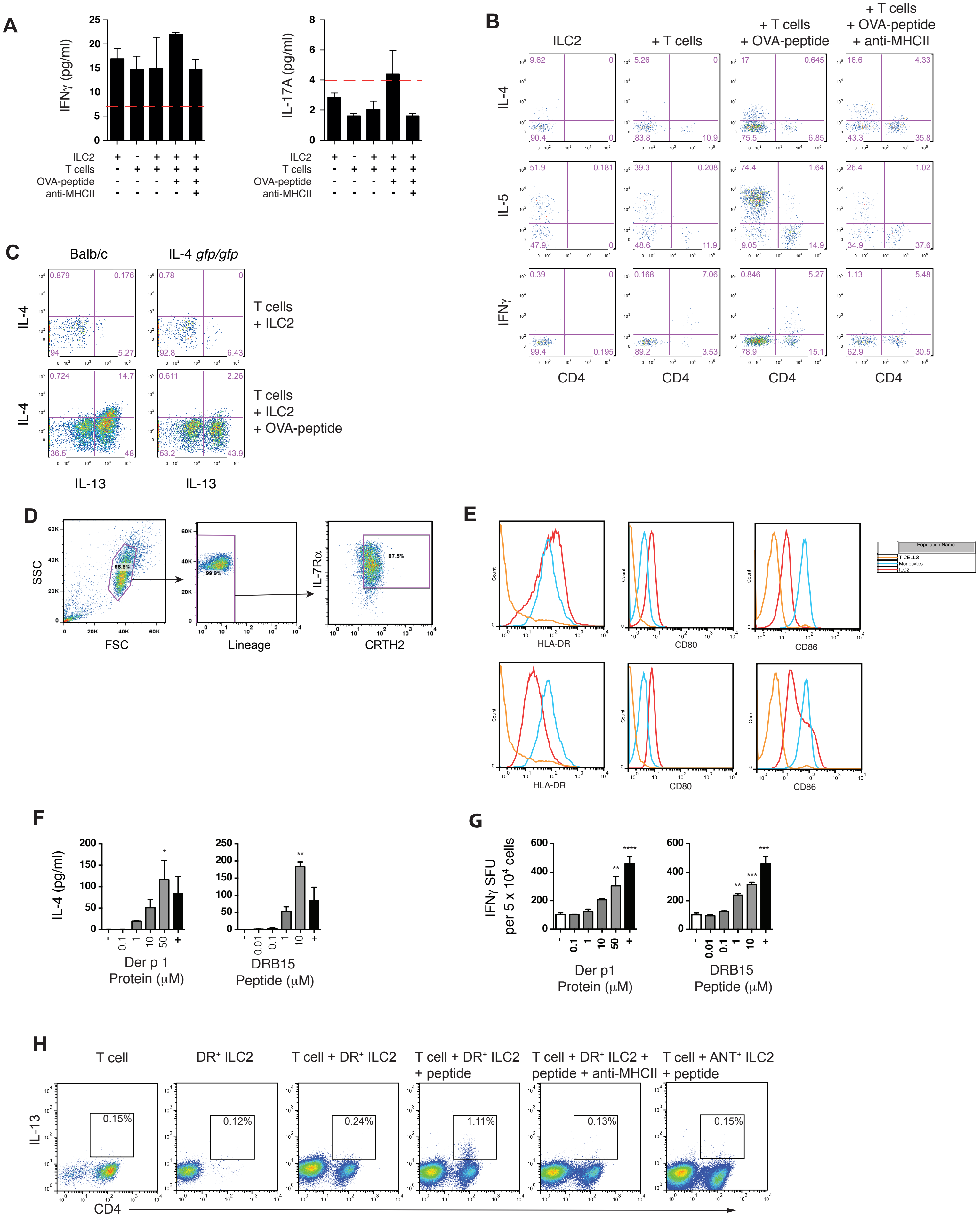
A
IFNγ (pg/ml)
IL-17A (pg/ml)
ILC2
T cells
OVA-peptide
anti-MHCII
B
ILC2
+ T cells
+ T cells + OVA-peptide
+ T cells + OVA-peptide + anti-MHCII
IL-4
IL-5
IFNγ
CD4
C
Balb/c
IL-4 gfp/gfp
T cells + ILC2
T cells + ILC2 + OVA-peptide
IL-13
D
SSC
FSC
Lineage
IL-7Rα
CRTH2
E
HLA-DR
CD80
CD86
Population Name
T CELLS
Monocytes
ILC2
F
IL-4 (pg/ml)
Der p 1 Protein (μM)
DRB15 Peptide (μM)
G
IFNγ SFU per 5 x 10⁴ cells
Der p1 Protein (μM)
DRB15 Peptide (μM)
H
T cell
DR⁺ ILC2
T cell + DR⁺ ILC2
T cell + DR⁺ ILC2 + peptide
T cell + DR⁺ ILC2 + peptide + anti-MHCII
T cell + ANT⁺ ILC2 + peptide
IL-13
CD4

SUPPLEMENTAL FIGURE LEGENDS

Figure S1, related to Figure 1. Generation and analysis of iCOS-T mice

(A) Targeting strategy to insert the IRES-DTR fusion sequence directly after *Icos* exon 3.

(B) Southern blot screens for ICOS-DTR. A positive clone of iCOS-DTR digested with BglII. Transgenic fragment 7.8 kb, Wildtype (WT) fragment 6.3 kb.

(C) iCOS-DTR mice were crossed with CD4-Cre mice to generate iCOS-T mice. Schematic shows how the DTR gene is deleted from T cells and retained in ILC2.

(D) ICOS expression on $CD4^+$ T cells after 3 daily doses of IL-33 at 0.5 μg/dose. Shaded histogram indicates isotype control. Black histogram represents WT ICOS expression. Red histogram represents iCOS-T ICOS expression. n = 5.

(E) Total $CD4^+$ T cell numbers in WT and iCOS-T mice after 3 daily doses of IL-33 at 0.5 μg/dose. n = 3 mice (representative of two independent experiments).

(F) WT and iCOS-T mice were infected with *N. brasiliensis*. 5 days post infection (d.p.i.), MLN and spleen cells were taken and $IL\text{-}13^+CD4^+$ T cell number was determined by intracellular staining. n = 5 mice.

(G) WT and iCOS-T mice were sensitized intraperitoneally with either PBS/Alum or OVA/Alum (20 μg/dose) on day 0 and day 12. On day 19, splenocytes were harvested and plated in duplicate at 2.0×10^5 cells per well. Splenocytes were re-stimulated with OVA (100 μg/ml) *in vitro* in the presence or absence of DTx (100 ng/ml). Cultures were maintained for 3 days at 37°C. Supernatants were analysed for IL-13 by ELISA.

(H) Scheme for the administration of DTx (or PBS) to *N.brasiliensis*-infected iCOS-T mice.

(I) Flow cytometric analysis of myeloid and lymphoid cells from mice treated as depicted in (H) (5 d.p.i.).

Open bars represent wells treated with OVA and untreated with DTx. Black bars represent wells treated with both OVA and DTx. n = 5 mice. * $P < 0.05$. Bar graphs represent mean +/- s.e.m (E-G).

Figure S2, related to Figure 1. Generation and analysis of *Rora*$^{sg/fl}$*IL7RCre mice*

(A) *Rora*-targeted (*Rora*$^{tm1a(EUCOMM)Wtsi}$) embryonic stem cells were obtained from EUCOMM and used to produce *Rora*$^{fl/sg}$*IL7RCre*.

(B) Numbers of cells in the mesenteric lymph nodes of *Rora*$^{fl/sg}$*IL7RCre* mice following IL-33 administration.

(C) Detection of ILC2 precursors in the bone marrow of *Rora*$^{fl/sg}$*IL7RCre* mice following administration of PBS or IL-33.

(D) ILC2, $CD4^+$ T cell and worm burdens in *Rora*$^{fl/sg}$*IL7RCre* mice following *N. brasiliensis* infection. Data are representative of a single experiment with 5 mice per group. Bar graphs represent mean +/- s.e.m (B and D).

Figure S3, related to Figure 2. ILC2 express MHCII

(A) MHCII expression by naïve wildtype or *MhcII*-/- $Lin^-GATA3^+ICOS^+Sca1^{high}$ MLN ILC2 from untreated mice. Plots have been gated on lineage-negative cells.

(B-C) MHCII expression by $GATA3^+$ (B) and IL-13^+ (C) MLN ILC2 (Lin^-ICOS^+) from naïve or IL-33-treated wildtype (B) or IL-13-tdTomato reporter (C) mice.

(D) MHCII expression by wildtype ILC2 from the indicated tissues from IL-33-treated mice.

(E) Proportion of MHCII-positive ILC2 in the indicated tissues of naïve wildtype mice.

(F) MHCII expression *ex vivo* ILC2 or following culture for 6 days with IL-7 and IL-33.

(G) Gating strategy for MLN B cells and ILC2 from mixed chimeras reconstituted with 1:1 ratios of B6SJL and either C57Bl/6 or *MhcII*-/- bone marrow.

(H) Gene expression analysis for *Gata3* and *Itgax* from flow cytometrically sorted populations. IL-33 elicited ILC2 and B cells were from MLN. DC populations were from spleen. * $P < 0.05$, and *** $P < 0.001$; ns, not significant. Data are representative of 2 - 3 experiments with 2 - 5 mice per group (A-E). Bars represent mean +/- s.e.m.

Figure S4, related to Figure 3. Detection of transferred ILC2 in IL-13-deficient recipients

(A) Intracellular IL-13 staining of the ILC2 population (prior gating strategy Lin-CD4-$Klrg1^+ICOS^+$) in the MLN of *N. brasiliensis*-infected IL-13-deficient recipients. Transferred wildtype or *MhcII*-/- ILC2 are highlighted in red boxes. A *N. brasiliensis*-infected wildtype MLN not subjected to cell transfer was included as a positive control for IL-13 staining. Numbers in the outlined areas indicate the

percentage of IL-13$^+$ cells in each gate. Data are representative of four independent experiments with 5 - 6 mice per group.

(B) Intracellular IL-13 and IL-5 staining of ILC2 from IL-33-treated wildtype and MHCII-deficient mice. Bar graphs indicate the percentage of mesenteric lymph node ILC2 that express IL-13 and the mean fluorescence intensity (MFI) of IL-13 staining. Data are representative of 5 mice per group. Bars represent mean +/- s.e.m.

(C) Intracellular IL-13 and IL-5 staining of MLN ILC2 from chimeric mice, generated with a 1:1 ratio of B6SJL (CD45.1$^+$) bone marrow and either C57Bl/6 or *MhcII*-/- bone marrow (CD45.2$^+$). Mice were treated with IL-33.

Figure S5, related to Figure 4. ILC2 induce antigen-dependent T cell activation *in vitro*, and can process endocytosed OVA both *in vitro* and *in vivo*

(A) T cell stimulation with either IL-25 or IL-33 elicited ILC2.

(B) Purity of sorted ILC2 population (dot plot shows 10,000 events).

(C) Proliferation of CFSE-labelled DO11.10 CD4$^+$ T cells, induced by OVA-peptide pre-pulsed ILC2.

(D) Proliferation of CTV-labelled DO11.10 CD4$^+$ T cells in response to OVA-peptide (323-339) or an irrelevant peptide derived from a myelin proteolipid protein.

(E) Proliferation of CFSE-labelled DO11.10 CD4$^+$ T cells, induced by bone marrow-derived DCs (BMDCs) or ILC2, as indicated.

(F) OTIITg CD4$^+$ T cell proliferation as determined by Cell Trace Violet dilution for data in Figure 4E.

(G) *In vitro* endocytosis of fluorescently-labelled ovalbumin (OVA-FITC) by ILC2.

(H-I) Flow cytometry analysis (H) and micrograph (I) of fluorescence resulting from ovalbumin-DQ (OVA-DQ) degradation by ILC2.

(J) Identification of bronchoalveolar lavage and lung ILC2 containing degraded OVA-DQ following co-administration with IL-33 (intranasally). Plots are gated on Lin$^-$ICOS$^+$ cells.

(K) Immunofluorescence detection of OVA-DQ$^+$IL-13$^+$CD3$^-$ ILC2 (arrows) in the lung of IL-13-*tdTomato* reporter mice following intranasal administration of IL-33 and OVA-DQ.

(L-M) OTIITg CD4$^+$ T cell proliferation in the presence of OVA antigen-pulsed ILC2 (L) or FACs sorted OVA-DQ-loaded ILC2 (M).

Data are representative of two independent experiments. MLN ILC2 were elicited using IL-33 (B,D, F and L) or IL-25 (C, E, G, H and I).

Figure S6, related to Figure 5. MHCII-dependent ILC2/CD4$^+$ T cell interactions lead to type-2 cytokine expression

(A) IFNγ and IL-17A concentrations in supernatants following ILC2/OTIITg CD4$^+$ T cell co-cultures, as indicated. Bars represent mean +/- s.e.m and dotted line indicates the limit of detection.

(B) Flow cytometry analysis of intracellular cytokine staining of ILC2 and CD4$^+$ OTIITg T cells following co-cultures, as indicated.

(C) Intracellular IL-4 and IL-13 staining of wildtype, or IL-4-deficient (IL-4$^{gfp/gfp}$), ILC2 in antigen-dependent co-cultures, as indicated.

Data are representative of 2 - 3 independent experiments.

(D) Sort purity of human ILC2 (Lin$^-$IL7Ra$^+$CRTH2$^+$) used for *in vitro* co cultures.

(E) Comparison of HLA-DR, CD80, and CD86 expression on T cells, monocytes and ILC2 from two separate donors.

(F) IL-4 concentration in the supernatants following human ILC2:T cell co-culture in the presence of either Der p 1 or DRB15, or PMA and ionomycin (+).

(G) IFNγ expression in culture supernatants following ILC2:T cell co-cultures in the presence of Der p 1 protein, or peptide (DRB15), or PMA and ionomycin (+).

(H) Intracellular IL-13 staining of human CD4$^+$ T cells stimulated in the presence of Der p 1 peptide DRB15 with MHCII-matched DR$^+$ ILC2 or MHCII-mismatched ANT$^+$ ILC2. Data are from a single experiment.

Bar graphs represent mean +/- s.e.m. * $P < 0.05$, ** $P < 0.01$, *** $P < 0.001$ and **** $P < 0.0001$. Data are representative of 2 - 3 independent experiments (D-G) or a single experiment (H).

SUPPLEMENTAL EXPERIMENTAL PROCEDURES

Generation of iCOS-DTR and iCOS-T mice

The floxed IRES2-DTR gene cassette was generated in house. The IRES2 sequence was purchased from Clontech (pTRE3G-IRES Vector Set: Cat no. 631174). The IRES2 sequence was amplified by PCR to generate a product with an XhoI restriction enzyme site and a loxP site at the 5' end and a BclI restriction enzyme site at the 3' end. The diphtheria toxin receptor gene cassette created by See Heng Wong was amplified by PCR to generate a product with an XhoI restriction site at the 5' end, a BamHI restriction enzyme site at the ATG start codon and a loxP site at the 3' end. Both PCR products were sequentially cloned into the TOPO4 vector backbone to generate a floxed IRES2-DTR gene cassette that fused the start codon of the DTR gene with the internal start codon of the IRES2 sequence.

The iCOS-DTR mice were then generated by recombineering[1]. The floxed IRES2-DTR gene cassette had 50 base pair (bp) arms of homology for recombineering added to either ends by PCR primers (forward, 5'-TTGTGGTACTCCTTTTTGGATGCATACTTATCATCTGGTTTTCAAAAAGC GAATTACTCGAGATAACTTC-3', reverse, 5'-AAAAAAACACCTAGGAAAGAAAGTATAAGAAAAAAATAAAAATCACTTA CTCTAGAACTAGTGGTTCCACC-3')

The floxed IRES2-DTR gene cassette was inserted immediately downstream of *Icos* exon 3 in the center of a 6kb length of genomic DNA. The *Icos* exon 3 genomic DNA was derived from the BAC bMQ310-P20 (Source Bioscience) by PCR (forward, 5'-AAATTTGCGGCCGCTTCTAACTCCTCCATTAGGGACCC-3', reverse, 5'-CAGGGGAACTAGTCCATGCGTTTC-3').

The iCOS-DTR construct was transfected into CCB embryonic stem cells and the clones were screened by Southern Blot analysis using *Bgl*II. The 3' screening probe was obtained by PCR of genomic DNA (5'- GGACCACAGGGCACCTGACTTG-3', 5'-GGCTGAGCTTCCTATTTGGAG-3'), yielding a probe of 616 bp. The expected wildtype band is 6.3 kb, while the expected target band is 7.8 kb. Targeted CCB

embryonic stem cell clones were used to generate an iCOS-DTR mouse line on a mixed background. The neomycin resistance gene was removed by breeding iCOS-DTR mice with FLP recombinase mice. Neomycin and FLP recombinase negative mice were then backcrossed 4 times to the C57Bl/6 background and subsequently crossed with CD4-Cre mice (Taconic, Nodel no. 4196) to generate iCOS-T mice. T cells in iCOS-T mice excise the DTR gene while ILC2 retain it. Genotyping of iCOS-T mice used PCR primers (5'-GACTCAGTGGCTATTCCGTCTACTTG-3', 5'-GGAAAATGCATGGTCTATCCTAGC-3', 5'-GGGAGAGGGGCATAACTTCGTATAGC-3'), giving a wildtype product of 562 bp and a targeted product of 452 bp. A separate Cre PCR was also used for genotyping (5'-TACCTGGCCTGGTCTGGACACAGTG-3', 5'-ATGGCTAATCGCCATCTTCCAGCAG-3').

Generation of *Rora*$^{+/\mathbf{flox}}$*IL7RCre* mice

Rora-targeted (*Rora*$^{tm1a(EUCOMM)Wtsi}$) embryonic stem cells were obtained from EUCOMM and used to generate *Rora*-targeted mice from which the neomycin selection and βgal cassettes were removed by inter-crossing with Flp-recombinase mice to give *Rora*$^{fl/+}$ mice. *Rora*$^{fl/+}$ mice were crossed with *Rora*$^{sg/+}$*IL7RCre* mice to produce *Rora*$^{sg/fl}$*IL7RCre* and *Rora*$^{sg/+}$*IL7RCre* mice. Genotyping of *Rora*$^{fl/+}$ mice was undertaken using PCR primers (5'-TGAGTGGTAACACCACGGCACGC-3', 5'-TGGAGCAGAATCATCCAGGAGGCC-3', 5'-CAACGGGTTCTTCTGTTAGTCC-3'), giving wildtype product of 573 bp and a targeted product of ~650 bp.

Fluorescence-activated cell analysis and cell sorting

Mouse tissue cell suspensions were incubated with purified anti-Fc receptor blocking antibody (anti-CD16/CD32) before addition of the specific antibodies. Cell surface markers were stained using a combination of fluorescein isothiocyanate (FITC)-, phycoerythrin (PE)-, PE-Cy7-, PerCP-Cy5.5-conjugated, allophycocyanin-conjugated, Alexa Fluor 647–conjugated, eFluor® 660-conjugated, eFluor 450–conjugated, Brilliant Violet 421-conjugated, Brilliant Violet 510-conjugated, APC-Cy7-conjugated and biotin-conjugated monoclonal antibodies. For intracellular cytokine staining a Fix/Perm kit was used and was completed according to the manufacturer's protocols (BD Bioscience). Intracellular staining with anti-GATA3

(eBioscience) was stained following the FoxP3 Staining Buffer Set (eBioscience) and the manufacturer's instructions. Annexin V staining was performed according to the manufacturer's instructions with an Annexin V Apoptosis Detection Kit (eBioscience). In each experiment the appropriate isotype control monoclonal antibodies and single conjugate controls were also included. Samples were analysed using a Becton Dickinson LSRII or LSRFortessa™ flow cytometer running FACSDiva™ acquisition and analysed using FlowJo software (version 8.8.3, Tree Star). Cells were sorted using a Sony iCyt Synergy cell sorter.

Antibodies for flow cytometry analysis: primary antibodies anti-CD4 (GK1.5); anti-CD8a (53-6.7); anti-CD19 (1D3); anti-B220 (RA3-6B2); anti-Gr-1 (RB6-8C5); anti-CD11c (N418); anti-TER119 (TER-119); anti-CD25 (PC61.5); anti-CD44 (1M7); anti-CD45.1 (A20); anti-CD45.2 (104); anti-CD69 (H1.2F3); anti-NK1.1 (PK136); anti-TCRγδ (eBioGL3); anti-KLRG1 (2F1); anti-IL-4 (11B11); anti-IL-5 (TRFK5); anti-IL-13 (eBio13A); anti-MHCII (I-A/I-E) (M5/114.15.2); fixable viability dye eFluor-780 and unconjugated anti-CD16/32 (93) were purchased from eBioscience, UK. Streptavidin conjugated to PE, PE-Cy7 or AF647 were purchased from eBioscience. Anti-ICOS (C398.4A); anti-CD3e (145-2C11) ; anti-CD11b (M1/70) ; anti-IL-7Rα (SB/199) ; anti-FcεRIα (MAR-1) ; anti-CD80 (16-10A1) and anti-CD86 (GL-1) were purchased from Biolegend. Anti-CD5 (53-7.3) ; anti-IL-2 (JES6-5H4) ; anti-CD62L (MEL-14) and anti-TCRβ (H57-597) were purchased from BD Bioscience. Anti-T1/ST2 (DJ8) conjugated to biotin was purchased from MD Biosciences. All isotype control antibodies, rat anti-mouse IgG1, IgG2a, IgG2b and Armenian hamster anti-mouse IgG were purchased from eBioscience.

Enzyme-linked Immunosorbent Assay

Murine IL-4 and IL-5 ELISA were performed using purified anti-IL-4 (eBioscience) and anti-IL-5 (BD Biosciences). Briefly, culture supernatants were added to antibody coated Nunc-Immuno plates (Thermo Scientific) for 12 hours. Cytokine concentration was detected by sequential incubation with a biotinylated detection anti-cytokine antibody (IL-4 (BD Bioscience), and IL-5 (BD Bioscience), streptavidin-horseradish peroxidase and an HRP colorimetric reagent. Murine IL-13 was detected with Quantikine IL-13 (R&D Systems) or mouse IL-13 ELISA Ready-

Set-Go! (eBioscience). Human IL-4 ELISA (eBioscience) were performed according to the manufacturer's protocol. Supernatant was added to anti-IL-4 coated Nunc-Immuno plate (Thermo Scientific) for 24 hours. Cytokine assays were also performed using the MagPix® system according to standard protocols.

CFSE and Cell trace Violet Staining

Cells were stained with Cell Trace™ CFSE or Cell Trace™ Violet Cell Proliferation Kits (Invitrogen) according to the manufacturer's instructions at a final concentration of 5 μM.

Bone Marrow-Derived DC (BMDC) Cultures

BMDCs were generated as previously described from the femurs and tibias of wildtype mice (Lutz et al., 1999) using 20 ng/ml recombinant mouse GM-CSF (R & D Systems).

In vitro OVA-FITC and OVA-DQ Uptake Assays

For the *in vitro* OVA-FITC uptake assays BMDC or IL-7/IL-33 cultured ILC2 were incubated for 4 hours with culture medium alone or in the presence of FITC-conjugated OVA (10 μg/ml or 100 μg /ml) at either 4°C or 37°C. OVA-DQ endocytosis and degradation assays were performed by incubating DQ-conjugated OVA (1-100 μg/ml) with freshly isolated ILC2 for 16 hours at 37°C, 5% CO_2. Cytospins of OVA-DQ treated ILC2 were performed by centrifuging samples in a Shandon Cytospin 3 (Shandon) at 900 rpm for 10 minutes and subsequently analysed for fluorescence on a Carl-Zeiss inverted microscope (LSM 710) and processed with ZEN 2008 (Carl-Zeiss).

Immunofluorescence of OVA-DQ Uptake Assays

For immunofluorescence micrographs, $IL13^{+/tdTomato}$ mice were treated with OVA-DQ and IL-33 i.n, and lungs were then prepared as described previously (Scanlon et al., 2011). After blocking, sections were incubated with purified Armenian hamster anti-mouse CD3ε (Clone 145-2C11, 1.25 μg/ml) (BioLegend) for 1 h, sections were incubated with Alexa Fluor 647-conjugated goat anti-Armenian hamster antibody (3 μg/ml) (Jackson Immunoresearch) and 300 nM DAPI (Biotium) for 30 min. Sections

were then mounted with ProLong Gold antifade reagent (Invitrogen) according to manufacturer's instructions. Sections were imaged with an LSM 710 confocal scanner (Zeiss) mounted on an Axio Observer.Z1 microscope (Zeiss) equipped with an EC Plan-NEOFLUAR 20X/0.5 objective (Zeiss). Data were recorded using Zen imaging software (Zeiss) and then analysed using ImageJ software (National Institutes of Health).

Quantitative Reverse Transcriptase PCR

APC populations and ILC2 were purified (by fluorescence-activated cell sorting) from the mesenteric lymph nodes of naïve mice or those challenged with three daily intraperitoneal injections of 0.5 μg recombinant mouse IL-33, respectively. RNA was purified and reverse-transcribed. Taqman primer and probe sets (Applied Biosystems) were used for quantification of the expression of *H2-Aa* (Mm00439211_m1), *H2-Ab1* (Mm00439216_m1), *H2-Eb1* (Mm00439221_m1), *Ciita* (Mm00482914_m1), *CD74* (Mm00658576_m1), *Itgax* (Mm00498698_m1) and *Gata3* (Mm00484683_m1). Expression was quantified relative to that of *Gapdh*.

Human ILC2 and T cell purification and stimulation

The ILC2 population and $CD4^+$ T cell lines were generated from the peripheral blood of HLA-DRB1*15-positive donors. For the isolation of ILC2, PBMC were fluorescently labelled using the lineage markers; CD3 (SK7; BD Biosciences), CD19 (SJ25C1; BD Biosciences), CD123 (FAB301C; R&D systems), CD11b (DCIS1/18; Biolegend), CD11c (BU15; Abcam), CD8 (RPA-T8; Biolegend), FcεRI (AER-37 (CRA-1); Biolegend), CD14 (MφP9; BD Biosciences), CD4 (MEM-241; Abcam), CD56 (HCD56; Biolegend). The negative population that express CD45 (HI30; Biolegend), CRTH2 (BM16; Miltenyibiotec), IL-7Rα (A019D5; Biolegend) were purity sorted from the lymphoid population on a MoFlo™ XDP cell sorter into 96 well plates and cultured with 100 IU/ml IL-2 (Peprotech 200-02) and Gamma-irradiated peripheral blood mononuclear cells (PBMCs) from 3 healthy volunteers (2 x 10^6 cells/ml). Cultures were stained with HLA-DR (BD Biosciences, G46-6), CD80 (Biolegend, 2D10) and CD86 (BD Biosciences, 2331) antibodies and acquired using Summit software on a CyAn flow Cytometer. FlowJo and Summit software were used for further data analysis.

PBMC were separated from heparinized whole blood on Lymphoprep™ (Axis-Shield PoC AS, Oslo, Norway) washed and resuspended in RPMI 1640 (Gibco®) supplemented with 2 mM L-glutamine, 50 U/ml penicillin, 50 μg/ml streptomycin and 10% Human Serum (R10H). Using the known sequence for Der p 1 (swissprot: locus DERP1_DERPT, accession P08176, recombinant protein Indoor Biotechnologies Ltd, USA), relevant HLA-DRB1*1501 peptide (Ardern-Jones et al., 2007) (AVNIVGYSNAQGVDY), was constructed in house using F-moc chemistry and purity confirmed using HPLC. Peptides were added each at a final concentration of 4 μM. LoTox™ Recombinant Der p 1 deglycosylated (LTR-DP1D-1) was obtained from Indoor Biotechnologies Ltd, USA and was protease-deficient in the absence of DTT (not added). The cells were incubated at 37°C with 5% CO2. At days 3 and 7 IL-2 was added to a final concentration of 100 IU/ml. At day 10 the cells were removed from the plate, washed twice in sterile PBS and returned overnight to unused wells in R10H. At Day 11 the cells were then used in ELISpot analyses as below at 4×10^4/well.

ELISpot analyses

ELISpot analyses were performed as previously described (Lalvani et al., 1997). T cell lines input cell numbers were 4×10^4/well. The plates were incubated overnight at 37°C and 5% CO_2 and were developed with streptavidin-alkaline phosphatase (Mabtech AB) and AP conjugate substrate kit (BioRad).

HLA typing

Donors were tissue typed by PCR-SSP phototyping (Bunce et al., 1995).

SUPPLEMENTAL REFERENCES

Ardern-Jones, M.R., Black, A.P., Bateman, E.A., and Ogg, G.S. (2007). Bacterial superantigen facilitates epithelial presentation of allergen to T helper 2 cells. Proc Natl Acad Sci U S A 104, 5557-5562.

Bunce, M., O'Neill, C.M., Barnardo, M.C., Krausa, P., Browning, M.J., Morris, P.J., and Welsh, K.I. (1995). Phototyping: comprehensive DNA typing for HLA-A, B, C, DRB1, DRB3, DRB4, DRB5 & DQB1 by PCR with 144 primer mixes utilizing sequence-specific primers (PCR-SSP). Tissue Antigens 46, 355-367.

Lalvani, A., Brookes, R., Hambleton, S., Britton, W.J., Hill, A.V., and McMichael, A.J. (1997). Rapid effector function in CD8+ memory T cells. J Exp Med 186, 859-865.

Liu, P., Jenkins, N.A., and Copeland, N.G. (2003). A highly efficient recombineering-based method for generating conditional knockout mutations. Genome Res 13, 476-484.

Lutz, M.B., Kukutsch, N., Ogilvie, A.L., Rossner, S., Koch, F., Romani, N., and Schuler, G. (1999). An advanced culture method for generating large quantities of highly pure dendritic cells from mouse bone marrow. J Immunol Methods 223, 77-92.

Scanlon, S.T., Thomas, S.Y., Ferreira, C.M., Bai, L., Krausz, T., Savage, P.B., and Bendelac, A. (2011). Airborne lipid antigens mobilize resident intravascular NKT cells to induce allergic airway inflammation. J Exp Med 208, 2113-2124.
