# Supplementary material for: MHCII-Mediated Dialog between Group 2 Innate Lymphoid Cells and CD4+ T Cells Potentiates Type 2 Immunity and Promotes Parasitic Helminth Expulsion
Source: Immunity. 2014 Aug 21;41(2):283–95. doi: 10.1016/j.immuni.2014.06.016 (PMC4148706; doi:10.1016/j.immuni.2014.06.016)
Supplement: Document S2. Article plus Supplemental Information [file mmc2.pdf]

CellPress

MHCII-Mediated Dialog between Group 2 Innate Lymphoid Cells and CD4+ T Cells Potentiates Type 2 Immunity and Promotes Parasitic Helminth Expulsion

Christopher J. Oliphant,[1,7] You Yi Hwang,[1,7] Jennifer A. Walker,[1,7] Maryam Salimi,[2] See Heng Wong,[1,8] James M. Brewer,[6] Alexandros Englezakis,[1] Jillian L. Barlow,[1] Emily Hams,[3] Seth T. Scanlon,[1] Graham S. Ogg,[2] Padraic G. Fallon,[3,4,5] and Andrew N.J. McKenzie[1,*]
[1]MRC Laboratory of Molecular Biology, Francis Crick Avenue, Cambridge, CB2 0QH, UK
[2]MRC Human Immunology Unit, NIHR Biomedical Research Centre, University of Oxford, John Radcliffe Hospital, OX3 9DS, UK
[3]Trinity Biomedical Sciences Institute, Trinity College Dublin, Dublin 2, Ireland
[4]National Children's Research Centre, Our Lady's Children's Hospital, Crumlin, Dublin 12, Ireland
[5]Institute of Molecular Medicine, Trinity College Dublin, Dublin 2, Ireland
[6]Institute of Infection, Immunity and Inflammation, GRBC, University Place, Glasgow, G12 8TA, UK
[7]Co-first author
[8]Present address: MedImmune, Milstein Building, Granta Park, Cambridge, CB1 6GH, UK
*Correspondence: anm@mrc-lmb.cam.ac.uk
http://dx.doi.org/10.1016/j.immuni.2014.06.016
This is an open access article under the CC BY license (http://creativecommons.org/licenses/by/3.0/).

SUMMARY

Group 2 innate lymphoid cells (ILC2s) release interleukin-13 (IL-13) during protective immunity to helminth infection and detrimentally during allergy and asthma. Using two mouse models to deplete ILC2s in vivo, we demonstrate that T helper 2 (Th2) cell responses are impaired in the absence of ILC2s. We show that MHCII-expressing ILC2s interact with antigen-specific T cells to instigate a dialog in which IL-2 production from T cells promotes ILC2 proliferation and IL-13 production. Deletion of MHCII renders IL-13-expressing ILC2s incapable of efficiently inducing *Nippostrongylus brasiliensis* expulsion. Thus, during transition to adaptive T cell-mediated immunity, the ILC2 and T cell crosstalk contributes to their mutual maintenance, expansion and cytokine production. This interaction appears to augment dendritic-cell-induced T cell activation and identifies a previously unappreciated pathway in the regulation of type-2 immunity.

INTRODUCTION

Group 2 immunity is believed to have evolved to combat parasitic helminth infection, but also contributes to wound healing. These responses are characterized by adaptive T helper 2 (Th2) cells expressing interleukin-4 (IL-4), IL-5, and IL-13, B cells secreting immunoglobulin E (IgE), eosinophils, and mast cells. Innate lymphoid cells (ILCs) also play key roles in type-2 responses by producing high amounts of type-2 cytokines (Moro et al., 2010; Neill et al., 2010; Price et al., 2010). ILC2s arise rapidly during helminth challenge, preceding the expansion of the adaptive Th2 response. This raises the question of whether ILC2s contribute to the initiation, polarization, or potentiation of adaptive immunity. Unlike type-1 responses, characterized by Th1 cells expressing interferon-γ (IFN-γ), where macrophages and dendritic cells provide the Th1-cell-inducing factor IL-12, the pathways that elicit and potentiate Th2 cells and type-2 immunity are less well defined. Although IL-4 is a key factor in Th2 cell differentiation, type-2 immunity continues in its absence, indicating additional routes of activation (Forbes et al., 2010; Kopf et al., 1993). IL-25 and IL-33 have been reported to induce T cell expression of type-2 cytokines, and it is these two epithelium-derived factors that potently induce ILC2s at the initiation of type-2 immunity (Moro et al., 2010; Neill et al., 2010). A potential role for ILC2s in influencing the adaptive type-2 response was indicated in an early report demonstrating that a non-B/non-T cell population (later called ILC2) was capable of biasing T cells to a more type-2 phenotype (Fallon et al., 2006). Later, the transfer of ILC2s into IL-13-deficient mice, which displayed reduced T cell responses following helminth infection, was shown to restore Th2 cell responses in vivo (Neill et al., 2010). More recently, ILC2-produced IL-13 has been linked to the migration of dendritic cells (DCs) and the support of Th2 cell differentiation (Halim et al., 2014).

Previous studies have demonstrated that MHCII-expressing conventional antigen-presenting DCs play a critical role in generating type-2 responses (Hammad et al., 2010; Phythian-Adams et al., 2010). MHCII is also expressed on B cells, plasmacytoid DCs, and IFN-γ-producing killer DCs (IKDCs), though in lower amounts than on conventional DCs (Chan et al., 2006; Taieb et al., 2006). More controversial reports advocated a role for basophils in antigen presentation (Perrigoue et al., 2009; Sokol et al., 2009; Yoshimoto et al., 2009). However, the generation of basophil null mice failed to show any major defects in primary Th2 cell responses (Ohnmacht et al., 2010). Thus, although basophils express MHCII, the key function of this molecule in their biology remains to be fully elucidated.

MHCII is expressed on ILC2s and ILC3s (Hepworth et al., 2013; Neill et al., 2010). MHCII on ILC3s, in the absence of the

costimulatory molecules CD80 and CD86, resulted in ILC3-mediated suppression of intestinal immune responses against commensal bacteria (Hepworth et al., 2013). However, the role of MHCII on ILC2s remains to be fully characterized. Here we introduce two complementary in vivo models for ILC2 depletion and demonstrate the importance of ILC2s for the efficient development of rapid Th2 cell responses during the expulsion of the parasitic worm *Nippostrongylus brasiliensis*.

RESULTS

ILC2s Are Required for Efficient Development of Th2 Cells during *N. brasiliensis* Infection

We have shown that mice lacking the IL-25 receptor fail to induce ILC2s efficiently during *N. brasiliensis* infection. Concurrently, the impaired Th2 cell responses that were observed in these mice implied a role for ILC2s in promoting adaptive immunity (Neill et al., 2010). To define the roles of ILC2s and Th2 cells during a type-2 immune response, we generated two complementary mouse models to enable ILC2 ablation during helminth infection. First, we generated a mouse strain in which ILC2s can be depleted temporally by the administration of diphtheria toxin (DTx). As inducible T cell costimulator (ICOS) is expressed preferentially on both T cells and ILC2s, we inserted a floxed DTx receptor (DTR) gene into the *Icos* locus (resulting in a null allele) enabling the CD4-cre-mediated excision of the DTR gene from T cells but its retention in ILC2s (see Figures S1A–S1C available online). Thus, the treatment of inducible ICOS-diphtheria toxin receptor (iCOS-T) mice with DTx allowed us to selectively deplete ILC2s while sparing T cells (Figures 1A and 1B).

After *N. brasiliensis* infection and DTx treatment, we observed a higher worm burden at day 5 after infection in ILC2-depleted iCOS-T mice compared to controls (Figure 1C), correlating with the specific depletion of ILC2s (Figure 1D), but maintenance of other cell populations (Figures S1H and S1I). Notably, we also observed that ILC2-depleted iCOS-T mice exhibited a substantial decrease in IL-5- and IL-13-producing CD4$^+$ T cells (Figure 1E). Because this deficit primarily affected ICOS$^+$CD4$^+$ T cells, we explored the possibility that ICOS haploinsufficiency might affect CD4$^+$ T cell function. ICOS expression on CD4$^+$ T cells from iCOS-T mice was reduced as compared to controls (Figure S1D), but T cell-intrinsic defects were not observed following IL-33 administration or *N. brasiliensis* infection (Figures S1E and S1F). The impaired Th2 cell response might be explained through an off-target effect of DTx on ICOS$^+$CD4$^+$ T cells. While restimulation of ovalbumin-primed splenocytes in vitro in the presence of DTx suggested that this was unlikely (Figure S1G), we generated another strain of ILC2-deficient mice and repeated the infection studies.

The transcription factor retinoid-related orphan receptor alpha (RORα) is critical for ILC2 development (Wong et al., 2012). We have established a conditionally targeted *Rora*$^{fl/sg}$ mouse that when intercrossed with IL-7 receptor (IL-7R)-cre (restricted to the lymphoid lineage) mice yields an ILC2-deficient mouse strain in which other lineages are unaffected (Figures S2A–S2C). *Rora*$^{fl/sg}$*Il7r*^{Cre} ILC2-deficient mice were infected with *N. brasiliensis* and cytokine expression was determined after 5 days. Although we observed little difference in worm burden at day 5 compared to controls (Figure 1F), this strain does show delayed worm expulsion (Figure S2D). The *Rora*$^{fl/sg}$*Il7r*^{Cre} ILC2-deficient mice had reduced ILC2s (Figure 1G) and a dramatic deficit in IL-5 and IL-13-producing ICOS$^+$CD4$^+$ T cells (Figure 1H).

Thus, in two distinct ILC2-deficient mouse strains in which ILC2 ablation is accomplished through discrete pathways, we observed concordant in vivo defects in CD4$^+$ Th2 cells. These data support an in vivo interplay between ILC2s and CD4$^+$ T cells, and we proceeded to investigate the potential role of ILC2-expressed MHCII in this interaction.

MHCII Is Expressed, but Also Acquired, by ILC2s

We confirmed MHCII expression on ~50%–70% of naive wild-type (WT) ILC2s (Lin$^-$Klrg1$^+$CD25$^+$ST2$^+$ICOS$^+$Sca1^{hi}GATA3$^+$IL-13$^+$) and those elicited in response to IL-25 or IL-33, as compared to ILC2s from MHCII-deficient mice (*H2-Ab1*$^{-/-}$, referred to throughout as *MhcII*$^{-/-}$) (Figure 2A and 2B; Figures S3A–S3C). Expression of MHCII on ILC2s was at lower amounts than that on B cells (Figure 2C). Lymph node, spleen, and Peyer's patch-derived ILC2s expressed MHCII, but ILC2s from the peritoneal lavage, bronchoalveolar lavage, and lung had considerably lower frequency of MHCII expression (Figure 2C; Figure S3D and S3E). A proportion of ILC2s also expressed the costimulatory molecule CD80 and to a lesser extent CD86 (Figure 2D), which are required, in combination with MHCII, for T cell activation and survival. Thus ILC2s express MHCII on their surface and have the potential to interact directly with CD4$^+$ T cells via this molecule.

ILC2 culture, for as little as 2 days, resulted in loss of cell surface expression of MHCII and by day 6 of culture only ~10% of ILC2s retained MHCII expression (Figure 2E; Figure S3F). Annexin V staining confirmed that this was not due to selective apoptosis of MHCII$^+$ cells (Figure 2F), raising the possibility that the presence of MHCII on ILC2s might arise through trogocytosis from antigen-presenting cells (APCs) (Wetzel et al., 2005) and that its loss from ILC2s was the result of membrane turnover. Trogocytosis of MHCII by ILC2s was assessed by generating mixed bone-marrow chimeras using CD45.1$^+$ WT and CD45.2$^+$ MHCII-deficient cells. Low amounts of MHCII dressing were detected on the ILC2s originating from the MHCII-deficient bone marrow, and this was equivalent to the amount of MHCII acquisition observed on other populations, including B cells (Figure 2G; Figure S3G). Cross-dressing did not account for all MHCII on the surface of ILC2s and, furthermore, ILC2s (confirmed as GATA3$^+$ and CD11c$^-$ compared to DC populations; Figure S3H) expressed mRNA encoding H2-Aa, H2-Ab1, and H2-Eb1, invariant MHCII chaperone CD74, and the MHC transcriptional activator CIITA (Figure 2H). Notably, downregulation of these MHC-related genes coincided with the loss of cell surface MHCII following in vitro culture. Thus, ILC2s express endogenous MHCII and might also acquire this molecule by trogocytosis.

ILC2 Expression of MHCII Is Important for the IL-13-Dependent Expulsion of *N. brasiliensis*

To investigate the biological importance of MHCII expression by ILC2s in vivo, we assessed the effect of deleting MHCII from ILC2s during an *N. brasiliensis* worm infection. Resolution of *N. brasiliensis* infection is highly dependent on ILC2s, which provide the IL-13 that induces the intestinal mucus production and

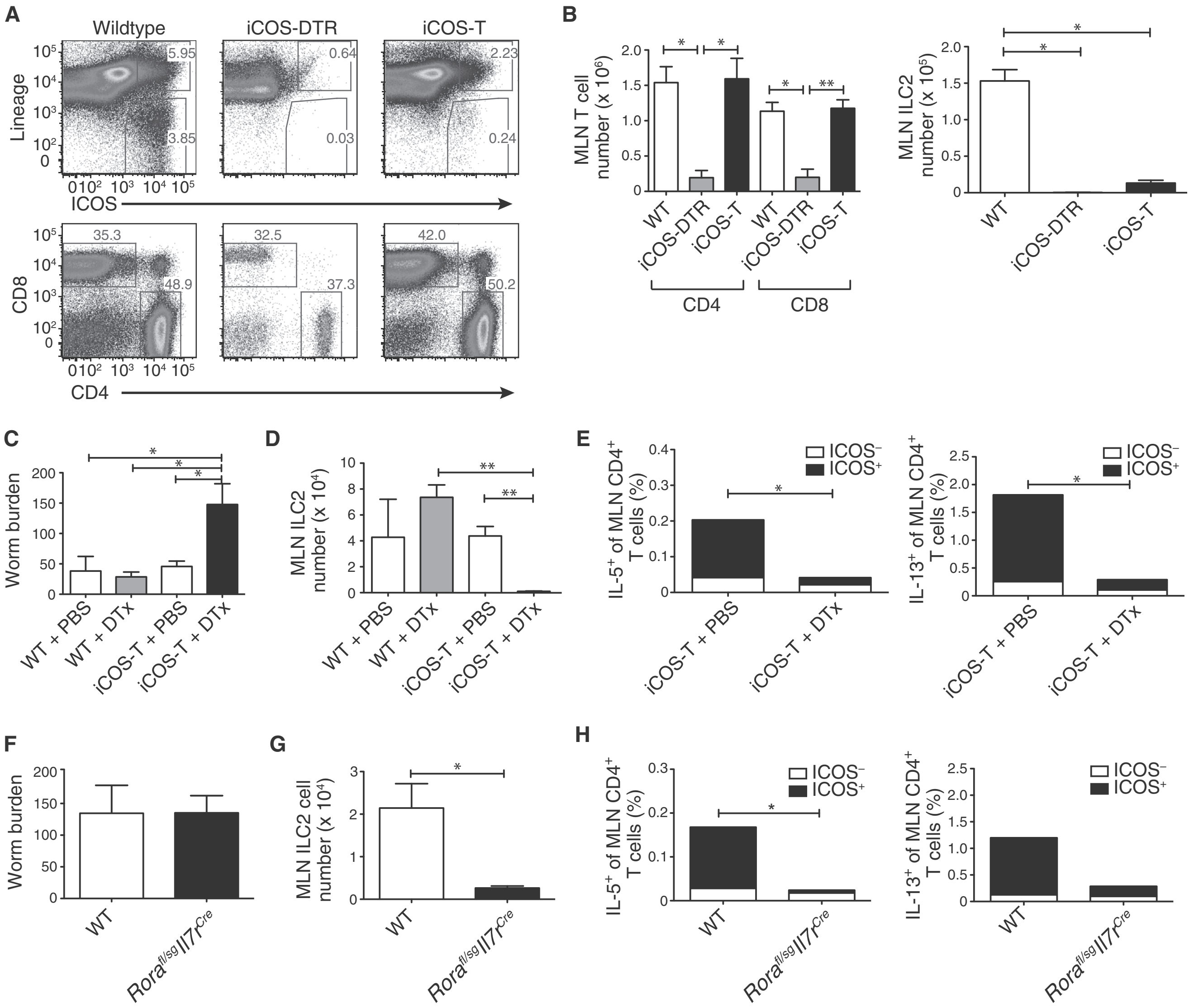

Figure 1. ILC2 Ablation Leads to Impaired Th2 Responses to *N. brasiliensis*

(A) ILC2 ablation. Representative flow cytometric analysis of MLN from WT, iCOS-DTR (without *Cd4*^{Cre}), or iCOS-T mice (with *Cd4*^{Cre}), treated with three daily doses of DTx (25 ng/g bodyweight) and IL-33.

(B) Numbers of T cells and ILC2s in MLN of mice, treated daily with 3 doses of DTx and IL-33.

(C) Worm burden in mice at 5 days postinfection (d.p.i). iCOS-T mice treated daily with DTx (15 ng/g bodyweight, i.p.).

(D) ILC2s in mice 5 d.p.i.

(E) $CD4^{+}$ T cells producing IL-5 and IL-13. Statistical analysis performed between $CD4^{+}ICOS^{+}$ T cell populations (black bars).

(F) Worm burden in *Rora*$^{fl/sg}$*Il7r*^{Cre} mice 5 d.p.i.

(G) ILC2 numbers in *Rora*$^{fl/sg}$*Il7r*^{Cre} mice 5 d.p.i.

(H) $CD4^{+}$ T cells producing IL-5 and IL-13. Statistical analysis performed between $CD4^{+}ICOS^{+}$ T cell populations (black bars).

Data are representative of at least two independent experiments with three (A and B) or five (C–H) mice per group and bar graphs represent mean ± SEM. *$p < 0.05$ and ** < 0.001. See also Figures S1 and S2.

muscular contraction characteristic of the "weep and sweep" response (Fallon et al., 2002). IL-13-deficient hosts (in which the *Il13* gene has been replaced with the eGFP gene [Neill et al., 2010]) are impaired in their ability to expel *N. brasiliensis*, but parasite clearance can be rescued by transferring WT IL-13-sufficient ILC2s into these hosts.

We tested the function of MHCII expression on ILC2s by transferring MHCII-deficient ILC2s into the *N. brasiliensis*-infected IL-13-deficient mice. The transferred ILC2s could be identified in the IL-13-deficient recipients due to expression of IL-13 protein (Figure S4A). Despite having similar intestinal parasite burdens at day 4/5, only IL-13-deficient hosts receiving WT ILC2s expelled their worms efficiently by day 7 (Figure 3A). By contrast, IL-13-deficient hosts receiving MHCII-deficient ILC2s displayed delayed parasite expulsion (Figure 3A). Notably, IL-13-deficient hosts receiving MHCII-deficient ILC2s showed impaired worm

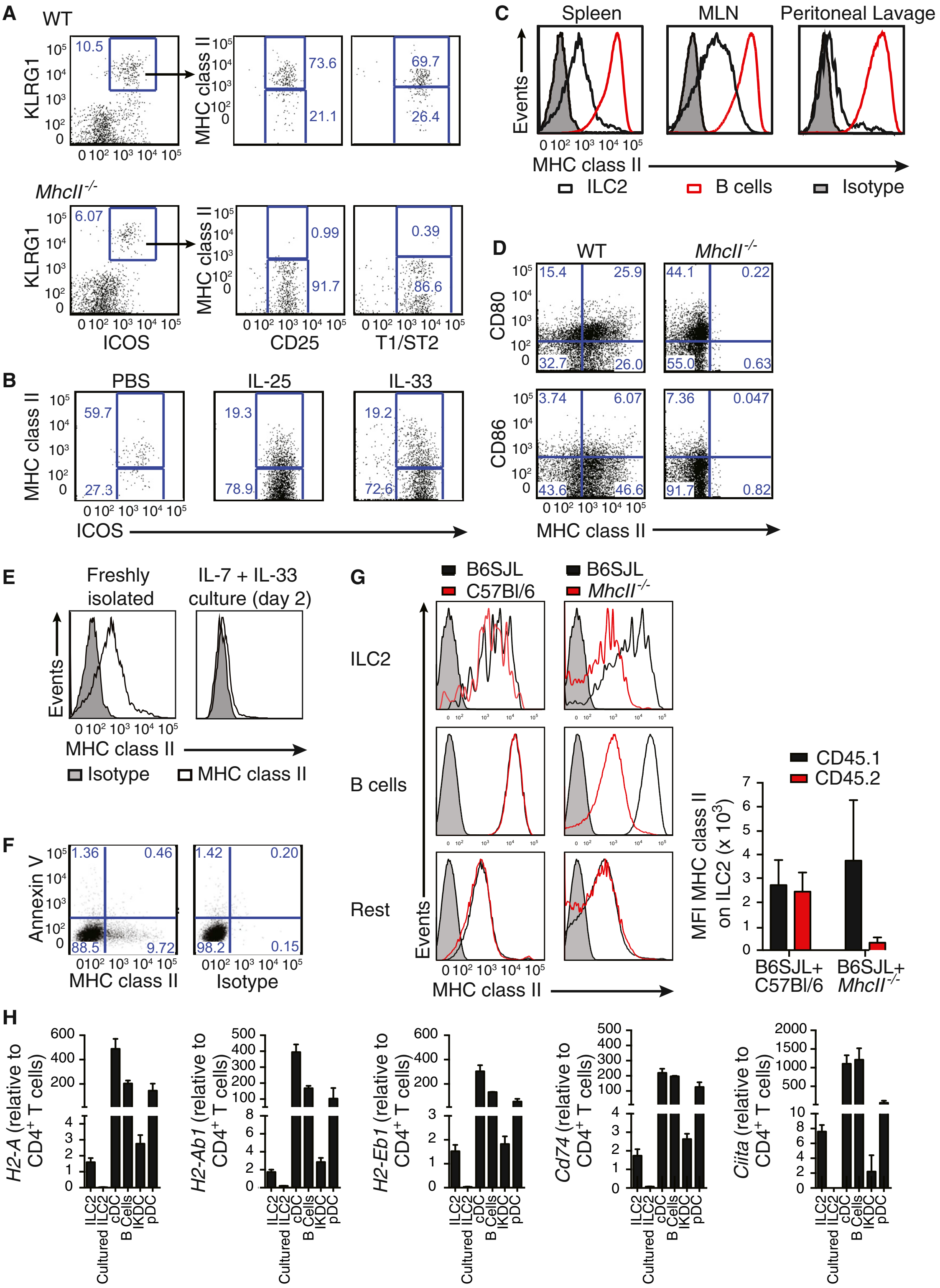

Figure 2. MHCII Is Expressed by ILC2s

(A) Representative flow cytometry of MHCII on ILC2s from the MLN of naive mice. Plots are gated on lin$^-$ cells.

(B) MHCII expression by ILC2s from the MLN of mice treated i.p. as indicated. Plots are gated on lin$^-$ cells.

(C) MHCII expression on IL-33-elicited ILC2s and B cells from the indicated tissues.

(D) CD80 and CD86 expression on Lin$^-$ICOS$^+$ MLN cells from IL-33-treated mice.

(E) MHCII expression on IL-25-elicited ILC2s cultured as indicated.

(legend continued on next page)

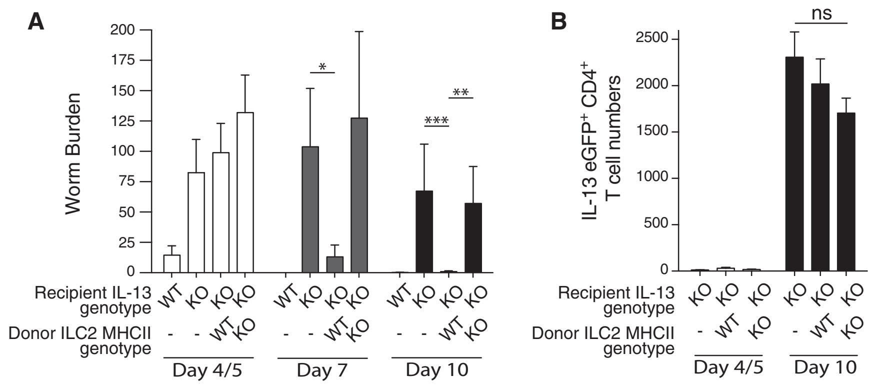

Figure 3. MHCII-Deficient ILC2s Fail to Rescue *N. brasiliensis* Expulsion in IL-13-Deficient Hosts

(A) Worm burden in IL-13-deficient hosts following transfer of ILC2s from IL-25-treated WT or *MhcII*$^{-/-}$ donors. Data are pooled from four independent experiments with five or six mice in each group per experiment. *p < 0.05, **p < 0.01, and ***p < 0.001.
(B) Number of *Il13*$^{egfp/egfp}$ CD4$^+$ T cells in MLN following infection.
Data are representative of three independent experiments with five to six mice per group. Bar graphs represent mean ± SEM. See also Figure S4.

expulsion despite normal, albeit IL-13-deficient, T cell induction (Figure 3B), presumably driven by endogenous MHCII-sufficient APCs. Importantly, we found no intrinsic deficit in the ability of MHCII-deficient ILC2s to produce IL-13 as compared to controls (Figures S4B and S4C). These results suggest a dialog between ILC2s and T cells that is required to induce sufficient IL-13-positive ILC2s.

ILC2 MHCII Contributes to Antigen-Induced Activation of CD4$^+$ T Cells

We next investigated the potential role for MHCII on ILC2s. Purified C57Bl/6 ILC2s were cultured with ovalbumin (OVA)-specific OTII transgenic (Tg) C57Bl/6 CD4$^+$ T cells (Barnden et al., 1998) in the presence or absence of OVA-derived peptide. ILC2s induced in vivo with IL-25 or IL-33 and preincubated with OVA-peptide induced similar T cell proliferation (Figure 4A; Figure S5A). In contrast, CD4$^+$ T cells alone, or cocultures of T cells and ILC2s in the absence of OVA peptide, failed to induce T cell proliferation. ILC2s were purified by flow cytometry to avoid DC cross-contamination (Figure S5B) and limiting dilution analysis confirmed that the contribution of contaminating DCs could be discounted (data not shown). Similar results were obtained with ILC2s purified from BALB/c mice in the presence of BALB/c DO11.10Tg CD4$^+$ T cells (Figure S5C). An irrelevant peptide derived from myelin proteolipid protein failed to stimulate DO11.10Tg T cell proliferation (Figure S5D). ILC2s that had downregulated surface MHCII expression following in vitro culture (Figure 2E) also failed to stimulate DO11.10Tg T cell proliferation (Figure S5E). The inclusion of DCs in these cultures indicated that in vitro cultured ILC2s did not actively impair T cell proliferation (Figure S5E). The peptide-presenting capacity of ILC2s was significantly lower than that of CD11c$^+$ DCs but was equivalent to that observed for naive B cells, IKDCs, and plasmacytoid DCs (Figure 4B).

Unlike ILC2s from WT mice, MHCII-deficient ILC2s failed to induce OTIITg T cell proliferation (Figure 4C). To confirm that the absence of MHCII had not altered the developmental state of ILC2s in the MHCII gene-targeted mice, we also demonstrated that a neutralizing anti-MHCII antibody, but not an isotype control, prevented proliferation of OTIITg T cells cocultured with peptide-pulsed ILC2s (Figures 4D and 4E; Figure S5F). Antigen-dependent T cell activation by ILC2s was further confirmed by increased numbers of activated CD4$^+$ T cells (CD44$^+$CD69$^+$ CD62L$^-$, Figures 4F and 4G). These data demonstrate that ILC2 MHCII ligation induced the expansion and activation of CD4$^+$ T cells.

Because ILC2s express the costimulatory molecules CD80 and CD86 (Figure 2D), we examined the role for these molecules by using blocking antibodies in vitro. Although individual blockade of CD80 or CD86 had little effect on T cell proliferation (Figure 4H), a combination of both antibodies inhibited T cell proliferation as compared to controls (Figure 4H). Antibody blocking was ineffective in BALB/c DO11.10Tg cocultures (data not shown), suggesting potential effects of TCR affinity on the requirement for costimulatory signals. Thus, the expression of the costimulatory molecules CD80 and CD86 by ILC2s provides an additional signal for the proliferation of OTIITg T cells in the context of peptide.

Murine ILC2s Endocytose, Process, and Present Antigen

ILC2s endocytosed soluble antigen in vitro dose dependently (Figures S5G–S5I) and also degraded ovalbumin-DQ (OVA-DQ), a self-quenched conjugate of ovalbumin protein that fluoresces when cleaved (Figures S5H and S5I). Furthermore, in vivo processed OVA-DQ fluorescence was observed in bronchoalveolar lavage and lung-derived ILC2s following intranasal antigen administration (Figures S5J and S5K). However, neither ILC2s loaded in vitro with OVA, or sorted OVA-DQ-positive ILC2s, loaded in vivo, induced transgenic T cell proliferation in coculture (Figures S5L and S5M). To determine whether this resulted from an inability of ILC2s to present the processed antigen, we cultured ILC2s in the presence of an E-alpha (Eα)-green fluorescent protein (GFP) fusion protein and used an Eα-specific

(F) Annexin V and MHCII expression on IL-33-elicited lymph node ILC2s cultured for 48 hr.
(G) Chimeric mice were generated by mixing B6SJL (CD45.1$^+$) bone marrow with either C57Bl/6 or *MhcII*$^{-/-}$ bone marrow (CD45.2$^+$) in a 1:1 ratio. MHCII expression was determined following three doses of IL-33 (i.p.). Graph shows mean fluorescence intensity (MFI) of MHCII expression by ILC2s.
(H) Quantitative RT-PCR for MHCII (*H2-Aa*, *H2-Ab1*, or *H2-Eb1*), *Cd74* and *Ciita* gene expression from the indicated populations. ILC2s are from the MLN of IL-33-treated mice.
Data are representative of at least two independent experiments with three mice per group. Bar graphs represent mean ± SEM. See also Figure S3.

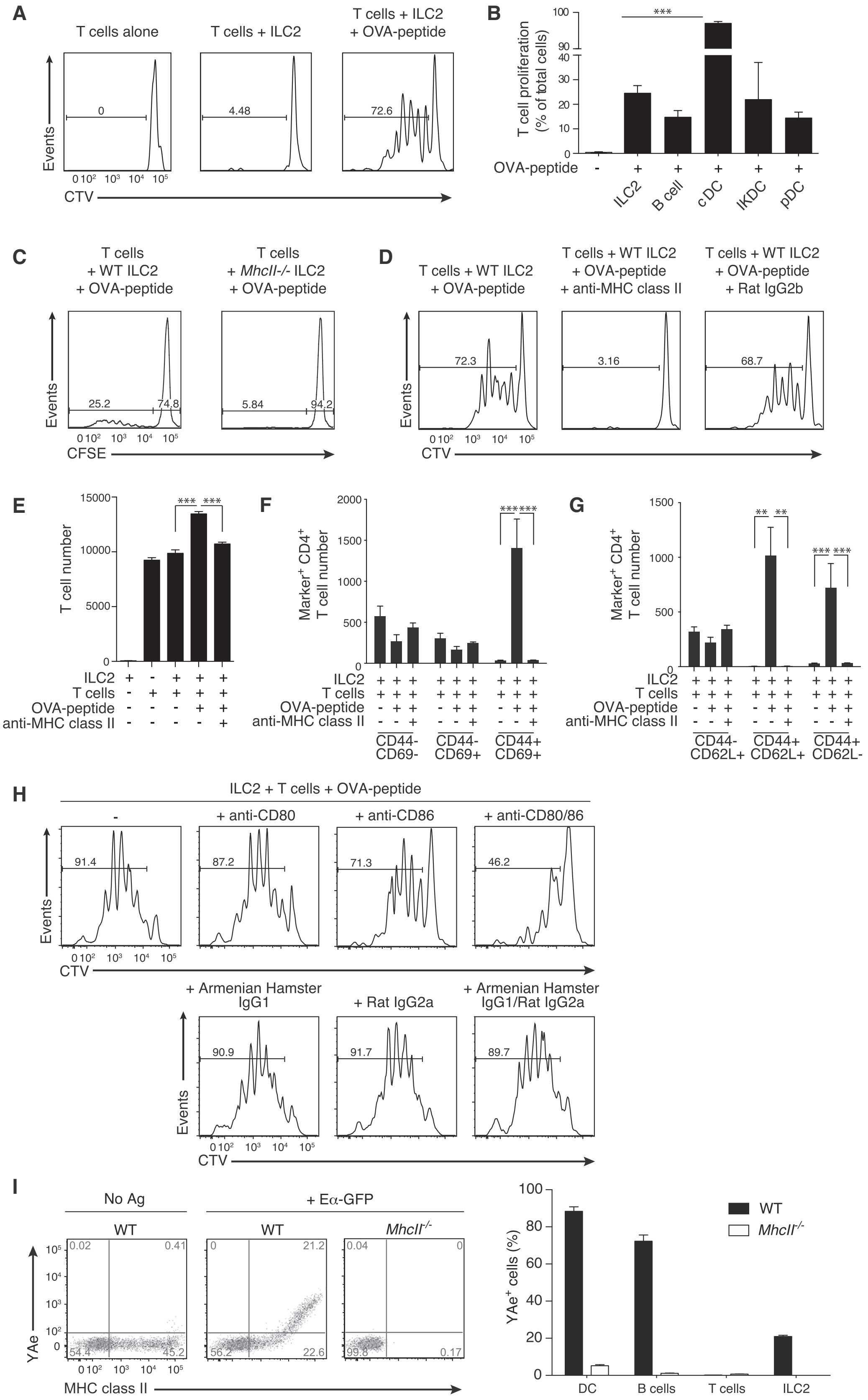

(legend on next page)

antibody to detect an Eα-derived peptide bound in the context of MHC (I-A^b) (Pape et al., 2007). ILC2s were incubated with Eα-GFP and after 20 hr stained for the presence of Eα-derived peptide. Notably, a proportion of the WT, but not MHC-deficient, ILC2s stained positive for the presence of MHCII-bound Eα-peptide as did DCs and B cells (Figure 4I). Thus, ILC2s can process and present antigen, albeit to a lesser extent than professional APCs, but not sufficiently to elicit T cell proliferation in the in vitro assays tested.

ILC2s Enter an Antigen-Dependent Dialog with CD4$^+$ T Cells

We next examined whether ILC2s polarize OTIITg CD4$^+$ T cells toward a Th2 phenotype. Supernatants from OTIITg T cells cocultured with OVA peptide-pulsed ILC2s contained markedly increased amounts of type-2 cytokines IL-5, IL-6, IL-9, and IL-13, but little IFN-γ or IL-17A, as compared to controls lacking peptide (Figure 5A; Figure S6A). This elicitation of type-2 cytokine expression was blocked by neutralizing anti-MHCII antibody (Figure 5A). Notably, this antigen- and MHCII-dependent increase in type-2 cytokines correlated with enhanced ILC2 numbers (Figure 5B).

The source of type-2 cytokines was investigated with cocultures containing either WT ILC2s or ILC2s harvested from mice with a combined deficiency for IL-4, IL-5, IL-9, and IL-13 (QUAD-KO) (Fallon et al., 2002). Coculture of WT ILC2s and DO11.10Tg T cells resulted in IL-5 and IL-13 expression that increased with the inclusion of peptide (Figure 5C). Comparison to parallel cultures, in which QUAD-KO ILC2s were combined with transgenic T cells, demonstrated that the majority of the IL-5 and IL-13 observed in the WT cultures was ILC2-derived (Figure 5C) and that type-2 cytokine production was only induced in T cells following the addition of peptide (Figure 5C). The absence of ILC2-derived type-2 cytokines had no effect on the proliferative potential of T cells (Figure 5D). Intracellular cytokine staining confirmed that ILC2s were the major source of type-2 cytokines following coculture in the presence of OVA peptide (Figure 5E; Figure S6B). Blocking antibodies targeting MHCII (Figure 5E; Figure S6B) or CD80 and CD86 (Figures 5F and 5G) impaired ILC2 expansion and type-2 cytokine expression. Together, these data highlight that ILC2s and T cells enter an antigen-dependent crosstalk that results in their subsequent activation and potentiation of type-2 immune responses and requires the expression of both MHCII and the costimulatory molecules CD80 and CD86 on ILC2s.

Crosstalk between ILC2s and CD4$^+$ T Cells Requires IL-2

We next sought to identify the secreted factors required for ILC2s and CD4$^+$ T cell interaction. Notably, although IL-4, a factor linked to Th2 cell polarization, is not always detectable in ILC2s (Fallon et al., 2006; Neill et al., 2010), we observed that IL-4 production from ILC2s was clearly evident in the presence of peptide (Figure 5C; Figures S6B and S6C). Blocking IL-4 in cocultures had only a small effect on IL-13$^+$ ILC2 and T cell numbers (Figures 6A and 6B). Thus, IL-4 might play a minor role in altering the cytokine milieu that arises in the MHCII-mediated collaboration between ILC2s and T cells.

The limited effect of IL-4 led us to investigate IL-2, the receptor for which (CD25) is highly expressed on ILC2s. Indeed, culture of ILC2s with IL-2 resulted in increased numbers of IL-13-producing ILC2s (Figure 6C). Intracellular cytokine staining demonstrated an antigen-dependent increase in the number of IL-2-expressing T cells in ILC2 cocultures, which was impaired with anti-MHCII antibodies (Figures 6D and 6E). Critically, IL-2 blockade prevented antigen-induced expression of type-2 cytokines in culture supernatants (Figure 6F), and intracellular cytokine staining confirmed a role for IL-2 in the expression of type-2 cytokines from ILC2s (Figure 6G) and T cells (Figure 6H).

We have shown previously that *Rag2*$^{-/-}$ mice have impaired worm clearance. To determine whether this deficit might be due to insufficient IL-2 production from T cells failing to induce sufficient ILC2s, we infected *Rag2*$^{-/-}$ mice with *N. brasiliensis* while also treating one group with an IL-2-anti-IL-2 antibody complex to facilitate slow release of the active IL-2 molecule. *Rag2*$^{-/-}$ mice sufficient for ILC2s failed to expel their worms efficiently (Figure 6I). By contrast, providing IL-2 resulted in rapid worm expulsion that correlated with elevated ILC2 numbers (Figures 6I and 6J). Thus, the MHCII-, CD80-, CD86-initiated production of IL-4 and IL-2 from ILC2s and T cells coordinates efficient T cell and ILC2 proliferation and type-2 cytokine expression.

Human ILC2s Express MHCII and Present Antigen to T Cells

In parallel studies, we assessed MHCII expression on purified human lineage$^-$IL-7Rα$^+$CRTH2$^+$ ILC2s (Mjösberg et al., 2011; Salimi et al., 2013) (Figure S6D). Human ILC2s expressed high amounts of HLA-DR, comparable to human monocytes, in conjunction with the costimulatory receptors CD80 and CD86 (Figure S6E). We investigated the ability of human ILC2s to activate human T cell lines with antigen specificity for the common house dust-mite allergen (HDM) Der p 1. Increasing concentrations of whole Der p 1 protein or Der p 1 peptide (DRB15), induced a 100- to 200-fold increase in the expression of IL-4 (Figure S6F), accompanied by a 3-fold increase in IFN-γ production (Figure S6G). Intracellular cytokine staining revealed induction of T cell-derived IL-13 in HLA-DR$^+$ ILC2 cocultures, but not those with anti-MHCII or HLA-mismatched ILC2s (Figure S6H). However, ILC2-produced IL-13 was not detected (Figure S6H).

Figure 4. Antigen-Dependent Activation of T Cells by ILC2s Requires MHCII

(A) Cell trace violet (CTV)-labeled OTIITg CD4$^+$ T cells were cultured in a 1:1 ratio with IL-33-elicited ILC2s, pulsed with or without OVA-peptide.

(B) Antigen-dependent OTIITg CD4$^+$ T cell proliferation induced by the indicated populations.

(C) CFSE-labeled OTIITg CD4$^+$ T cell proliferation, as indicated.

(D) CTV-labeled OTIITg CD4$^+$ T cell proliferation, as indicated.

(E) OTIITg CD4$^+$ T cell numbers in cocultures, as indicated.

(F and G) The number of activated OTIITg CD4$^+$ T cells (CD44$^+$CD69$^+$ or CD44$^+$CD62L$^-$) following coculture with antigen-pulsed ILC2s.

(H) CTV-labeled OTIITg CD4$^+$ T cell proliferation, as indicated. Data are representative of three independent experiments with two or three mice per group.

(I) Detection of processed and presented Eα peptide on ILC2s. Data are representative of two independent experiments.

ILC2s were elicited using IL-33 and isolated from mesenteric, para-aortic, and inguinal lymph nodes. Bar graphs represent mean ± SEM. See also Figure S5.

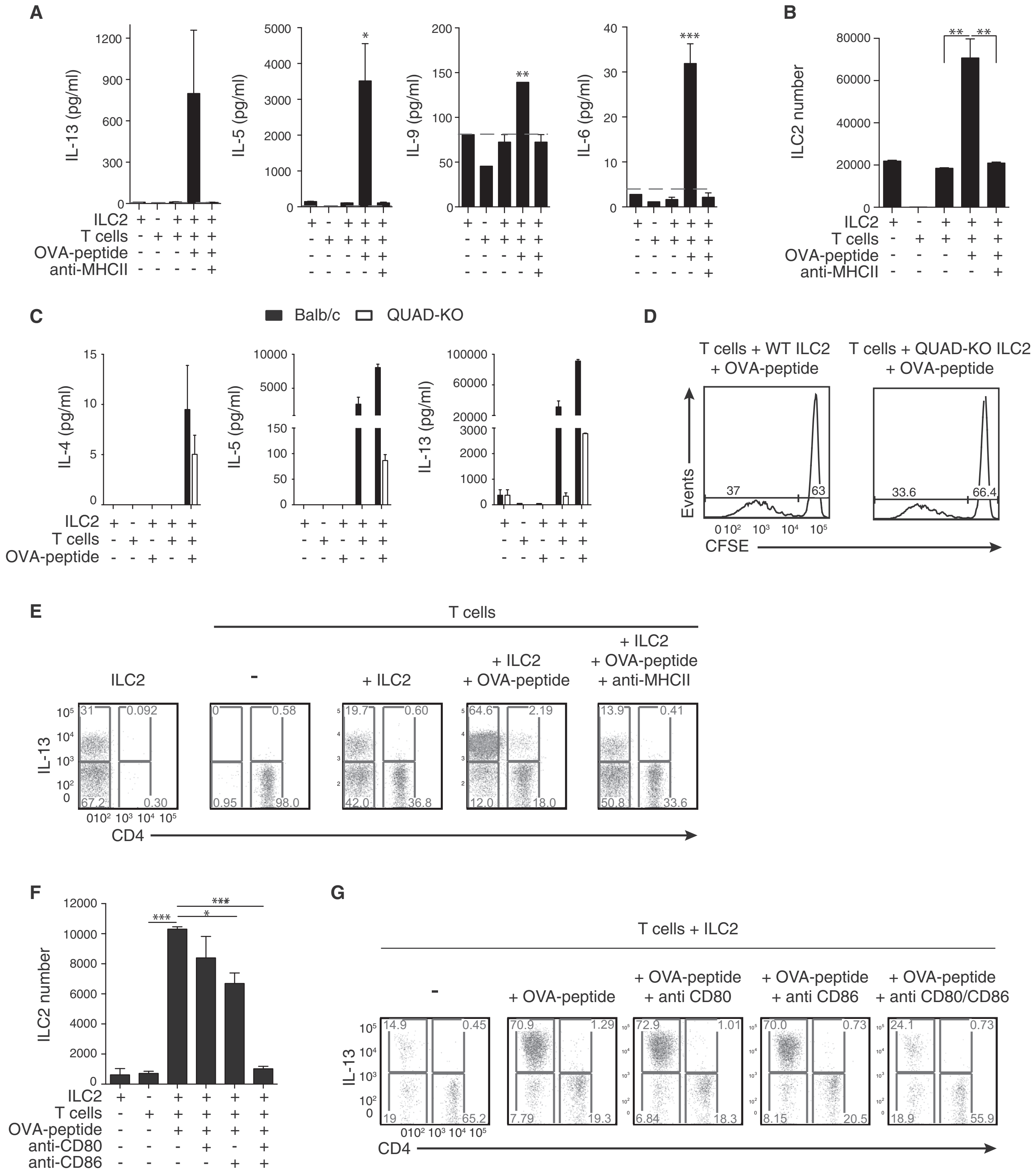

Figure 5. ILC2s Enter Antigen-Dependent Dialog with CD4⁺ T Cells

(A) Cytokines in supernatants of cocultures containing peptide-pulsed ILC2s and OTIITg CD4⁺ T cells. Dotted line indicates limit of detection for each assay.

(B) ILC2 number following coculture with OTIITg CD4⁺ T cells, as indicated.

(C) Cytokine production in supernatants of WT (BALB/c) or QUAD-KO ILC2s and DO11.10Tg T cell cocultures, as indicated.

(D) CFSE-labeled DO11.10Tg T cell proliferation following coculture with QUAD-KO ILC2s, as indicated.

(E) Intracellular IL-13 staining following ILC2:OTIITg CD4⁺ T cell coculture, as indicated.

(legend continued on next page)

Thus, human ILC2s can process and present antigens to T cells thereby initiating cytokine expression.

DISCUSSION

A critical role for ILC2s in antihelminthic immune responses was demonstrated in mice deficient in IL-33 and IL-25 signaling (Neill et al., 2010) and in RORα-deficient mice, which lack ILC2s (Wong et al., 2012). Transfer of ILC2s into IL-13-deficient, but not Rag2-deficient, hosts was sufficient to restore worm clearance (Neill et al., 2010). This suggested that interactions between IL-13-sufficient ILC2s and T cells, but probably not B cells (Voehringer et al., 2006), were required for the effective clearance of parasitic helminths. The impairment of T cell responses suggested that in addition to conventional T cell priming by DCs, which is essential for efficient Th2 generation (Hammad et al., 2010; Phythian-Adams et al., 2010), ILC2s might also play a previously unanticipated role in the initiation of protective type-2 immunity.

We have generated two ILC2-depletion models to study ILC2 function in vivo. The iCOS-T model relies upon *Icos*-promoter controlled expression of DTR on ILC2s enabling temporal regulation of ILC2 deletion by DTx-administration. In the second model, ILC2s were depleted through IL-7Rcre-mediated excision of a "floxed" exon in the *Rora* gene. In these distinct models, we observed that ILC2 ablation impaired the magnitude of the Th2 response during *N. brasiliensis* infection.

While MHCII-expressing conventional antigen-presenting DCs are essential for activating naive T cell responses (Hammad et al., 2010; Phythian-Adams et al., 2010), MHCII-mediated crosstalk between ILC2s and T cells plays an additional role in potentiating IL-13-dependent *N. brasiliensis* expulsion. T cell-derived IL-2 induces proliferation of ILC2s and elevates their expression of type-2 cytokines. Thus, this reciprocal interaction potentiates both the innate and adaptive sources of type-2 cytokines and provides an important antigen-dependent feedback, whereby regulation of ILC2 proliferation or survival shifts from innate sources of IL-33 and/or IL-25 (Neill et al., 2010) to cytokines provided by an ongoing adaptive response, namely IL-2 and IL-4 derived from T cells.

Several pathways regulate Th2 cell differentiation, including TCR signal strength, ligation of costimulatory molecules on APCs, and availability of cytokines such as IL-2 and IL-4 (Cote-Sierra et al., 2004; Jenkins et al., 2008; Yamane et al., 2005). Coculture of T cells with ILC2s, in the presence of antigen, preferentially induces T cell secretion of IL-5 and IL-13. Notably, we observed relatively low amounts of MHCII expression on ILC2s, compared to DC subsets or B cells. This interaction might contribute to the polarization of the T cell response because suboptimal TCR ligation has been reported to bias Th2 differentiation in vitro (Yamane et al., 2005) and in vivo (Jun et al., 2003). Similarly, components from *Schistosoma mansoni* egg extracts have been demonstrated to condition DCs, resulting in their impaired interaction with T cells and potentially reduced TCR signal strength (Everts et al., 2009). Therefore, ILC2s, unlike other APC, might be preconditioned to polarize T cells toward a Th2 phenotype by virtue of their lower amount of MHCII expression.

Cytokine regulation of Th2 cell differentiation has focused on IL-4 though it is clear that it is not essential for Th2 development in vivo (van Panhuys et al., 2008). Indeed, we found little role for IL-4 in the ILC2-regulated differentiation of Th2 cells. By contrast, IL-2 was critical for the proliferation of Th2 cells in the presence of ILC2s and antigen. IL-2 is expressed by T cells following CD28 ligation (Seder et al., 1994) and plays an important role in Th2 differentiation via STAT5a signaling (Cote-Sierra et al., 2004; Zhu et al., 2003). Notably, we found that the CD28 ligands CD80 and CD86 were expressed on ILC2, suggesting that ILC2s have the capacity to interact with T cells and contribute to their type-2 polarization.

Strikingly, we observed a profound increase in ILC2 proliferation and type-2 cytokine production in T cell cocultures with antigen, which was dependent on T cell-derived IL-2. ILC2s require the common γ-chain (γc) receptor for their development and proliferation, and are absent in γc receptor-deficient mice (Moro et al., 2010; Neill et al., 2010; Price et al., 2010). Furthermore, Moro et al. (2010) showed that ILC2s from fat-associated lymphoid clusters (FALC) expanded in the presence of IL-2, resulting in elevated amounts of cytokine production (Moro et al., 2010). Additionally, IL-2 has also been reported to induce ILC2s to secrete IL-9, which then protects the ILC2s from apoptosis via an autocrine feedback loop (Turner et al., 2013). Therefore, it is possible that IL-2 expressed following antigen-dependent ILC2:T cell interactions induces IL-9 expression and improves ILC2 survival, thereby further biasing toward a type-2 immune response. Certainly, IL-9 was present in the cultures that included T cells and antigen, though we were unable to identify the cellular source.

Despite demonstrating that murine ILC2s can take up and process OVA, and process and present Eα-GFP, we have been unable to demonstrate T cell proliferation. Several studies have shown that cells can acquire MHCII:antigen complexes from antigen-presenting cells (Wetzel et al., 2005). We observed acquisition of MHCII by ILC2s in mixed bone-marrow chimera experiments, which might contribute to their biology. However, where MHCII-deficient ILC2s were transferred into *N. brasiliensis*-infected MHCII-sufficient mice, MHCII trogocytosis was insufficient to restore worm expulsion. In contrast to mouse ILC2s, we found that human ILC2s process whole Der p 1 antigen and present the derived peptides to T cells in vitro. Thus, our existing in vitro cellular proliferation assays appear too insensitive. Previously, human NK cells have been shown to process and present tetanus toxoid and Der p 1 to T cells, but protein antigen from *Mycobacterium leprae* was poorly processed and presented (Roncarolo et al., 1991). It is also noteworthy that many of the antigens associated with type-2 immune responses are either shed by multicellular helminth parasites (Kamata et al., 1995) or are proteins with protease activity, such as Der p 1 or papain (Gough et al., 1999; Halim et al.,

(F) ILC2 number following coculture with OTIITg CD4$^+$ T cells, as indicated.

(G) Intracellular IL-13 staining following ILC2:OTIITg CD4$^+$ T cell coculture, as indicated.

Data are representative of three independent experiments with two or three mice per group. MLN ILC2s were elicited using IL-33 (A, B, E–G) or IL-25 (C and D). Bar graphs represent mean ± SEM. See also Figure S6.

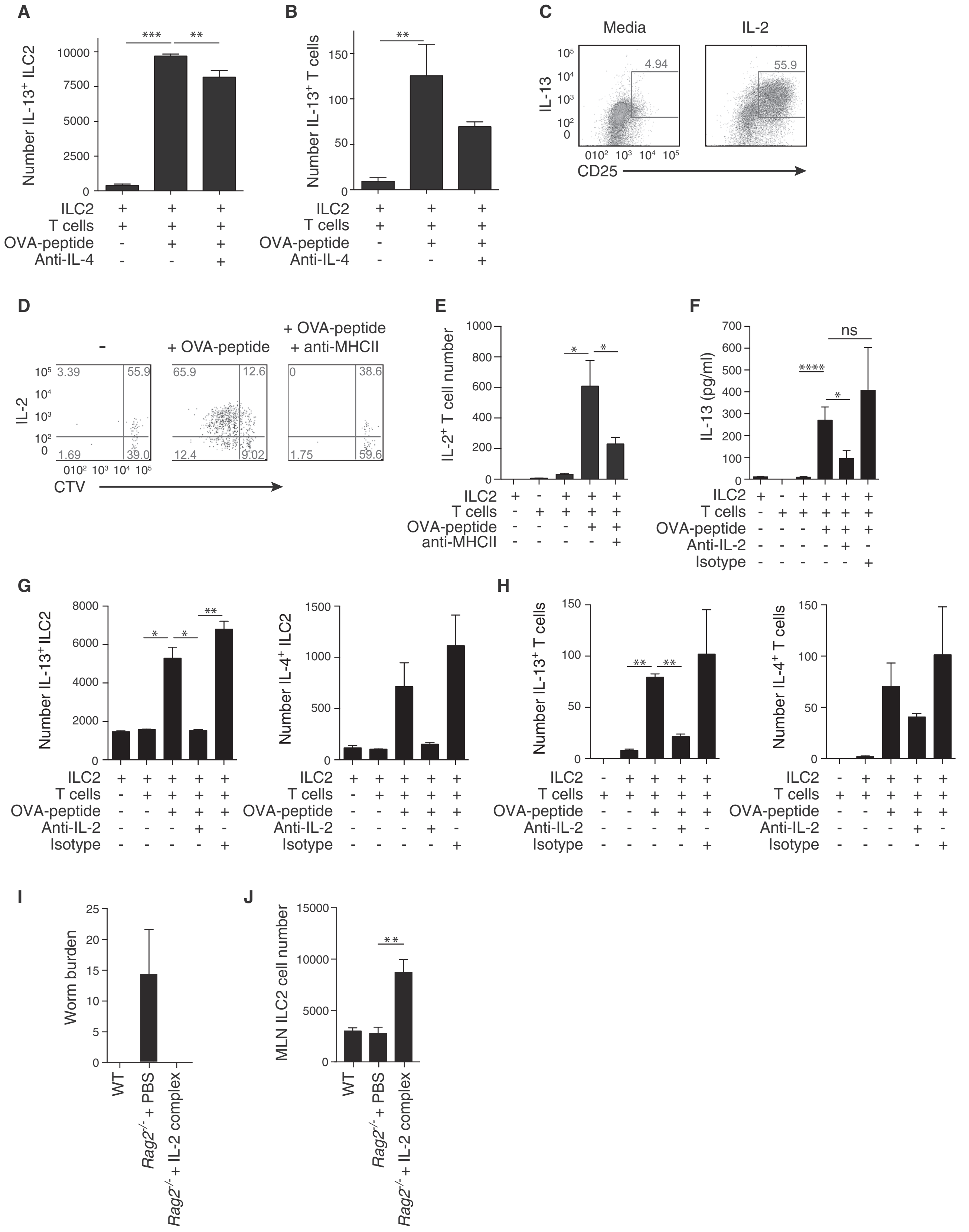

(legend on next page)

2012). It is therefore interesting to speculate that ILC2s might also acquire exogenous peptides from their surroundings (Santambrogio et al., 1999).

This proinflammatory role for MHCII expression on ILC2s supports similar findings for peptide presentation by ILC2s (Mirchandani et al., 2014). By contrast, ILC3s have been reported to suppress intestinal immune responses against commensal bacteria (Hepworth et al., 2013). Notably, ILC2s express the costimulatory molecules CD80 and CD86 on their surface, whereas ILC3s do not (Hepworth et al., 2013), and this might underlie the agonistic role for MHCII on ILC2s.

In addition to antihelminthic immunity, ILC2s have also been implicated in promoting detrimental type-2 immune responses in mouse models of asthma (Barlow et al., 2012; Chang et al., 2011; Monticelli et al., 2011) and allergies (Bartemes et al., 2012; Halim et al., 2012; Salimi et al., 2013). Currently there is little information regarding ILC2s and T cell interactions in allergic models, but similar antigen-dependent ILC2:T cell crosstalk might also be important for the potentiation of type-2 responses in allergy. Indeed, ILC2s are enriched in nasal polyps from patients with chronic rhinosinusitis (Mjösberg et al., 2011), which might arise in response to allergens. We have now demonstrated the ability of human ILC2s to process and present the house-dust-mite allergen Der p 1, to human T cells in vitro in a process that would exacerbate type-2 inflammation. In these human assays, we observed the induction of T cell-derived IL-13, but did not detect ILC2-produced IL-13. It is not clear why this is the case but might be a consequence of the 3- to 4-week culture required to derive sufficient ILC2s for these assays. However, these data support a potential role for human ILC2s in the potentiation of T cell responses during asthma and allergy.

The two mouse models that we describe for the genetic ablation of ILC2s in vivo complement those that have already been reported. The use of the *Il13* gene to drive diphtheria toxin resulted in the deletion of IL-13-producing ILC2s, but other IL-13-producers would also be susceptible to toxin-mediated cell death (Liang et al., 2012). The depletion of ILC2s has also been reported in mice in which *Id2*^{CreER} mediated the excision of a conditional *Gata3* allele (Hoyler et al., 2012); however, it is becoming clear that GATA3 is required for all ILC populations (Serafini et al., 2014). We now show that ILC2 ablation can be regulated temporally in otherwise immune-competent iCOS-T mice. Because it remained possible that the insertion of DTR into the *Icos* locus might alter T cell activation, we also generated *Rora*$^{fl/sg}$*Il7r*^{Cre} mice. Although RORα has been reported previously to play a subordinate role to RORγ in the development of Th17 T cells, a role in Th2 cell activation has not been reported. Thus, the complementary data from these two distinct mouse lines strongly support the role for ILC2s in the progression to adaptive Th2-mediated immunity. These mice should prove useful for defining the roles of ILC2s in further disease models.

Our data demonstrate that ILC2s and T cells cooperate through MHCII-dependent activation to potentiate the type-2 response against *N. brasiliensis* and extend the recently described pathway by which IL-13 from ILC2s can promote DC migration to the draining lymph nodes to stimulate Th2 polarization (Halim et al., 2014). Such functions place ILC2s at a critical point in the transition from the innate type-2 response to adaptive type-2 immunity. Therefore, ILC2s expand initially in response to IL-33 and IL-25 derived from innate cell sources but require adaptive T cell-produced mediators for their maintenance and subsequent potentiation of protective type-2 immunity.

EXPERIMENTAL PROCEDURES

Mice

C57Bl/6, *H2-Ab1*$^{-/-}$ (*MhcII*$^{-/-}$) (Cosgrove et al., 1991), *Il13*$^{egfp/egfp}$ (Neill et al., 2010), *Il7r*^{Cre} (Schlenner et al., 2010), OTIITgB6 (JAX Laboratories), and Staggerer *Rora*$^{sg/+}$ (JAX Laboratories) mice were on a C57Bl/6 background. *Il13*$^{tdTomato/+}$, *Il4*$^{gfp/gfp}$ (Hu-Li et al., 2001) and *Il4*$^{-/-}$*Il5*$^{-/-}$*Il9*$^{-/-}$*Il13*$^{-/-}$ (QUAD-KO) (Fallon et al., 2002) mice were on a BALB/c background. iCOS-DTR, iCOS-T, and *Rora*$^{+/flox}$*Il7r*^{Cre} mice were generated as described in the Supplemental Experimental Procedures. All mice were bred in a specific pathogen-free facility. BALB/c mice were purchased from Charles River Laboratories as required. In individual experiments all mice were matched for age, gender, and background strain. All animal experiments undertaken in this study were done so with the approval of the UK Home Office.

IL-25, IL-33, IL-2 Complex and DTx Administration

IL-25 (either 2 μg/mouse or 0.5 μg/mouse of recombinant mouse IL-25 [Centocor] in PBS) was administered daily for 3 days intraperitoneally (i.p., termed IL-25-elicited ILC2s). For i.p. IL-33 administration, 0.5 μg/mouse or 1 μg/mouse recombinant mouse IL-33 (BioLegend) in PBS was administered daily for up to 4 days (termed IL-33-elicited ILC2s). For intranasal IL-33 administrations, mice were anesthetized and 0.4 μg of recombinant protein administered in PBS on 4 consecutive days. IL-2 complexes were prepared by incubating IL-2 and anti-IL-2 mAb (clone JES6) (both from R&D Systems) at a 1:5 w/w ratio for 20 min at 37°C. Complexes were then diluted in PBS and administered i.p. (1 μg IL-2 and 5 μg anti-IL-2 per dose) on days 0, 2, 4, 6, and 8 following *N. brasiliensis* infection. DTx (either 25 ng/g or 15 ng/g body weight, Sigma) was administered daily for up to 5 days i.p. Mice were euthanized 24 hr later and tissues collected for analysis. Control animals received PBS only.

Bone-Marrow Chimeras

For mixed-chimera experiments, lethally irradiated CD45.1^{+} B6/SJL mice were reconstituted with a 1:1 mixture of bone-marrow cells (2 × 10^{6} cells) containing either CD45.1^{+} B6/SJL and CD45.2^{+} C57Bl/6 or CD45.1^{+} B6/SJL and CD45.2^{+}

Figure 6. IL-2 Is Required for the Antigen-Dependent Dialog between ILC2s and T Cells

(A and B) Number of IL-13-expressing ILC2s (A) and T cells (B) from ILC2:OTIITg CD4^{+} T cell cocultures in the presence of IL-4-blocking antibodies.
(C) Surface CD25 and intracellular IL-13 expression by ILC2s stimulated with IL-2 for 72 hr.
(D) Intracellular IL-2 staining of CTV-labeled OTIITg CD4^{+} T cells following coculture with OVA-peptide-loaded WT ILC2s.
(E) Number of IL-2-expressing T cells from ILC2:OTIITg CD4^{+} T cell cocultures in the presence of MHCII-blocking antibodies.
(F) IL-13 concentration in supernatants from ILC2:OTIITg CD4^{+} T cell cocultures in the presence of blocking anti-IL-2 antibodies.
(G and H) Number of cytokine expressing ILC2s (G) and OTIITg CD4^{+} T cells (H) in the presence of IL-2 blocking antibodies.
(I) Worm burdens and (J) ILC2s in *Rag2*$^{-/-}$ mice 10 d.p.i. with *N. brasiliensis* and treated as indicated.
Lymph node ILC2s were elicited with IL-33. Bar graphs represent mean ± SEM. Data are representative of three independent experiments with two or three mice per group (A–H) or from a single experiment with five or six mice per group (I and J). Bar graphs represent mean ± SEM.

MhcII$^{-/-}$ cells. After 10 weeks, mice were given daily doses of recombinant mouse IL-33 (0.4 μg per dose).

In Vitro ILC2 Cultures with IL-7 and IL-33 or IL-2

For the expansion of ILC2s in vitro from WT mice, ILC2s were FACS purified, as defined by LIN$^-$ (a combination of CD3, CD4, CD8α, CD19, B220, CD11c, CD11b, Gr1, FcεR1, CD5, γ/δTCR, NK1.1, and TER119) ICOS$^+$. For IL-7/IL-33 in vitro cultures of ILC2s, we followed the protocol as described previously (Neill et al., 2010). Cultures were maintained for between 2 and 6 days. For in vitro IL-2 cultures, ILC2s were FACS purified from the spleen and MLN of IL-25 or IL-33-treated mice, and subsequently cultured at 2.5 × 10^4 cells per well in the presence or absence of IL-2 (10 ng/ml). Cultures were maintained for 3 days at 37°C.

In Vitro ILC2s and T Cell Cocultures

Freshly isolated or in vitro cultured ILC2s, from WT, *MhcII*$^{-/-}$, or QUAD-KO mice, were resuspended in RPMI 1640 (GIBCO®) supplemented with 2 mM L-glutamine, 50 U/ml penicillin, 50 μg/ml streptomycin, and 10% Hyclone Fetal Bovine Serum (Thermoscientific). Where necessary, ILC2s were pulsed with OVA-peptide (323–339) for 2 hr at 37°C. Subsequently ILC2s were washed thoroughly and 1 × 10^4 ILC2s were cultured with 1 × 10^4 labeled CD3$^+$CD4$^+$ OTIITg or DO11.10Tg splenic T cells, which were sorted by flow cytometry, for 5 days at 37°C, 5% CO_2. Where indicated, the antibodies M5/114.15.2 (anti-MHCII, eBioscience), 16-10A1 (anti-CD80, BioLegend), and GL-1 (anti-CD86, BioLegend) were added to cultures at a concentration of 1 μg/ml. 11B11 (anti-IL-4, eBioscience) was incubated at a concentration of 10 μg/ml. Where appropriate, blockade of IL-2 was performed with 10 μg/ml of JES6-1A12 (eBioscience) and S4B6 (BD PharMingen). Cocultures containing in vivo-loaded OVA-DQ$^+$ ILC2s were prepared by FACS-purifying ILC2s, based on Lin$^-$ICOS$^+$OVA-DQ$^+$ or lineage$^-$ICOS$^+$OVA-DQ$^-$, from the bronchoalveolar lavage of WT mice receiving an intranasal administration of OVA-DQ and IL-33 on 7 consecutive days. Cocultures were setup at a 1:1 ratio with 4 × 10^4 cells per well. Human samples were taken under GCP guidance with ethical approval of the NRES Committee South Central.

Helminth Infection and Cell Transfers

Mice were inoculated subcutaneously with 500 viable third-stage *N. brasiliensis* larvae. Where appropriate, FACS-purified ILC2s were transferred by intravenous injection 2 hr after *N. brasiliensis* injections.

E-alpha:Green Fluorescent Protein Fusion Protein

To assess the ability of ILC2s to present processed antigen, we cultured ILC2s in the presence of 100 μg/ml E-alpha (Eα)-green fluorescent protein (GFP) fusion protein for 20 hr. An Eα-specific antibody (clone YAe) was used to detect an Eα-derived peptide (Eα52-68) bound in the context of MHC (I-A^b) prior to flow cytometric analysis.

Statistical Analysis

Graph Pad Prism was used to calculate the SEM when different numbers of data sets existed in each experimental group. When data were normally distributed and when two independent variables were being analyzed, a Kruskal-Wallis one-way ANOVA with Bonferroni post-analysis was performed. In all other instances, statistical differences between groups were calculated with Student's t test, with $P < 0.05$ considered significant.

SUPPLEMENTAL INFORMATION

Supplemental Information includes six figures and Supplemental Experimental Procedures and can be found with this article online at http://dx.doi.org/10.1016/j.immuni.2014.06.016.

AUTHOR CONTRIBUTIONS

A.N.J.M., S.H.W, Y.Y.H., and C.J.O conceived the study. C.J.O, S.H.W., J.A.W., J.L.B., Y.Y.H., E.H., A.E., S.T.S., and P.G.F. did the experiments or contributed to experimental design, reagents, and analysis. G.S.O and M.S. performed the human ILC2 experiments. A.N.J.M. wrote the manuscript with contributions from all authors, but primarily from C.J.O and J.A.W.

ACKNOWLEDGMENTS

We thank the Ares staff for their technical assistance. We also thank A. Betz for providing MHCII-deficient mice. This work was supported by the American Asthma Foundation (A.N.J.M.), UK-MRC (A.N.J.M., G.S.O.), Wellcome Trust (grant number 100963/Z/13/Z) (A.N.J.M.), A-STAR (Y.Y.H.), Science Foundation Ireland and National Children's Research Centre (P.G.F.), and NIHR Biomedical Research Centre Programme (G.S.O.). A.N.J.M. and G.S.O. have research grant funding from Janssen Research and Development.

Received: December 11, 2013
Accepted: June 25, 2014
Published: July 31, 2014

REFERENCES

Barlow, J.L., Bellosi, A., Hardman, C.S., Drynan, L.F., Wong, S.H., Cruickshank, J.P., and McKenzie, A.N. (2012). Innate IL-13-producing nuocytes arise during allergic lung inflammation and contribute to airways hyperreactivity. J. Allergy Clin. Immunol. *129*, 191–198, e191-194.

Barnden, M.J., Allison, J., Heath, W.R., and Carbone, F.R. (1998). Defective TCR expression in transgenic mice constructed using cDNA-based alpha- and beta-chain genes under the control of heterologous regulatory elements. Immunol. Cell Biol. *76*, 34–40.

Bartemes, K.R., Iijima, K., Kobayashi, T., Kephart, G.M., McKenzie, A.N., and Kita, H. (2012). IL-33-responsive lineage- CD25+ CD44(hi) lymphoid cells mediate innate type 2 immunity and allergic inflammation in the lungs. J. Immunol. *188*, 1503–1513.

Chan, C.W., Crafton, E., Fan, H.N., Flook, J., Yoshimura, K., Skarica, M., Brockstedt, D., Dubensky, T.W., Stins, M.F., Lanier, L.L., et al. (2006). Interferon-producing killer dendritic cells provide a link between innate and adaptive immunity. Nat. Med. *12*, 207–213.

Chang, Y.J., Kim, H.Y., Albacker, L.A., Baumgarth, N., McKenzie, A.N., Smith, D.E., Dekruyff, R.H., and Umetsu, D.T. (2011). Innate lymphoid cells mediate influenza-induced airway hyper-reactivity independently of adaptive immunity. Nat. Immunol. *12*, 631–638.

Cosgrove, D., Gray, D., Dierich, A., Kaufman, J., Lemeur, M., Benoist, C., and Mathis, D. (1991). Mice lacking MHC class II molecules. Cell *66*, 1051–1066.

Cote-Sierra, J., Foucras, G., Guo, L., Chiodetti, L., Young, H.A., Hu-Li, J., Zhu, J., and Paul, W.E. (2004). Interleukin 2 plays a central role in Th2 differentiation. Proc. Natl. Acad. Sci. USA *101*, 3880–3885.

Everts, B., Perona-Wright, G., Smits, H.H., Hokke, C.H., van der Ham, A.J., Fitzsimmons, C.M., Doenhoff, M.J., van der Bosch, J., Mohrs, K., Haas, H., et al. (2009). Omega-1, a glycoprotein secreted by Schistosoma mansoni eggs, drives Th2 responses. J. Exp. Med. *206*, 1673–1680.

Fallon, P.G., Jolin, H.E., Smith, P., Emson, C.L., Townsend, M.J., Fallon, R., Smith, P., and McKenzie, A.N. (2002). IL-4 induces characteristic Th2 responses even in the combined absence of IL-5, IL-9, and IL-13. Immunity *17*, 7–17.

Fallon, P.G., Ballantyne, S.J., Mangan, N.E., Barlow, J.L., Dasvarma, A., Hewett, D.R., McIlgorm, A., Jolin, H.E., and McKenzie, A.N. (2006). Identification of an interleukin (IL)-25-dependent cell population that provides IL-4, IL-5, and IL-13 at the onset of helminth expulsion. J. Exp. Med. *203*, 1105–1116.

Forbes, E., van Panhuys, N., Min, B., and Le Gros, G. (2010). Differential requirements for IL-4/STAT6 signalling in CD4 T-cell fate determination and Th2-immune effector responses. Immunol. Cell Biol. *88*, 240–243.

Gough, L., Schulz, O., Sewell, H.F., and Shakib, F. (1999). The cysteine protease activity of the major dust mite allergen Der p 1 selectively enhances the immunoglobulin E antibody response. J. Exp. Med. *190*, 1897–1902.

Halim, T.Y., Krauss, R.H., Sun, A.C., and Takei, F. (2012). Lung natural helper cells are a critical source of Th2 cell-type cytokines in protease allergen-induced airway inflammation. Immunity *36*, 451–463.

Halim, T.Y., Steer, C.A., Mathä, L., Gold, M.J., Martinez-Gonzalez, I., McNagny, K.M., McKenzie, A.N., and Takei, F. (2014). Group 2 innate

lymphoid cells are critical for the initiation of adaptive T helper 2 cell-mediated allergic lung inflammation. Immunity *40*, 425–435.

Hammad, H., Plantinga, M., Deswarte, K., Pouliot, P., Willart, M.A., Kool, M., Muskens, F., and Lambrecht, B.N. (2010). Inflammatory dendritic cells—not basophils—are necessary and sufficient for induction of Th2 immunity to inhaled house dust mite allergen. J. Exp. Med. *207*, 2097–2111.

Hepworth, M.R., Monticelli, L.A., Fung, T.C., Ziegler, C.G., Grunberg, S., Sinha, R., Mantegazza, A.R., Ma, H.L., Crawford, A., Angelosanto, J.M., et al. (2013). Innate lymphoid cells regulate CD4+ T-cell responses to intestinal commensal bacteria. Nature *498*, 113–117.

Hoyler, T., Klose, C.S., Souabni, A., Turqueti-Neves, A., Pfeifer, D., Rawlins, E.L., Voehringer, D., Busslinger, M., and Diefenbach, A. (2012). The transcription factor GATA-3 controls cell fate and maintenance of type 2 innate lymphoid cells. Immunity *37*, 634–648.

Hu-Li, J., Pannetier, C., Guo, L., Löhning, M., Gu, H., Watson, C., Assenmacher, M., Radbruch, A., and Paul, W.E. (2001). Regulation of expression of IL-4 alleles: analysis using a chimeric GFP/IL-4 gene. Immunity *14*, 1–11.

Jenkins, S.J., Perona-Wright, G., and MacDonald, A.S. (2008). Full development of Th2 immunity requires both innate and adaptive sources of CD154. J. Immunol. *180*, 8083–8092.

Jun, J.E., Wilson, L.E., Vinuesa, C.G., Lesage, S., Blery, M., Miosge, L.A., Cook, M.C., Kucharska, E.M., Hara, H., Penninger, J.M., et al. (2003). Identifying the MAGUK protein Carma-1 as a central regulator of humoral immune responses and atopy by genome-wide mouse mutagenesis. Immunity *18*, 751–762.

Kamata, I., Yamada, M., Uchikawa, R., Matsuda, S., and Arizono, N. (1995). Cysteine protease of the nematode Nippostrongylus brasiliensis preferentially evokes an IgE/IgG1 antibody response in rats. Clin. Exp. Immunol. *102*, 71–77.

Kopf, M., Le Gros, G., Bachmann, M., Lamers, M.C., Bluethmann, H., and Köhler, G. (1993). Disruption of the murine IL-4 gene blocks Th2 cytokine responses. Nature *362*, 245–248.

Liang, H.E., Reinhardt, R.L., Bando, J.K., Sullivan, B.M., Ho, I.C., and Locksley, R.M. (2012). Divergent expression patterns of IL-4 and IL-13 define unique functions in allergic immunity. Nat. Immunol. *13*, 58–66.

Mirchandani, A.S., Besnard, A.G., Yip, E., Scott, C., Bain, C.C., Cerovic, V., Salmond, R.J., and Liew, F.Y. (2014). Type 2 innate lymphoid cells drive CD4+ Th2 cell responses. J. Immunol. *192*, 2442–2448.

Mjösberg, J.M., Trifari, S., Crellin, N.K., Peters, C.P., van Drunen, C.M., Piet, B., Fokkens, W.J., Cupedo, T., and Spits, H. (2011). Human IL-25- and IL-33-responsive type 2 innate lymphoid cells are defined by expression of CRTH2 and CD161. Nat. Immunol. *12*, 1055–1062.

Monticelli, L.A., Sonnenberg, G.F., Abt, M.C., Alenghat, T., Ziegler, C.G., Doering, T.A., Angelosanto, J.M., Laidlaw, B.J., Yang, C.Y., Sathaliyawala, T., et al. (2011). Innate lymphoid cells promote lung-tissue homeostasis after infection with influenza virus. Nat. Immunol. *12*, 1045–1054.

Moro, K., Yamada, T., Tanabe, M., Takeuchi, T., Ikawa, T., Kawamoto, H., Furusawa, J., Ohtani, M., Fujii, H., and Koyasu, S. (2010). Innate production of T(H)2 cytokines by adipose tissue-associated c-Kit(+)Sca-1(+) lymphoid cells. Nature *463*, 540–544.

Neill, D.R., Wong, S.H., Bellosi, A., Flynn, R.J., Daly, M., Langford, T.K., Bucks, C., Kane, C.M., Fallon, P.G., Pannell, R., et al. (2010). Nuocytes represent a new innate effector leukocyte that mediates type-2 immunity. Nature *464*, 1367–1370.

Ohnmacht, C., Schwartz, C., Panzer, M., Schiedewitz, I., Naumann, R., and Voehringer, D. (2010). Basophils orchestrate chronic allergic dermatitis and protective immunity against helminths. Immunity *33*, 364–374.

Pape, K.A., Catron, D.M., Itano, A.A., and Jenkins, M.K. (2007). The humoral immune response is initiated in lymph nodes by B cells that acquire soluble antigen directly in the follicles. Immunity *26*, 491–502.

Perrigoue, J.G., Saenz, S.A., Siracusa, M.C., Allenspach, E.J., Taylor, B.C., Giacomin, P.R., Nair, M.G., Du, Y., Zaph, C., van Rooijen, N., et al. (2009). MHC class II-dependent basophil-CD4+ T cell interactions promote T(H)2 cytokine-dependent immunity. Nat. Immunol. *10*, 697–705.

Phythian-Adams, A.T., Cook, P.C., Lundie, R.J., Jones, L.H., Smith, K.A., Barr, T.A., Hochweller, K., Anderton, S.M., Hämmerling, G.J., Maizels, R.M., and MacDonald, A.S. (2010). CD11c depletion severely disrupts Th2 induction and development in vivo. J. Exp. Med. *207*, 2089–2096.

Price, A.E., Liang, H.E., Sullivan, B.M., Reinhardt, R.L., Eisley, C.J., Erle, D.J., and Locksley, R.M. (2010). Systemically dispersed innate IL-13-expressing cells in type 2 immunity. Proc. Natl. Acad. Sci. USA *107*, 11489–11494.

Roncarolo, M.G., Bigler, M., Haanen, J.B., Yssel, H., Bacchetta, R., de Vries, J.E., and Spits, H. (1991). Natural killer cell clones can efficiently process and present protein antigens. J. Immunol. *147*, 781–787.

Salimi, M., Barlow, J.L., Saunders, S.P., Xue, L., Gutowska-Owsiak, D., Wang, X., Huang, L.C., Johnson, D., Scanlon, S.T., McKenzie, A.N., et al. (2013). A role for IL-25 and IL-33-driven type-2 innate lymphoid cells in atopic dermatitis. J. Exp. Med. *210*, 2939–2950.

Santambrogio, L., Sato, A.K., Carven, G.J., Belyanskaya, S.L., Strominger, J.L., and Stern, L.J. (1999). Extracellular antigen processing and presentation by immature dendritic cells. Proc. Natl. Acad. Sci. USA *96*, 15056–15061.

Schlenner, S.M., Madan, V., Busch, K., Tietz, A., Läufle, C., Costa, C., Blum, C., Fehling, H.J., and Rodewald, H.R. (2010). Fate mapping reveals separate origins of T cells and myeloid lineages in the thymus. Immunity *32*, 426–436.

Seder, R.A., Germain, R.N., Linsley, P.S., and Paul, W.E. (1994). CD28-mediated costimulation of interleukin 2 (IL-2) production plays a critical role in T cell priming for IL-4 and interferon gamma production. J. Exp. Med. *179*, 299–304.

Serafini, N., Klein Wolterink, R.G., Satoh-Takayama, N., Xu, W., Vosshenrich, C.A., Hendriks, R.W., and Di Santo, J.P. (2014). Gata3 drives development of RORγt+ group 3 innate lymphoid cells. J. Exp. Med. *211*, 199–208.

Sokol, C.L., Chu, N.Q., Yu, S., Nish, S.A., Laufer, T.M., and Medzhitov, R. (2009). Basophils function as antigen-presenting cells for an allergen-induced T helper type 2 response. Nat. Immunol. *10*, 713–720.

Taieb, J., Chaput, N., Ménard, C., Apetoh, L., Ullrich, E., Bonmort, M., Péquignot, M., Casares, N., Terme, M., Flament, C., et al. (2006). A novel dendritic cell subset involved in tumor immunosurveillance. Nat. Med. *12*, 214–219.

Turner, J.E., Morrison, P.J., Wilhelm, C., Wilson, M., Ahlfors, H., Renauld, J.C., Panzer, U., Helmby, H., and Stockinger, B. (2013). IL-9-mediated survival of type 2 innate lymphoid cells promotes damage control in helminth-induced lung inflammation. J. Exp. Med. *210*, 2951–2965.

van Panhuys, N., Tang, S.C., Prout, M., Camberis, M., Scarlett, D., Roberts, J., Hu-Li, J., Paul, W.E., and Le Gros, G. (2008). In vivo studies fail to reveal a role for IL-4 or STAT6 signaling in Th2 lymphocyte differentiation. Proc. Natl. Acad. Sci. USA *105*, 12423–12428.

Voehringer, D., Reese, T.A., Huang, X., Shinkai, K., and Locksley, R.M. (2006). Type 2 immunity is controlled by IL-4/IL-13 expression in hematopoietic non-eosinophil cells of the innate immune system. J. Exp. Med. *203*, 1435–1446.

Wetzel, S.A., McKeithan, T.W., and Parker, D.C. (2005). Peptide-specific intercellular transfer of MHC class II to CD4+ T cells directly from the immunological synapse upon cellular dissociation. J. Immunol. *174*, 80–89.

Wong, S.H., Walker, J.A., Jolin, H.E., Drynan, L.F., Hams, E., Camelo, A., Barlow, J.L., Neill, D.R., Panova, V., Koch, U., et al. (2012). Transcription factor RORα is critical for nuocyte development. Nat. Immunol. *13*, 229–236.

Yamane, H., Zhu, J., and Paul, W.E. (2005). Independent roles for IL-2 and GATA-3 in stimulating naive CD4+ T cells to generate a Th2-inducing cytokine environment. J. Exp. Med. *202*, 793–804.

Yoshimoto, T., Yasuda, K., Tanaka, H., Nakahira, M., Imai, Y., Fujimori, Y., and Nakanishi, K. (2009). Basophils contribute to T(H)2-IgE responses in vivo via IL-4 production and presentation of peptide-MHC class II complexes to CD4+ T cells. Nat. Immunol. *10*, 706–712.

Zhu, J., Cote-Sierra, J., Guo, L., and Paul, W.E. (2003). Stat5 activation plays a critical role in Th2 differentiation. Immunity *19*, 739–748.

Immunity, Volume *41*

Supplemental Information

MHCII-Mediated Dialog between Group 2 Innate Lymphoid Cells and CD4$^+$ T Cells Potentiates Type 2 Immunity and Promotes Parasitic Helminth Expulsion

Christopher J. Oliphant, You Yi Hwang, Jennifer A. Walker, Maryam Salimi, See Heng Wong, James M. Brewer, Alexandros Englezakis, Jillian L. Barlow, Emily Hams, Seth T. Scanlon, Graham S. Ogg, Padraic G. Fallon, and Andrew N.J. McKenzie

Figure S1

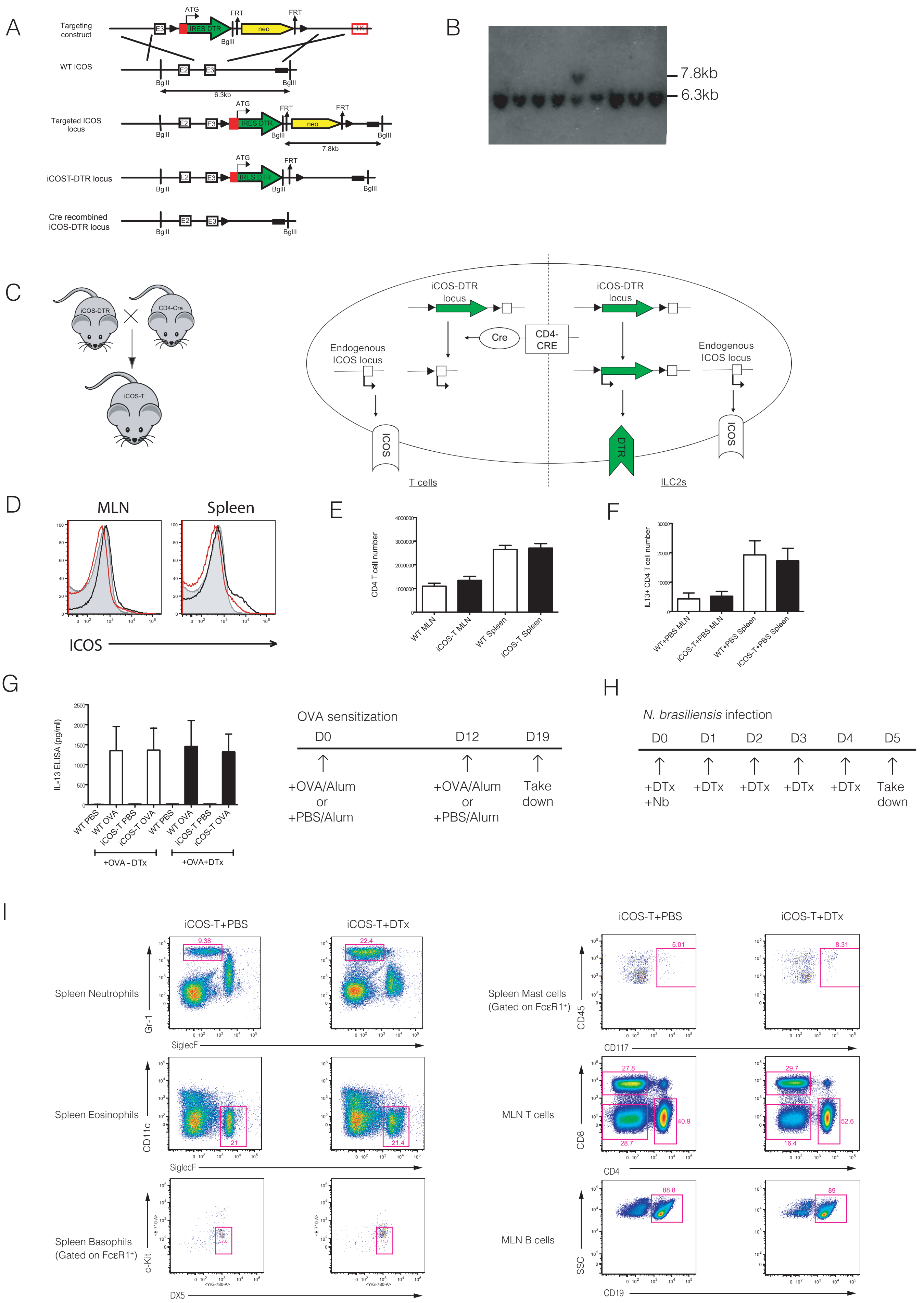
A
Targeting construct
WT ICOS
Targeted ICOS locus
iCOST-DTR locus
Cre recombined iCOS-DTR locus
ATG
FRT
IRES DTR
neo
BglII
E2
E3
6.3kb
7.8kb
B
7.8kb
6.3kb
C
iCOS-DTR
CD4-Cre
iCOS-T
iCOS-DTR locus
Cre
CD4-CRE
Endogenous ICOS locus
ICOS
DTR
T cells
ILC2s
D
MLN
Spleen
ICOS
E
CD4 T cell number
WT MLN
iCOS-T MLN
WT Spleen
iCOS-T Spleen
F
IL13+ CD4 T cell number
WT+PBS MLN
iCOS-T+PBS MLN
WT+PBS Spleen
iCOS-T+PBS Spleen
G
IL-13 ELISA (pg/ml)
WT PBS
WT OVA
iCOS-T PBS
iCOS-T OVA
+OVA - DTx
+OVA+DTx
OVA sensitization
D0
D12
D19
+OVA/Alum or +PBS/Alum
+OVA/Alum or +PBS/Alum
Take down
H
N. brasiliensis infection
D0
D1
D2
D3
D4
D5
+DTx +Nb
+DTx
+DTx
+DTx
+DTx
Take down
I
iCOS-T+PBS
iCOS-T+DTx
Spleen Neutrophils
Gr-1
SiglecF
9.38
22.4
Spleen Eosinophils
CD11c
SiglecF
21
21.4
Spleen Basophils (Gated on FcεR1⁺)
c-Kit
DX5
57.8
71.7
Spleen Mast cells (Gated on FcεR1⁺)
CD45
CD117
5.01
8.31
MLN T cells
CD8
CD4
27.8
28.7
40.9
29.7
16.4
52.6
MLN B cells
SSC
CD19
88.8
89

Figure S2

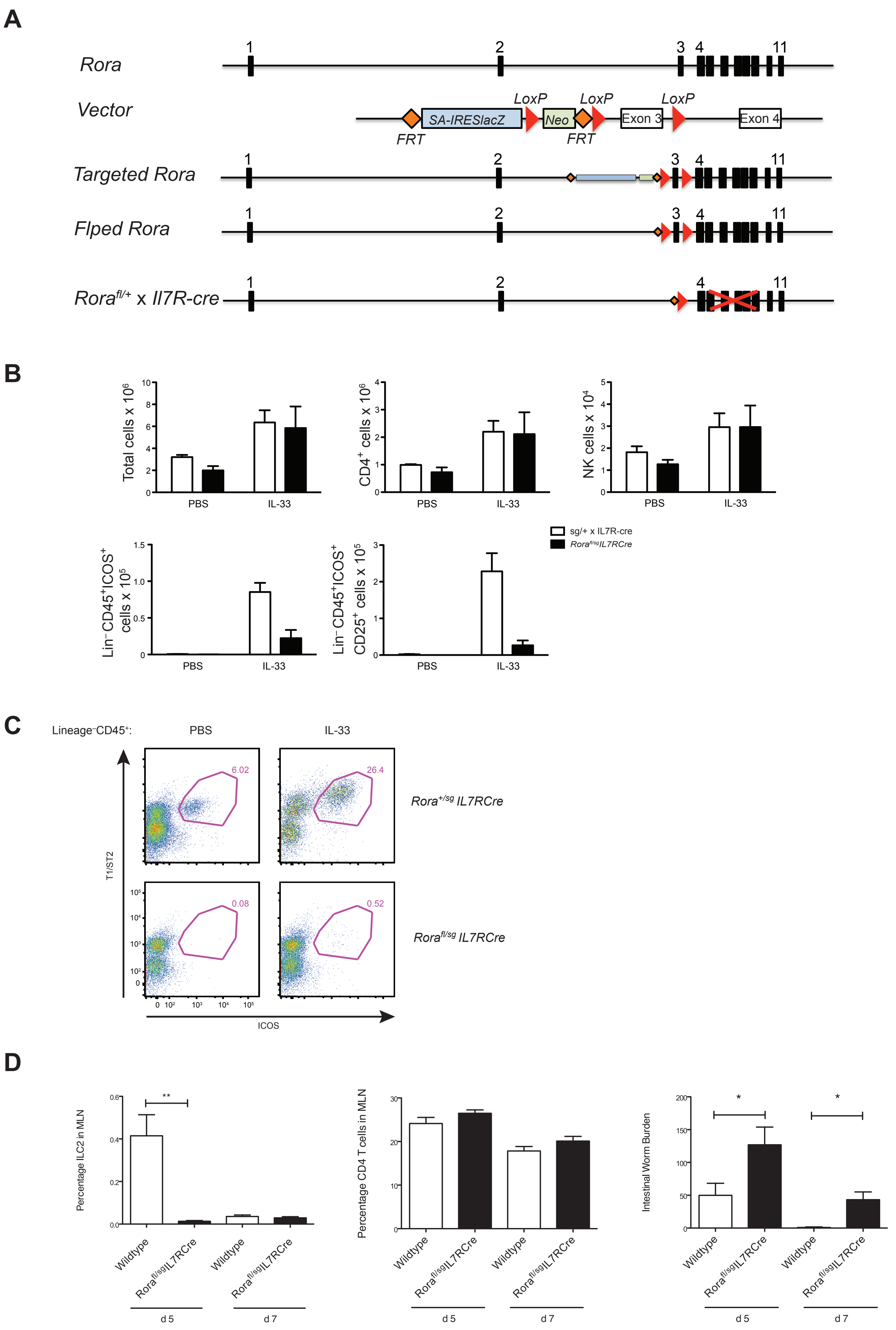

A
Rora
Vector
LoxP
SA-IRESlacZ
Neo
Exon 3
Exon 4
FRT
Targeted Rora
Flped Rora
Rora fl/+ x Il7R-cre
B
Total cells x 10^6
CD4+ cells x 10^6
NK cells x 10^4
PBS
IL-33
Lin−CD45+ICOS+ cells x 10^5
Lin−CD45+ICOS+ CD25+ cells x 10^5
sg/+ x IL7R-cre
Rora fl/sg IL7RCre
C
Lineage−CD45+:
6.02
26.4
0.08
0.52
Rora +/sg IL7RCre
Rora fl/sg IL7RCre
T1/ST2
ICOS
D
Percentage ILC2 in MLN
Percentage CD4 T cells in MLN
Intestinal Worm Burden
Wildtype
Rora fl/sg IL7RCre
d 5
d 7

Figure S3

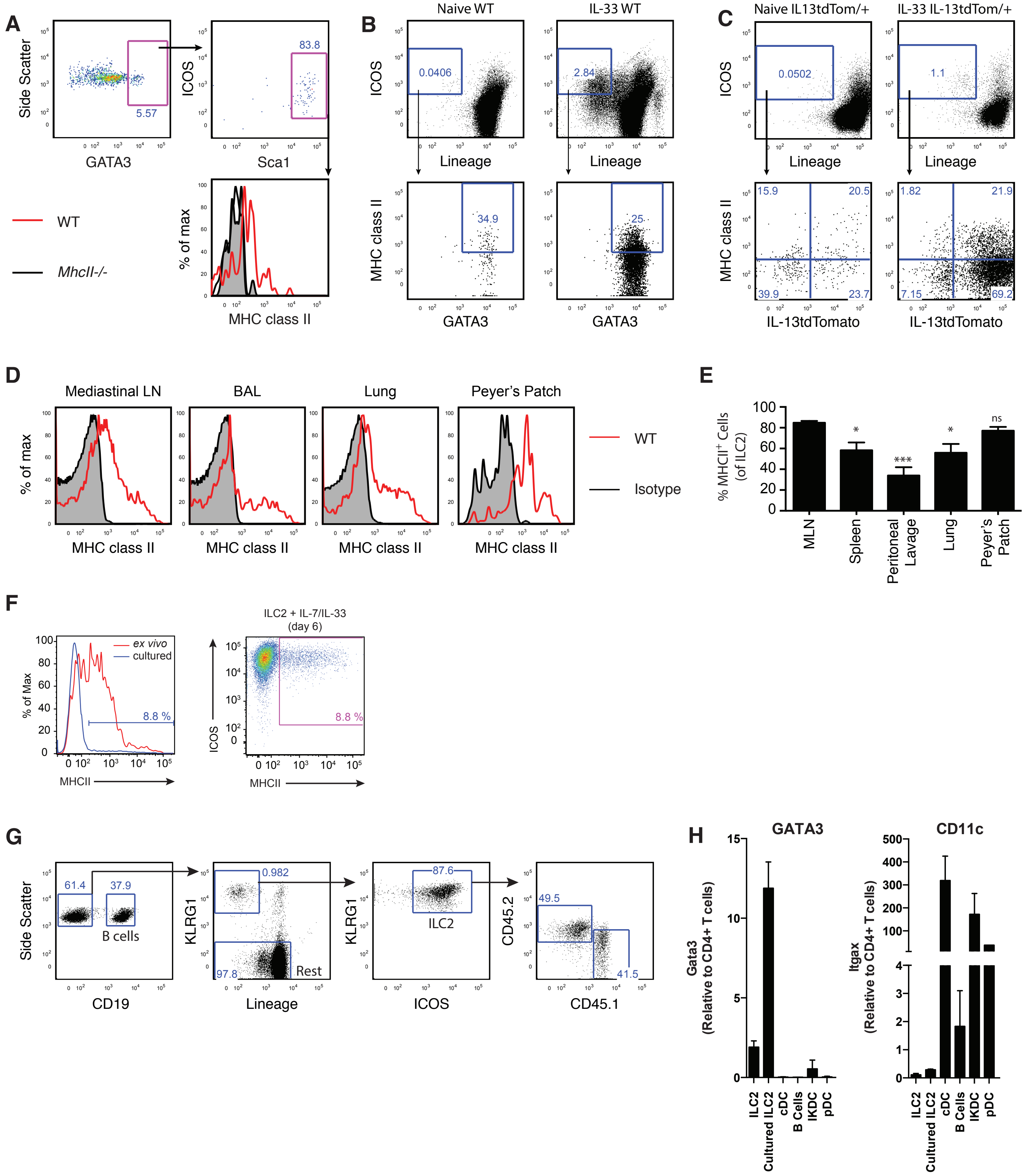

A
Side Scatter
GATA3
5.57
ICOS
Sca1
83.8
% of max
MHC class II
WT
MhcII-/-
B
Naive WT
IL-33 WT
ICOS
Lineage
0.0406
2.84
MHC class II
GATA3
34.9
25
C
Naive IL13tdTom/+
IL-33 IL-13tdTom/+
0.0502
1.1
15.9
20.5
39.9
23.7
1.82
21.9
7.15
69.2
IL-13tdTomato
D
Mediastinal LN
BAL
Lung
Peyer's Patch
Isotype
E
% MHCII+ Cells (of ILC2)
MLN
Spleen
Peritoneal Lavage
Peyer's Patch
ns
F
ILC2 + IL-7/IL-33 (day 6)
ex vivo
cultured
% of Max
MHCII
8.8 %
G
CD19
61.4
37.9
B cells
KLRG1
0.982
97.8
Rest
87.6
ILC2
CD45.2
CD45.1
49.5
41.5
H
GATA3
CD11c
Gata3 (Relative to CD4+ T cells)
Itgax (Relative to CD4+ T cells)
ILC2
Cultured ILC2
cDC
B Cells
IKDC
pDC

Figure S4

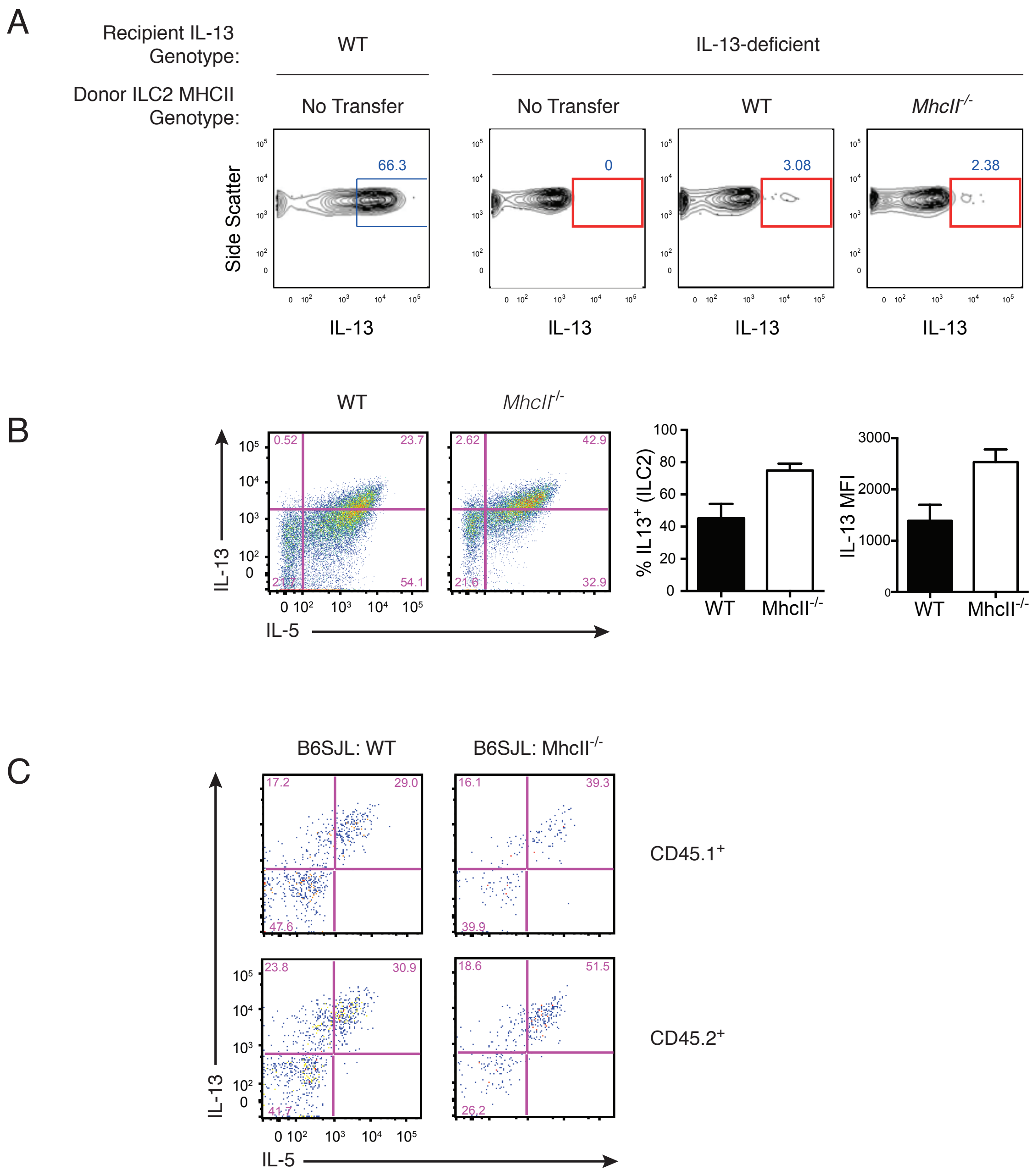
A
Recipient IL-13 Genotype:
WT
IL-13-deficient
Donor ILC2 MHCII Genotype:
No Transfer
No Transfer
WT
Mhcll-/-
Side Scatter
66.3
0
3.08
2.38
IL-13
B
WT
Mhcll-/-
0.52
23.7
54.1
2.62
42.9
21.6
32.9
IL-13
IL-5
% IL13+ (ILC2)
IL-13 MFI
WT
MhcII-/-
C
B6SJL: WT
B6SJL: MhcII-/-
17.2
29.0
47.6
16.1
39.3
39.9
23.8
30.9
41.7
18.6
51.5
26.2
CD45.1+
CD45.2+
IL-13
IL-5

Figure S5

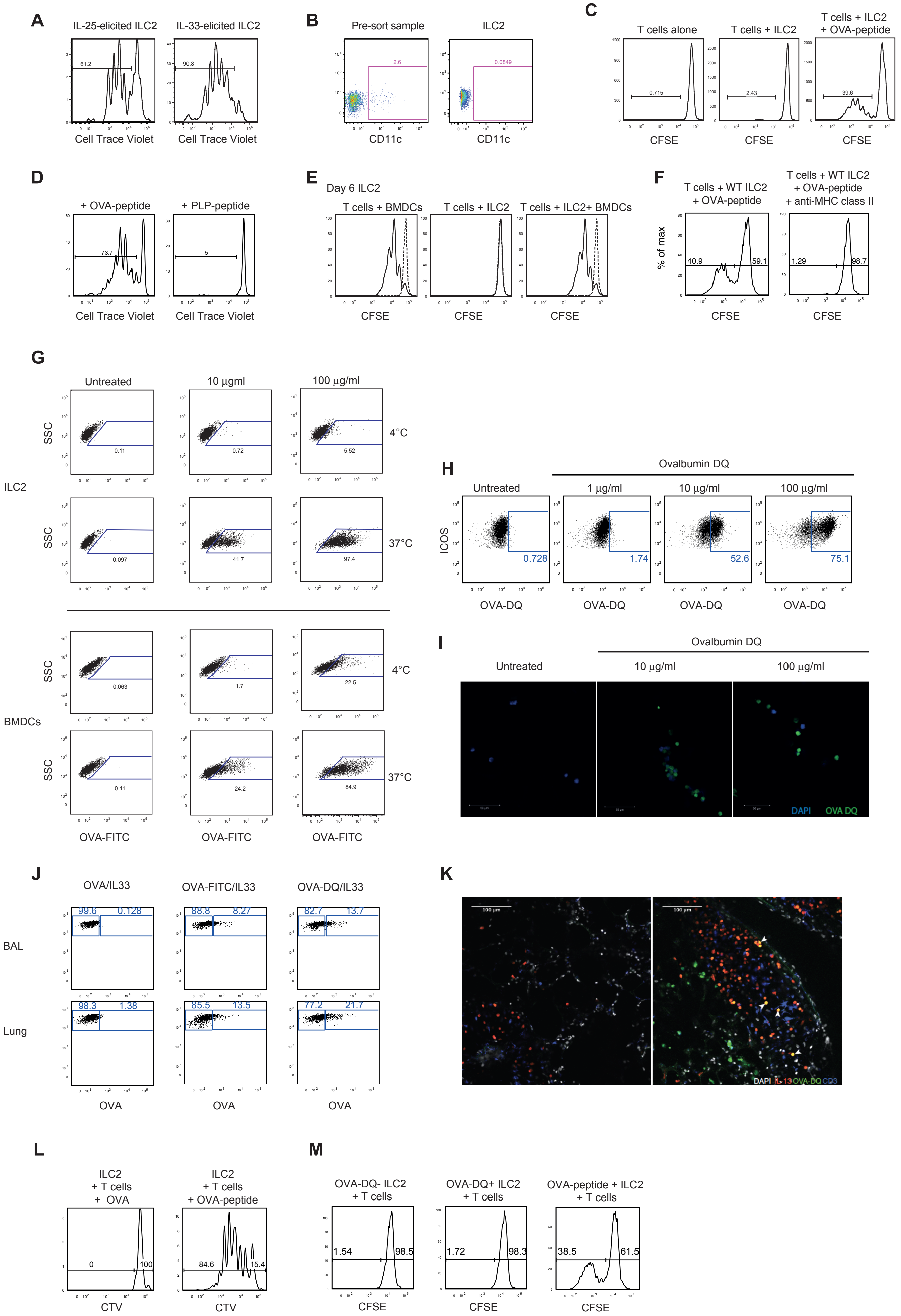
A
IL-25-elicited ILC2
IL-33-elicited ILC2
Cell Trace Violet
B
Pre-sort sample
ILC2
CD11c
C
T cells alone
T cells + ILC2
T cells + ILC2 + OVA-peptide
CFSE
D
+ OVA-peptide
+ PLP-peptide
Cell Trace Violet
E
Day 6 ILC2
T cells + BMDCs
T cells + ILC2
T cells + ILC2+ BMDCs
CFSE
F
T cells + WT ILC2 + OVA-peptide
T cells + WT ILC2 + OVA-peptide + anti-MHC class II
% of max
CFSE
G
Untreated
10 µgml
100 µg/ml
ILC2
BMDCs
SSC
4°C
37°C
OVA-FITC
H
Ovalbumin DQ
Untreated
1 µg/ml
10 µg/ml
100 µg/ml
ICOS
OVA-DQ
I
Ovalbumin DQ
Untreated
10 µg/ml
100 µg/ml
DAPI
OVA DQ
J
OVA/IL33
OVA-FITC/IL33
OVA-DQ/IL33
BAL
Lung
OVA
K
DAPI IL-13 OVA-DQ CD3
L
ILC2 + T cells + OVA
ILC2 + T cells + OVA-peptide
CTV
M
OVA-DQ- ILC2 + T cells
OVA-DQ+ ILC2 + T cells
OVA-peptide + ILC2 + T cells
CFSE

Figure S6

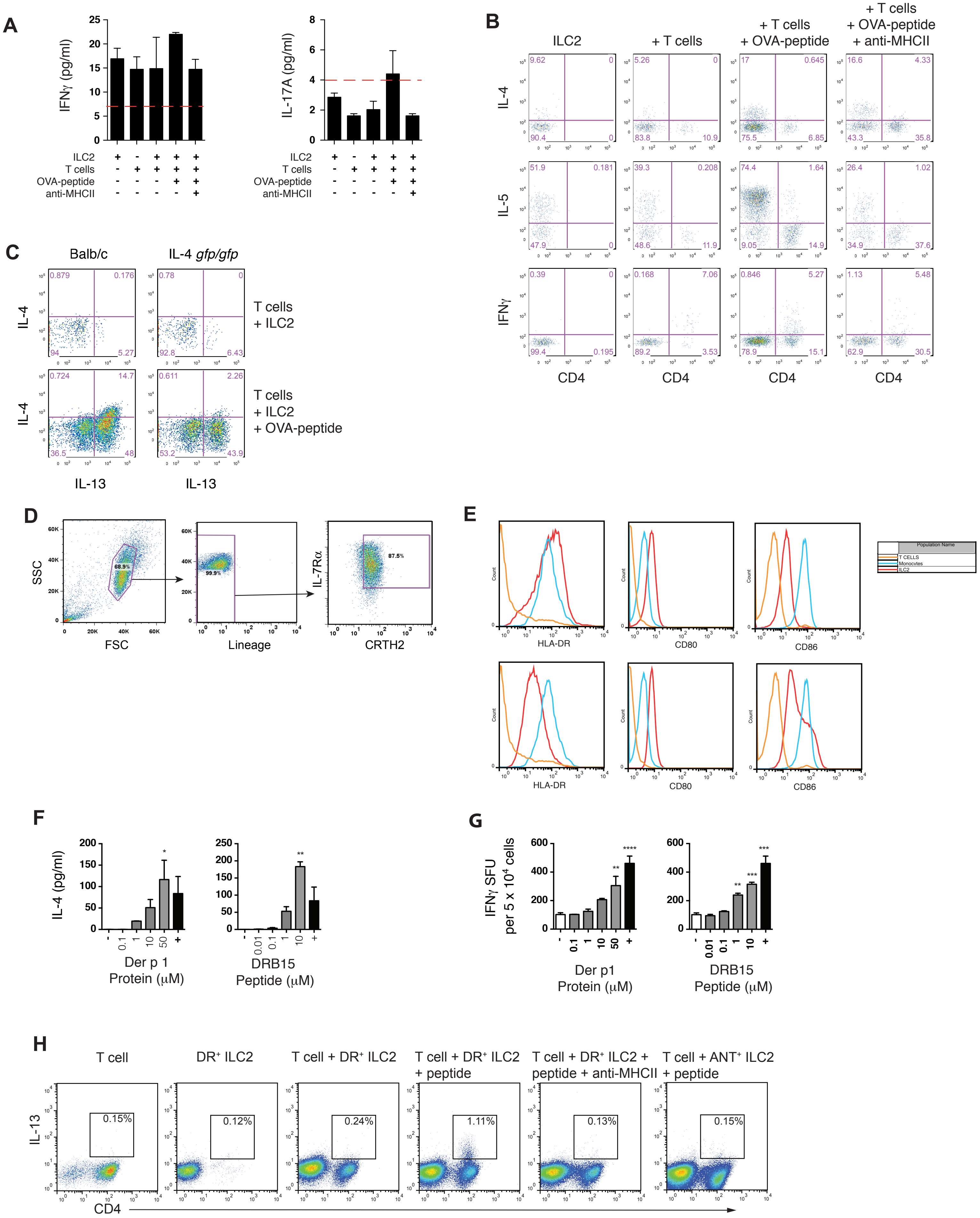

A
IFNγ (pg/ml)
IL-17A (pg/ml)
ILC2
T cells
OVA-peptide
anti-MHCII
B
ILC2
+ T cells
+ T cells + OVA-peptide
+ T cells + OVA-peptide + anti-MHCII
IL-4
IL-5
IFNγ
CD4
C
Balb/c
IL-4 gfp/gfp
T cells + ILC2
T cells + ILC2 + OVA-peptide
IL-13
D
SSC
FSC
Lineage
IL-7Rα
CRTH2
68.9%
99.9%
87.5%
E
HLA-DR
CD80
CD86
Count
Population Name
T CELLS
Monocytes
ILC2
F
IL-4 (pg/ml)
Der p 1 Protein (μM)
DRB15 Peptide (μM)
G
IFNγ SFU per 5 x 10^4 cells
Der p1 Protein (μM)
DRB15 Peptide (μM)
H
T cell
DR+ ILC2
T cell + DR+ ILC2
T cell + DR+ ILC2 + peptide
T cell + DR+ ILC2 + peptide + anti-MHCII
T cell + ANT+ ILC2 + peptide
IL-13
CD4
0.15%
0.12%
0.24%
1.11%
0.13%
0.15%

SUPPLEMENTAL FIGURE LEGENDS

Figure S1, related to Figure 1. Generation and analysis of iCOS-T mice

(A) Targeting strategy to insert the IRES-DTR fusion sequence directly after *Icos* exon 3.

(B) Southern blot screens for ICOS-DTR. A positive clone of iCOS-DTR digested with BglII. Transgenic fragment 7.8 kb, Wildtype (WT) fragment 6.3 kb.

(C) iCOS-DTR mice were crossed with CD4-Cre mice to generate iCOS-T mice. Schematic shows how the DTR gene is deleted from T cells and retained in ILC2.

(D) ICOS expression on $CD4^+$ T cells after 3 daily doses of IL-33 at 0.5 μg/dose. Shaded histogram indicates isotype control. Black histogram represents WT ICOS expression. Red histogram represents iCOS-T ICOS expression. n = 5.

(E) Total $CD4^+$ T cell numbers in WT and iCOS-T mice after 3 daily doses of IL-33 at 0.5 μg/dose. n = 3 mice (representative of two independent experiments).

(F) WT and iCOS-T mice were infected with *N. brasiliensis*. 5 days post infection (d.p.i.), MLN and spleen cells were taken and $IL\text{-}13^+CD4^+$ T cell number was determined by intracellular staining. n = 5 mice.

(G) WT and iCOS-T mice were sensitized intraperitoneally with either PBS/Alum or OVA/Alum (20 μg/dose) on day 0 and day 12. On day 19, splenocytes were harvested and plated in duplicate at 2.0×10^5 cells per well. Splenocytes were re-stimulated with OVA (100 μg/ml) *in vitro* in the presence or absence of DTx (100 ng/ml). Cultures were maintained for 3 days at 37°C. Supernatants were analysed for IL-13 by ELISA.

(H) Scheme for the administration of DTx (or PBS) to *N.brasiliensis*-infected iCOS-T mice.

(I) Flow cytometric analysis of myeloid and lymphoid cells from mice treated as depicted in (H) (5 d.p.i.).

Open bars represent wells treated with OVA and untreated with DTx. Black bars represent wells treated with both OVA and DTx. n = 5 mice. * P < 0.05. Bar graphs represent mean +/- s.e.m (E-G).

Figure S2, related to Figure 1. Generation and analysis of *$Rora^{sg/fl}$IL7RCre mice*

(A) *Rora*-targeted (*Rora*$^{tm1a(EUCOMM)Wtsi}$) embryonic stem cells were obtained from EUCOMM and used to produce *Rora*$^{fl/sg}$*IL7RCre*.

(B) Numbers of cells in the mesenteric lymph nodes of *Rora*$^{fl/sg}$*IL7RCre* mice following IL-33 administration.

(C) Detection of ILC2 precursors in the bone marrow of *Rora*$^{fl/sg}$*IL7RCre* mice following administration of PBS or IL-33.

(D) ILC2, CD4^{+} T cell and worm burdens in *Rora*$^{fl/sg}$*IL7RCre* mice following *N. brasiliensis* infection. Data are representative of a single experiment with 5 mice per group. Bar graphs represent mean +/- s.e.m (B and D).

Figure S3, related to Figure 2. ILC2 express MHCII

(A) MHCII expression by naïve wildtype or *MhcII*-/- Lin^{-}GATA3^{+}ICOS^{+}Sca1^{high} MLN ILC2 from untreated mice. Plots have been gated on lineage-negative cells.

(B-C) MHCII expression by GATA3^{+} (B) and IL-13^{+} (C) MLN ILC2 (Lin^{-}ICOS^{+}) from naïve or IL-33-treated wildtype (B) or IL-13-tdTomato reporter (C) mice.

(D) MHCII expression by wildtype ILC2 from the indicated tissues from IL-33-treated mice.

(E) Proportion of MHCII-positive ILC2 in the indicated tissues of naïve wildtype mice.

(F) MHCII expression *ex vivo* ILC2 or following culture for 6 days with IL-7 and IL-33.

(G) Gating strategy for MLN B cells and ILC2 from mixed chimeras reconstituted with 1:1 ratios of B6SJL and either C57Bl/6 or *MhcII*-/- bone marrow.

(H) Gene expression analysis for *Gata3* and *Itgax* from flow cytometrically sorted populations. IL-33 elicited ILC2 and B cells were from MLN. DC populations were from spleen. * P < 0.05, and *** *P* < 0.001; ns, not significant. Data are representative of 2 - 3 experiments with 2 - 5 mice per group (A-E). Bars represent mean +/- s.e.m.

Figure S4, related to Figure 3. Detection of transferred ILC2 in IL-13-deficient recipients

(A) Intracellular IL-13 staining of the ILC2 population (prior gating strategy Lin-CD4-Klrg1^{+}ICOS^{+}) in the MLN of *N. brasiliensis*-infected IL-13-deficient recipients. Transferred wildtype or *MhcII*-/- ILC2 are highlighted in red boxes. A *N. brasiliensis*-infected wildtype MLN not subjected to cell transfer was included as a positive control for IL-13 staining. Numbers in the outlined areas indicate the

percentage of IL-13$^+$ cells in each gate. Data are representative of four independent experiments with 5 - 6 mice per group.

(B) Intracellular IL-13 and IL-5 staining of ILC2 from IL-33-treated wildtype and MHCII-deficient mice. Bar graphs indicate the percentage of mesenteric lymph node ILC2 that express IL-13 and the mean fluorescence intensity (MFI) of IL-13 staining. Data are representative of 5 mice per group. Bars represent mean +/- s.e.m.

(C) Intracellular IL-13 and IL-5 staining of MLN ILC2 from chimeric mice, generated with a 1:1 ratio of B6SJL (CD45.1$^+$) bone marrow and either C57Bl/6 or *MhcII*-/- bone marrow (CD45.2$^+$). Mice were treated with IL-33.

Figure S5, related to Figure 4. ILC2 induce antigen-dependent T cell activation *in vitro*, and can process endocytosed OVA both *in vitro* and *in vivo*

(A) T cell stimulation with either IL-25 or IL-33 elicited ILC2.

(B) Purity of sorted ILC2 population (dot plot shows 10,000 events).

(C) Proliferation of CFSE-labelled DO11.10 CD4$^+$ T cells, induced by OVA-peptide pre-pulsed ILC2.

(D) Proliferation of CTV-labelled DO11.10 CD4$^+$ T cells in response to OVA-peptide (323-339) or an irrelevant peptide derived from a myelin proteolipid protein.

(E) Proliferation of CFSE-labelled DO11.10 CD4$^+$ T cells, induced by bone marrow-derived DCs (BMDCs) or ILC2, as indicated.

(F) OTIITg CD4$^+$ T cell proliferation as determined by Cell Trace Violet dilution for data in Figure 4E.

(G) *In vitro* endocytosis of fluorescently-labelled ovalbumin (OVA-FITC) by ILC2.

(H-I) Flow cytometry analysis (H) and micrograph (I) of fluorescence resulting from ovalbumin-DQ (OVA-DQ) degradation by ILC2.

(J) Identification of bronchoalveolar lavage and lung ILC2 containing degraded OVA-DQ following co-administration with IL-33 (intranasally). Plots are gated on Lin$^-$ICOS$^+$ cells.

(K) Immunofluorescence detection of OVA-DQ$^+$IL-13$^+$CD3$^-$ ILC2 (arrows) in the lung of IL-13-*tdTomato* reporter mice following intranasal administration of IL-33 and OVA-DQ.

(L-M) OTIITg CD4$^+$ T cell proliferation in the presence of OVA antigen-pulsed ILC2 (L) or FACs sorted OVA-DQ-loaded ILC2 (M).

Data are representative of two independent experiments. MLN ILC2 were elicited using IL-33 (B,D, F and L) or IL-25 (C, E, G, H and I).

Figure S6, related to Figure 5. MHCII-dependent ILC2/CD4$^+$ T cell interactions lead to type-2 cytokine expression

(A) IFNγ and IL-17A concentrations in supernatants following ILC2/OTIITg CD4$^+$ T cell co-cultures, as indicated. Bars represent mean +/- s.e.m and dotted line indicates the limit of detection.

(B) Flow cytometry analysis of intracellular cytokine staining of ILC2 and CD4$^+$ OTIITg T cells following co-cultures, as indicated.

(C) Intracellular IL-4 and IL-13 staining of wildtype, or IL-4-deficient (IL-4$^{gfp/gfp}$), ILC2 in antigen-dependent co-cultures, as indicated.

Data are representative of 2 - 3 independent experiments.

(D) Sort purity of human ILC2 (Lin$^-$IL7Ra$^+$CRTH2$^+$) used for *in vitro* co cultures.

(E) Comparison of HLA-DR, CD80, and CD86 expression on T cells, monocytes and ILC2 from two separate donors.

(F) IL-4 concentration in the supernatants following human ILC2:T cell co-culture in the presence of either Der p 1 or DRB15, or PMA and ionomycin (+).

(G) IFNγ expression in culture supernatants following ILC2:T cell co-cultures in the presence of Der p 1 protein, or peptide (DRB15), or PMA and ionomycin (+).

(H) Intracellular IL-13 staining of human CD4$^+$ T cells stimulated in the presence of Der p 1 peptide DRB15 with MHCII-matched DR$^+$ ILC2 or MHCII-mismatched ANT$^+$ ILC2. Data are from a single experiment.

Bar graphs represent mean +/- s.e.m. * $P < 0.05$, ** $P < 0.01$, *** $P < 0.001$ and **** $P < 0.0001$. Data are representative of 2 - 3 independent experiments (D-G) or a single experiment (H).

SUPPLEMENTAL EXPERIMENTAL PROCEDURES

Generation of iCOS-DTR and iCOS-T mice

The floxed IRES2-DTR gene cassette was generated in house. The IRES2 sequence was purchased from Clontech (pTRE3G-IRES Vector Set: Cat no. 631174). The IRES2 sequence was amplified by PCR to generate a product with an XhoI restriction enzyme site and a loxP site at the 5' end and a BclI restriction enzyme site at the 3' end. The diphtheria toxin receptor gene cassette created by See Heng Wong was amplified by PCR to generate a product with an XhoI restriction site at the 5' end, a BamHI restriction enzyme site at the ATG start codon and a loxP site at the 3' end. Both PCR products were sequentially cloned into the TOPO4 vector backbone to generate a floxed IRES2-DTR gene cassette that fused the start codon of the DTR gene with the internal start codon of the IRES2 sequence.

The iCOS-DTR mice were then generated by recombineering[1]. The floxed IRES2-DTR gene cassette had 50 base pair (bp) arms of homology for recombineering added to either ends by PCR primers (forward, 5'-TTGTGGTACTCCTTTTTGGATGCATACTTATCATCTGGTTTTCAAAAAGCGAATTACTCGAGATAACTTC-3', reverse, 5'-AAAAAAACACCTAGGAAAGAAAGTATAAGAAAAAAATAAAAATCACTTACTCTAGAACTAGTGGTTCCACC-3')

The floxed IRES2-DTR gene cassette was inserted immediately downstream of *Icos* exon 3 in the center of a 6kb length of genomic DNA. The *Icos* exon 3 genomic DNA was derived from the BAC bMQ310-P20 (Source Bioscience) by PCR (forward, 5'-AAATTTGCGGCCGCTTCTAACTCCTCCATTAGGGACCC-3', reverse, 5'-CAGGGGAACTAGTCCATGCGTTTC-3').

The iCOS-DTR construct was transfected into CCB embryonic stem cells and the clones were screened by Southern Blot analysis using *Bgl*II. The 3' screening probe was obtained by PCR of genomic DNA (5'- GGACCACAGGGCACCTGACTTG-3', 5'-GGCTGAGCTTCCTATTTGGAG-3'), yielding a probe of 616 bp. The expected wildtype band is 6.3 kb, while the expected target band is 7.8 kb. Targeted CCB

embryonic stem cell clones were used to generate an iCOS-DTR mouse line on a mixed background. The neomycin resistance gene was removed by breeding iCOS-DTR mice with FLP recombinase mice. Neomycin and FLP recombinase negative mice were then backcrossed 4 times to the C57Bl/6 background and subsequently crossed with CD4-Cre mice (Taconic, Nodel no. 4196) to generate iCOS-T mice. T cells in iCOS-T mice excise the DTR gene while ILC2 retain it. Genotyping of iCOS-T mice used PCR primers (5'-GACTCAGTGGCTATTCCGTCTACTTG-3', 5'-GGAAAATGCATGGTCTATCCTAGC-3', 5'-GGGAGAGGGGCATAACTTCGTATAGC-3'), giving a wildtype product of 562 bp and a targeted product of 452 bp. A separate Cre PCR was also used for genotyping (5'-TACCTGGCCTGGTCTGGACACAGTG-3', 5'-ATGGCTAATCGCCATCTTCCAGCAG-3').

Generation of *Rora*$^{+/flox}$*IL7RCre* mice

Rora-targeted (*Rora*$^{tm1a(EUCOMM)Wtsi}$) embryonic stem cells were obtained from EUCOMM and used to generate *Rora*-targeted mice from which the neomycin selection and βgal cassettes were removed by inter-crossing with Flp-recombinase mice to give *Rora*$^{fl/+}$ mice. *Rora*$^{fl/+}$ mice were crossed with *Rora*$^{sg/+}$*IL7RCre* mice to produce *Rora*$^{sg/fl}$*IL7RCre* and *Rora*$^{sg/+}$*IL7RCre* mice. Genotyping of *Rora*$^{fl/+}$ mice was undertaken using PCR primers (5'-TGAGTGGTAACACCACGGCACGC-3', 5'-TGGAGCAGAATCATCCAGGAGGCC-3', 5'-CAACGGGTTCTTCTGTTAGTCC-3'), giving wildtype product of 573 bp and a targeted product of ~650 bp.

Fluorescence-activated cell analysis and cell sorting

Mouse tissue cell suspensions were incubated with purified anti-Fc receptor blocking antibody (anti-CD16/CD32) before addition of the specific antibodies. Cell surface markers were stained using a combination of fluorescein isothiocyanate (FITC)-, phycoerythrin (PE)-, PE-Cy7-, PerCP-Cy5.5-conjugated, allophycocyanin-conjugated, Alexa Fluor 647–conjugated, eFluor® 660-conjugated, eFluor 450–conjugated, Brilliant Violet 421-conjugated, Brilliant Violet 510-conjugated, APC-Cy7-conjugated and biotin-conjugated monoclonal antibodies. For intracellular cytokine staining a Fix/Perm kit was used and was completed according to the manufacturer's protocols (BD Bioscience). Intracellular staining with anti-GATA3

(eBioscience) was stained following the FoxP3 Staining Buffer Set (eBioscience) and the manufacturer's instructions. Annexin V staining was performed according to the manufacturer's instructions with an Annexin V Apoptosis Detection Kit (eBioscience). In each experiment the appropriate isotype control monoclonal antibodies and single conjugate controls were also included. Samples were analysed using a Becton Dickinson LSRII or LSRFortessa™ flow cytometer running FACSDiva™ acquisition and analysed using FlowJo software (version 8.8.3, Tree Star). Cells were sorted using a Sony iCyt Synergy cell sorter.

Antibodies for flow cytometry analysis: primary antibodies anti-CD4 (GK1.5); anti-CD8a (53-6.7); anti-CD19 (1D3); anti-B220 (RA3-6B2); anti-Gr-1 (RB6-8C5); anti-CD11c (N418); anti-TER119 (TER-119); anti-CD25 (PC61.5); anti-CD44 (1M7); anti-CD45.1 (A20); anti-CD45.2 (104); anti-CD69 (H1.2F3); anti-NK1.1 (PK136); anti-TCRγδ (eBioGL3); anti-KLRG1 (2F1); anti-IL-4 (11B11); anti-IL-5 (TRFK5); anti-IL-13 (eBio13A); anti-MHCII (I-A/I-E) (M5/114.15.2); fixable viability dye eFluor-780 and unconjugated anti-CD16/32 (93) were purchased from eBioscience, UK. Streptavidin conjugated to PE, PE-Cy7 or AF647 were purchased from eBioscience. Anti-ICOS (C398.4A); anti-CD3e (145-2C11) ; anti-CD11b (M1/70) ; anti-IL-7Rα (SB/199) ; anti-FcεRIα (MAR-1) ; anti-CD80 (16-10A1) and anti-CD86 (GL-1) were purchased from Biolegend. Anti-CD5 (53-7.3) ; anti-IL-2 (JES6-5H4) ; anti-CD62L (MEL-14) and anti-TCRβ (H57-597) were purchased from BD Bioscience. Anti-T1/ST2 (DJ8) conjugated to biotin was purchased from MD Biosciences. All isotype control antibodies, rat anti-mouse IgG1, IgG2a, IgG2b and Armenian hamster anti-mouse IgG were purchased from eBioscience.

Enzyme-linked Immunosorbent Assay

Murine IL-4 and IL-5 ELISA were performed using purified anti-IL-4 (eBioscience) and anti-IL-5 (BD Biosciences). Briefly, culture supernatants were added to antibody coated Nunc-Immuno plates (Thermo Scientific) for 12 hours. Cytokine concentration was detected by sequential incubation with a biotinylated detection anti-cytokine antibody (IL-4 (BD Bioscience), and IL-5 (BD Bioscience), streptavidin-horseradish peroxidase and an HRP colorimetric reagent. Murine IL-13 was detected with Quantikine IL-13 (R&D Systems) or mouse IL-13 ELISA Ready-

Set-Go! (eBioscience). Human IL-4 ELISA (eBioscience) were performed according to the manufacturer's protocol. Supernatant was added to anti-IL-4 coated Nunc-Immuno plate (Thermo Scientific) for 24 hours. Cytokine assays were also performed using the MagPix® system according to standard protocols.

CFSE and Cell trace Violet Staining

Cells were stained with Cell Trace™ CFSE or Cell Trace™ Violet Cell Proliferation Kits (Invitrogen) according to the manufacturer's instructions at a final concentration of 5 μM.

Bone Marrow-Derived DC (BMDC) Cultures

BMDCs were generated as previously described from the femurs and tibias of wildtype mice (Lutz et al., 1999) using 20 ng/ml recombinant mouse GM-CSF (R & D Systems).

***In vitro* OVA-FITC and OVA-DQ Uptake Assays**

For the *in vitro* OVA-FITC uptake assays BMDC or IL-7/IL-33 cultured ILC2 were incubated for 4 hours with culture medium alone or in the presence of FITC-conjugated OVA (10 μg/ml or 100 μg /ml) at either 4°C or 37°C. OVA-DQ endocytosis and degradation assays were performed by incubating DQ-conjugated OVA (1-100 μg/ml) with freshly isolated ILC2 for 16 hours at 37°C, 5% CO_2. Cytospins of OVA-DQ treated ILC2 were performed by centrifuging samples in a Shandon Cytospin 3 (Shandon) at 900 rpm for 10 minutes and subsequently analysed for fluorescence on a Carl-Zeiss inverted microscope (LSM 710) and processed with ZEN 2008 (Carl-Zeiss).

Immunofluorescence of OVA-DQ Uptake Assays

For immunofluorescence micrographs, $IL13^{+/tdTomato}$ mice were treated with OVA-DQ and IL-33 i.n, and lungs were then prepared as described previously (Scanlon et al., 2011). After blocking, sections were incubated with purified Armenian hamster anti-mouse CD3ε (Clone 145-2C11, 1.25 μg/ml) (BioLegend) for 1 h, sections were incubated with Alexa Fluor 647-conjugated goat anti-Armenian hamster antibody (3 μg/ml) (Jackson Immunoresearch) and 300 nM DAPI (Biotium) for 30 min. Sections

were then mounted with ProLong Gold antifade reagent (Invitrogen) according to manufacturer's instructions. Sections were imaged with an LSM 710 confocal scanner (Zeiss) mounted on an Axio Observer.Z1 microscope (Zeiss) equipped with an EC Plan-NEOFLUAR 20X/0.5 objective (Zeiss). Data were recorded using Zen imaging software (Zeiss) and then analysed using ImageJ software (National Institutes of Health).

Quantitative Reverse Transcriptase PCR

APC populations and ILC2 were purified (by fluorescence-activated cell sorting) from the mesenteric lymph nodes of naïve mice or those challenged with three daily intraperitoneal injections of 0.5 μg recombinant mouse IL-33, respectively. RNA was purified and reverse-transcribed. Taqman primer and probe sets (Applied Biosystems) were used for quantification of the expression of *H2-Aa* (Mm00439211_m1), *H2-Ab1* (Mm00439216_m1), *H2-Eb1* (Mm00439221_m1), *Ciita* (Mm00482914_m1), *CD74* (Mm00658576_m1), *Itgax* (Mm00498698_m1) and *Gata3* (Mm00484683_m1). Expression was quantified relative to that of *Gapdh*.

Human ILC2 and T cell purification and stimulation

The ILC2 population and $CD4^+$ T cell lines were generated from the peripheral blood of HLA-DRB1*15-positive donors. For the isolation of ILC2, PBMC were fluorescently labelled using the lineage markers; CD3 (SK7; BD Biosciences), CD19 (SJ25C1; BD Biosciences), CD123 (FAB301C; R&D systems), CD11b (DCIS1/18; Biolegend), CD11c (BU15; Abcam), CD8 (RPA-T8; Biolegend), FcεRI (AER-37 (CRA-1); Biolegend), CD14 (MφP9; BD Biosciences), CD4 (MEM-241; Abcam), CD56 (HCD56; Biolegend). The negative population that express CD45 (HI30; Biolegend), CRTH2 (BM16; Miltenyibiotec), IL-7Rα (A019D5; Biolegend) were purity sorted from the lymphoid population on a MoFlo™ XDP cell sorter into 96 well plates and cultured with 100 IU/ml IL-2 (Peprotech 200-02) and Gamma-irradiated peripheral blood mononuclear cells (PBMCs) from 3 healthy volunteers (2 x 10^6 cells/ml). Cultures were stained with HLA-DR (BD Biosciences, G46-6), CD80 (Biolegend, 2D10) and CD86 (BD Biosciences, 2331) antibodies and acquired using Summit software on a CyAn flow Cytometer. FlowJo and Summit software were used for further data analysis.

PBMC were separated from heparinized whole blood on Lymphoprep™ (Axis-Shield PoC AS, Oslo, Norway) washed and resuspended in RPMI 1640 (Gibco®) supplemented with 2 mM L-glutamine, 50 U/ml penicillin, 50 μg/ml streptomycin and 10% Human Serum (R10H). Using the known sequence for Der p 1 (swissprot: locus DERP1_DERPT, accession P08176, recombinant protein Indoor Biotechnologies Ltd, USA), relevant HLA-DRB1*1501 peptide (Ardern-Jones et al., 2007) (AVNIVGYSNAQGVDY), was constructed in house using F-moc chemistry and purity confirmed using HPLC. Peptides were added each at a final concentration of 4 μM. LoTox™ Recombinant Der p 1 deglycosylated (LTR-DP1D-1) was obtained from Indoor Biotechnologies Ltd, USA and was protease-deficient in the absence of DTT (not added). The cells were incubated at 37°C with 5% CO2. At days 3 and 7 IL-2 was added to a final concentration of 100 IU/ml. At day 10 the cells were removed from the plate, washed twice in sterile PBS and returned overnight to unused wells in R10H. At Day 11 the cells were then used in ELISpot analyses as below at 4×10^4/well.

ELISpot analyses

ELISpot analyses were performed as previously described (Lalvani et al., 1997). T cell lines input cell numbers were 4×10^4/well. The plates were incubated overnight at 37°C and 5% CO_2 and were developed with streptavidin-alkaline phosphatase (Mabtech AB) and AP conjugate substrate kit (BioRad).

HLA typing

Donors were tissue typed by PCR-SSP phototyping (Bunce et al., 1995).

SUPPLEMENTAL REFERENCES

Ardern-Jones, M.R., Black, A.P., Bateman, E.A., and Ogg, G.S. (2007). Bacterial superantigen facilitates epithelial presentation of allergen to T helper 2 cells. Proc Natl Acad Sci U S A 104, 5557-5562.

Bunce, M., O'Neill, C.M., Barnardo, M.C., Krausa, P., Browning, M.J., Morris, P.J., and Welsh, K.I. (1995). Phototyping: comprehensive DNA typing for HLA-A, B, C, DRB1, DRB3, DRB4, DRB5 & DQB1 by PCR with 144 primer mixes utilizing sequence-specific primers (PCR-SSP). Tissue Antigens 46, 355-367.

Lalvani, A., Brookes, R., Hambleton, S., Britton, W.J., Hill, A.V., and McMichael, A.J. (1997). Rapid effector function in CD8+ memory T cells. J Exp Med 186, 859-865.

Liu, P., Jenkins, N.A., and Copeland, N.G. (2003). A highly efficient recombineering-based method for generating conditional knockout mutations. Genome Res 13, 476-484.

Lutz, M.B., Kukutsch, N., Ogilvie, A.L., Rossner, S., Koch, F., Romani, N., and Schuler, G. (1999). An advanced culture method for generating large quantities of highly pure dendritic cells from mouse bone marrow. J Immunol Methods 223, 77-92.

Scanlon, S.T., Thomas, S.Y., Ferreira, C.M., Bai, L., Krausz, T., Savage, P.B., and Bendelac, A. (2011). Airborne lipid antigens mobilize resident intravascular NKT cells to induce allergic airway inflammation. J Exp Med 208, 2113-2124.
